# New information on *Bonapartenykus* (Alvarezsauridae: Theropoda) from the Allen Formation (middle Campanian-lower Maastrichtian) of Río Negro Province, Patagonia, Argentina clarifies the Patagonykinae body plan

**DOI:** 10.1371/journal.pone.0308366

**Published:** 2025-01-30

**Authors:** Jorge Gustavo Meso, Jonah Nathaniel Choiniere, Mattia Antonio Baiano, Stephen Louis Brusatte, Juan Ignacio Canale, Leonardo Salgado, Diego Pol, Michael Pittman

**Affiliations:** 1 Universidad Nacional de Río Negro (UNRN), Instituto de Investigación en Paleobiología y Geología (IIPG), General Roca Río Negro, Argentina; 2 Consejo Nacional de Investigaciones Científicas y Técnicas (CONICET), Buenos Aires, Argentina; 3 Evolutionary Studies Institute, University of the Witwatersrand, Johannesburg, South Africa; 4 Museo “Carmen Funes”, Plaza Huincul, Neuquén, Argentina; 5 Área Laboratorio e Investigación, Museo Municipal “Ernesto Bachmann”, Villa El Chocón, Neuquén, Argentina; 6 School of GeoSciences, University of Edinburgh, Edinburgh, United Kingdom; 7 Museo Paleontológico Egidio Feruglio, Trelew, Chubut, Argentina; 8 School of Life Sciences, The Chinese University of Hong Kong, Shatin, Hong Kong, SAR, China; Universita degli Studi della Basilicata, ITALY

## Abstract

Alvarezsauria is a group of morphologically distinctive, medium- to small-sized later-diverging coelurosaurian theropod dinosaurs, whose record ranges from the Late Jurassic to the Late Cretaceous. This clade had a widespread distribution in Laurasia in what is now Europe, Asia, and North America, although there are also several Cretaceous taxa from Gondwana in what is now Argentina that all belong to the family Alvarezsauridae. Although alvarezsaurid taxonomic diversity and anatomical knowledge has expanded over the last decade, alvarezsaurid internal phylogenetic relationships remain highly debated. In this contribution, we describe new alvarezsaurid material and review previously reported alvarezsaurid specimens from the middle Campanian—lower Maastrichtian Allen Formation discovered at the Salitral Ojo de Agua locality in Río Negro province, Patagonia, Argentina. Although these specimens were collected at different times without precise original provenance information, here we gathered X-ray diffraction data of the associated sediments that now suggests that all of these specimens come from the same site and stratigraphic level as the holotype of the alvarezsaurid *Bonapartenykus ultimus*. Based on this new provenance information and the morphological similarity of the considered specimens, here we tentatively refer them to the genus *Bonapartenykus*. These newly referred specimens add to the record of this genus and shed light on the body plan of the alvarezsaurid clade Patagonykinae by permitting a more complete reconstruction of the neck, pectoral girdle, hindlimb, and tail.

## 1. Introduction

Alvarezsauria is a group of later-diverging coelurosaurian theropod dinosaurs of medium to small size, whose fossil record spans from the Late Jurassic to the Late Cretaceous. The distribution of this clade was likely originally Laurasian since its earliest known members are currently restricted to the Oxfordian (Late Jurassic) of China [1–3]. The Early Cretaceous fossil record of Alvarezsauria was also limited to the Asian continent, which now consists of up to three taxa [4–6]. By the Late Cretaceous, Alvarezsauria was highly diversified and globally distributed, with abundant Laurasian records from what is now Asia [7–21], Europe ([22]; see also [23] and references therein), and North America [24–27] as well as the first Gondwanan records represented by discoveries from South America [28–34].

The first South American species to be described was *Alvararezsaurus calvoi*, a small theropod from the Bajo de la Carpa Formation (Santonian) of Neuquén Province, Argentina [28]. This taxon led José Fernando Bonaparte to erect the groups Alvarezsauria and Alvarezsauridae in which other taxa were later included. Since then, four other alvarezsaurian species from Patagonia have been described: *Alnashetri cerropoliciensis* from Cenomanian outcrops of the Candeleros Formation, Neuquén Group [34, 35], *Patagonykus puertai* from Turonian-Coniacian outcrops of the Portezuelo Formation, Neuquén Group [29, 36], *Achillesaurus manazzonei* from Santonian outcrops of the Bajo de la Carpa Formation, Neuquén Group [31], and *Bonapartenykus ultimus* from middle Campanian—lower Maastrichtian outcrops of the Allen Formation, Malargüe Group [33].

Despite the increase of alvarezsaurian specimens described in the last decade and the new palaeocological inferences that have been made [37–43], phylogenetic relationships between members of the group remain highly debated (e.g., [3, 6, 10, 11, 13, 26, 33, 41, 44, 45]). In fact, the only two points of agreement among all previous phylogenetic analyses of Alvarezsauria are the early diverging positions of the Late Jurassic taxa from China (*Haplocheirus*, *Aorun*, *Shishugounykus*) and the monophyly of the Late Cretaceous Asian clade Parvicursorinae (e.g., [3, 6, 10, 13, 26, 44, 46]). Patagonykinae [33] is the only named South American clade of alvarezsaurids that clusters *Patagonykus* and *Bonapartenykus* and has been recovered in some studies [41, 46–48] but not others [3, 6].

This contribution focuses on the stratigraphically youngest patagonykine, *Bonapartenykus* from the Allen Formation of Argentina (middle Campanian—lower Maastrichtian, Upper Cretaceous). This formation has yielded numerous dinosaur remains [49–57] and is the only unit that has produced alvarezsaurid remains from the Campanian—Maastrichtian of Patagonia. Here we describe new alvarezsaurid materials and review all specimens catalogued under the MPCN-PV 738 (partially described by [32, 58]; see below Historical Background) collected at the Salitral Ojo de Agua locality, which are here tentatively referred to *Bonapartenykus ultimus*. We then discuss the significance of our results to our current understanding of the patagonykine body plan.

### 1.1. Institutional abbreviations

**MPCA**, Museo Provincial “Carlos Ameghino”, Cipolletti, Río Negro Province, Patagonia, Argentina; **MGPIFD-GR**, Museo de Paleontología y Geología del Instituto de Formación Docente Continua de General Roca, General Roca, Río Negro Province, Argentina; **MPCN-PV**, Museo Patagónico de Ciencias Naturales “Juan Carlos Salgado”, General Roca, Rio Negro Province, Argentina; **MACN-PV-RN**, Museo Argentino de Ciencias Naturales “Bernardino Rivadavia”, Buenos Aires Province, Argentina; **MCF PVPH**, Museo “Carmen Funes”, Plaza Huincul, Neuquén Province, Argentina; **MUCPv**, Museo de la Universidad del Comahue, Centro Paleontológico “Lago Barreales”, Neuquén Province, Argentina; **MEPyG**, Museo Educativo de Paleontología y Geología del Instituto de Formación Docente Continua de General Roca, General Roca, Río Negro Province, Argentina.

## 2. Material and methods

### 2.1. Historical background

Powell [50] was the first to report the presence of small theropods at the Salitral Ojo de Agua (SOA) locality in stratigraphic levels corresponding to the Allen Formation. Powell (op. cit.) reported osteological remains of these dinosaurs associated with eggs and eggshells assigned to titanosaurian sauropods and theropods. Among the theropod oological material, Powell (op. cit.) reported numerous eggshells and two partial eggs of a type characterised by thin eggshell and external ornamentation consisting of low and short ridges.

In December 2003 and February 2004, Leonardo Salgado (who was part of the expeditions led by J.E. Powell to SOA) conducted two successive field trips to SOA, expanding some of Powell’s original fossiliferous quarries, including the one that yielded the small theropod bones and eggshells. These new field trips resulted in abundant dinosaur bones and eggs, most of which come from Powell’s small theropod site, renamed as “Arriagada III” quarry [54, 59, 60].

From the “Arriagada III” quarry, [32] described several theropod bones under the collection number MGPIFD-GR 166–194, assigning them to Alvarezsauridae. These specimens were transferred to the Museo Patagónico de Ciencias Naturales (MPCN) once this institution was created and are currently catalogued under MPCN-PV 738. In the same publication, the intimate association between the alvarezsaurian bones and fossil eggshells was mentioned for the first time. Additionally, other fragmentary alvarezsaurid materials from “Arriagada III” quarry were collected and communicated by [61] but were never described, and were added to the set of remains accessioned under MPCN-PV 738.

In 2006, Agnolín and collaborators reported the alvarezsaurian affinities of theropod remains collected by J.E. Powell at SOA and reported in 1992. Six years later, these remains were described in detail as the holotype (MPCA 1290) of a new alvarezsaurid species: *Bonapartenykus ultimus* [33]. In this contribution, they referred to this species three specimens collected by Salgado and his team. Two of them were described by Salgado (op. cit.; MGPIFD-GR 166 and MGPIFD-GR 184), whereas the femur collected at the same site was described earlier by [58] as an ornithopod (MGPIFD-GR 177, labelled by [58] as MEPyG 177).

In summary, the alvarezsaurid materials collected from SOA currently include the holotype of *Bonapartenykus ultimus* (MPCA 1290) and a large number of elements collected at the same quarry and, presumably, from the same level (egg level 2) that are now accessioned under the collection number MPCN-PV 738. These remains were reported or partially described by [32, 33, 58, 61], but have never been fully described or analysed. The lack of detailed treatment of these materials (which include over 70 elements) motivates this study, which will describe them in detail, evaluate their taxonomic affinities and discuss their implications for understanding the biology and evolution of alvarezsaurids.

### 2.2. Descriptions and comparison

For the description and general orientation of the materials as well as the analysis of anatomical characters, we adopted the terminology used in avian anatomy [62]. In the particular case of the anatomical descriptions of the vertebra laminae and fossae, the terminology employed was that proposed by [63–65].

Concerning the description of each vertebral element, a reference was made to the region they occupy within the sequence, *i*.*e*., anterior cervical, posterior cervical, anterior dorsal, posterior dorsal, sacral, anterior caudal, middle caudal and posterior caudal. As stated by [66], although this division is arbitrary, it is very useful when describing general anatomical features, especially when analysing anatomical characters. In addition, we use the terms “cranial” and “caudal” instead of “anterior” and “posterior” in the case of postcranial elements. In order to avoid confusion, exceptions were made for the location of each vertebral element. For example, instead of using “caudal cervical vertebrae”, “posterior cervical vertebrae” was used. The presumed orientation of the scapula in life was oblique to the sagittal plane [67, 68]. For directional adjectives, unless explicitly stated otherwise, we use “long/short” to describe variation in the craniocaudal direction, “wide/narrow” to describe variation in the mediolateral direction, and “high/low” to describe variation in the dorsoventral direction.

Measurements were taken with a pair of hardened stainless steel digital calipers that could measure up to 150 mm (6 "), with an accuracy of 0.01 mm.

### 2.3. Phylogenetic nomenclature and analisys

Following the suggestion made by [39], we adopt the stem-based definition of Alvarezsauria of [33], as the most inclusive clade including *Alvarezsaurus calvoi* but not *Passer domesticus* or *Ornithomimus velox*; Alvarezsauridae [28] as the least inclusive clade containing *Alvarezsaurus calvoi*, *Mononykus olecranus*, and their most recent common ancestor; Parvicursorinae [8] *sensu* [69] as a stem-based taxon, and defined as the most inclusive clade including *Parvicursor remotus* but not *Patagonykus puertai*; and Mononykini as the group of theropods that include taxa more closely related to *Mononykus* than to *Parvicursor*, *Patagonykus*, and *Alvarezsaurus* [33].

The phylogenetic position of the studied material (MPCN-PV 738) was assessed using a modified version of the phylogenetic data matrix presented by [6] and later papers (e.g., [3, 10, 13, 26]). This modified phylogenetic data matrix coding 647 characters for 122 terminal taxa was assembled in WinClada v. 1.00.08 [70] and analysed using TNT v. 1.5 [71]. 54 new phenotypic characters were added, including 4 of our own based on observed differences in Patagonian alvarezsaurids (see S1 File: “1. Characters”). Character 431 was eliminated from the original phylogenetic data matrix of [6] as we considered it to be ambiguously defined. We also, modified the definition of character 619 and 363 character scorings (see S1 File: “1. Characters”, “2. Modifications” and “3. Characters ordered”). Thus, the current study includes virtually all known alvarezsaurian material, except for the following specimens that were not included due to their fragmentary nature and/or undescribed status: the specimens LACM 153311 [32]; IVPP V20341 [21], and 41HIII-0104 [11]. The Patagonian alvarezsaurid *Alnashetri* was not included in this analysis given that a new specimen is currently under study (P. Makovicky pers. comm.) that is larger and more complete than the holotype and modifies the information available for this taxon. The protocol for the phylogenetic consisted of a combination of the tree-search algorithms including Wagner trees, TBR branch swapping, and sectorial searches until 100 hits of the same minimum tree length were obtained (command “xmult = hits 100”). The resultant trees were then subjected to a final round of TBR branch swapping (command "bb") to ensure a thorough sampling of tree space. We also implemented the IterPCR procedure of [72], which generates a reduced consensus without wildcards. The robustness of each node was obtained from the script pcrjak.run [73], which aims to identify taxa that decrease support values because they are unstable in the jackknife analysis.

### 2.4. X-ray diffraction (XRD)

One of the authors of this work (L.S.) was involved in the original collection of both the holotype of *Bonapartenykus ultimus* (MPCA 1290) as well as most of the specimens accessioned under MPCN-PV 738, so we have first-hand information that indicates that all of the specimens were from the same quarry (Arriagada III). To test this whether all of the specimens belong to the same stratigraphic level (“egg level 2” of [32]), we carried out an X-ray diffraction analysis of the mineralogical composition of the sedimentary rock matrix attached to the fossils of MPCA 1290 and MPCN-PV 738.

We used a Rigaku SmartLab 3 X-ray diffractometer housed at the Instituto de Investigación en Paleobiología y Geología (UNRN-CONICET) in General Roca, Argentina. Two types of preparations were necessary for the matrix in MPCA 1290 and MPCN-PV 738, the first one involved preparing a total rock powder, and the second one involved separation of the sample’s clay content.

To prepare the total rock powder, a representative subsample of the matrix of MPCA 1290 and MPCN-PV 738 was ground manually in an agate mortar, until reaching a 200 ASTM mesh size, equivalent to 74 μm. The resulting material was evenly distributed in a sample holder and analysed in the XRD. It is worth mentioning that the distribution of the sample on the sample holder was carried out in such a way that the crystals were randomly oriented, allowing them to be exposed to the greatest possible number of X-rays.

To separate the clay content from the matrix of MPCA 1290 and MPCN-PV 738, a representative subsample of approx. 15g was used. This was placed in a Falcon-type tube with 40 ml of distilled water and stirred for 40 minutes, then the suspension containing the clay fraction of less than 2 μm was removed by pipetting. One millilitre of this suspension was spread on a glass slide and allowed to dry in air at room temperature (AD) for 24 hours before being analysed by XRD. This procedure ensures extended sedimentation of the clay crystals preferentially on their basal faces once the distilled water evaporates, which allows correct identification. After drying the sample, it was subjected to the same smear for 12 hours, solvation with ethylene glycol, and then analysed in the XRD. Finally, the clay smear was calcined at 550ºC and re-analysed by XRD.

## 3. Systematic paleontology

Theropoda Marsh, 1881.

Coelurosauria Huene, 1920.

Alvarezsauria Bonaparte, 1991.

Alvarezsauridae Bonaparte, 1991.

Patagonykinae Agnolín, Powell, Novas, Kundrát, 2012.

cf. *Bonapartenykus ultimus* Agnolín, Powell, Novas, Kundrát, 2012.

### 3.1. Referred specimens

Fragment of a middle cervical neural arch (MPCN-PV 738.26); almost complete middle cervical neural arch (MPCN-PV 738.27); fragment of a cervical neural arch (MPCN -PV 738.28); fragment of a cervical centrum (MPCN-PV 738.32); fragment of a cervical neural arch (MPCN-PV 738.43); neural arch fragment of a proximal cervical vertebra (MPCN-PV 738.47); fragment of a cervical postzygapophysis (MPCN-PV 738.48); fragment of a cervical postzygapophysis (MPCN-PV 738.50); cervical prezygapophysis (MPCN-PV 738.51); neural arch fragment of a cervical vertebra (MPCN-PV 738.52); fragment of a cervical postzygapophysis (MPCN-PV 738.55); two almost complete cervico-dorsal neural arch (MPCN-PV 738.15–16); almost complete dorsal rib (MPCN-PV 738.36); proximal end of a dorsal rib (MPCN-PV 738.35); fragment of a dorsal postzygapophysis (MPCN-PV 738.49); two incomplete fused sacral centra (MPCN-PV 738.14; formerly MGPIFD-GR 171); fragment of a possible last sacral vertebra (MPCN-PV 738.31); caudal centrum (MPCN-PV 738.11); almost complete proximal caudal centrum (MPCN-PV 738.10); caudal prezygapophysis (MPCN-PV 738.9); distal caudal centrum (MPCN-PV 738.12); almost complete mid caudal vertebra (MPCN-PV 738.8); distal fragment of a caudal centrum (MPCN-PV 738.38); proximal fragment of a caudal centrum (MPCN-PV 738.37); almost complete caudal centrum (MPCN-PV 738.29); distal fragment of a caudal centrum (MPCN-PV 738.30); distal fragment of a caudal centrum (MPCN-PV 738.40); almost complete caudal neural arch (MPCN-PV 738.58); proximal end of a chevron (MPCN-PV 738.7); proximal end of a left scapula (MPCN-PV 738.3; formerly MGPIFD-GR 184); almost complete left coracoid (MPCN-PV 738.2); partial left pubis (MPCN-PV 738.4; MGPIFD-GR 194); almost complete right femur (MPCN-PV 738.1; formerly MEPyG 177); proximal end of a right fibula (MPCN-PV 738.13); fragment of a right tibia (MPCN-PV 738.24); distal end of a metatarsal I (MPCN-PV 738.6); proximal end of a right metatarsal II (MPCN-PV 738.5); fragment of a right metatarsal III (MPCN-PV 738.25); fragment of a left metatarsal III (MPCN-PV 738.34); fragment of a left metatarsal III (MPCN-PV 738.33); left pedal phalanx III-2 (MPCN-PV 738.18); right pedal phalanx IV-3 (MPCN-PV 738.19); right pedal phalanx IV-4 (MPCN-PV 738.21); left pedal phalanx IV-2 (MPCN-PV 738.23); left pedal phalanx IV-4 (MPCN- PV 738.20); proximal fragment of a right pedal phalanx III-1 (MPCN-PV 738.22); proximal fragment of an indeterminate pedal non-ungual phalanx (MPCN-PV 738.39); proximal fragment of an indeterminate pedal non-ungual phalanx (MPCN-PV 738.41); fragment of an indeterminate pedal non-ungual phalanx (MPCN-PV 738.17); almost complete pedal ungual phalanx IV (MPCN-PV 738.44); almost complete pedal ungual phalanx III (MPCN-PV 738.45); pedal ungual phalanx II (MPCN-PV 738.46); proximal fragment of indeterminate pedal ungual phalanx (MPCN-PV 738.54); and several indeterminate fragments.

### 3.2. Locality and horizon

**Fig 1:**
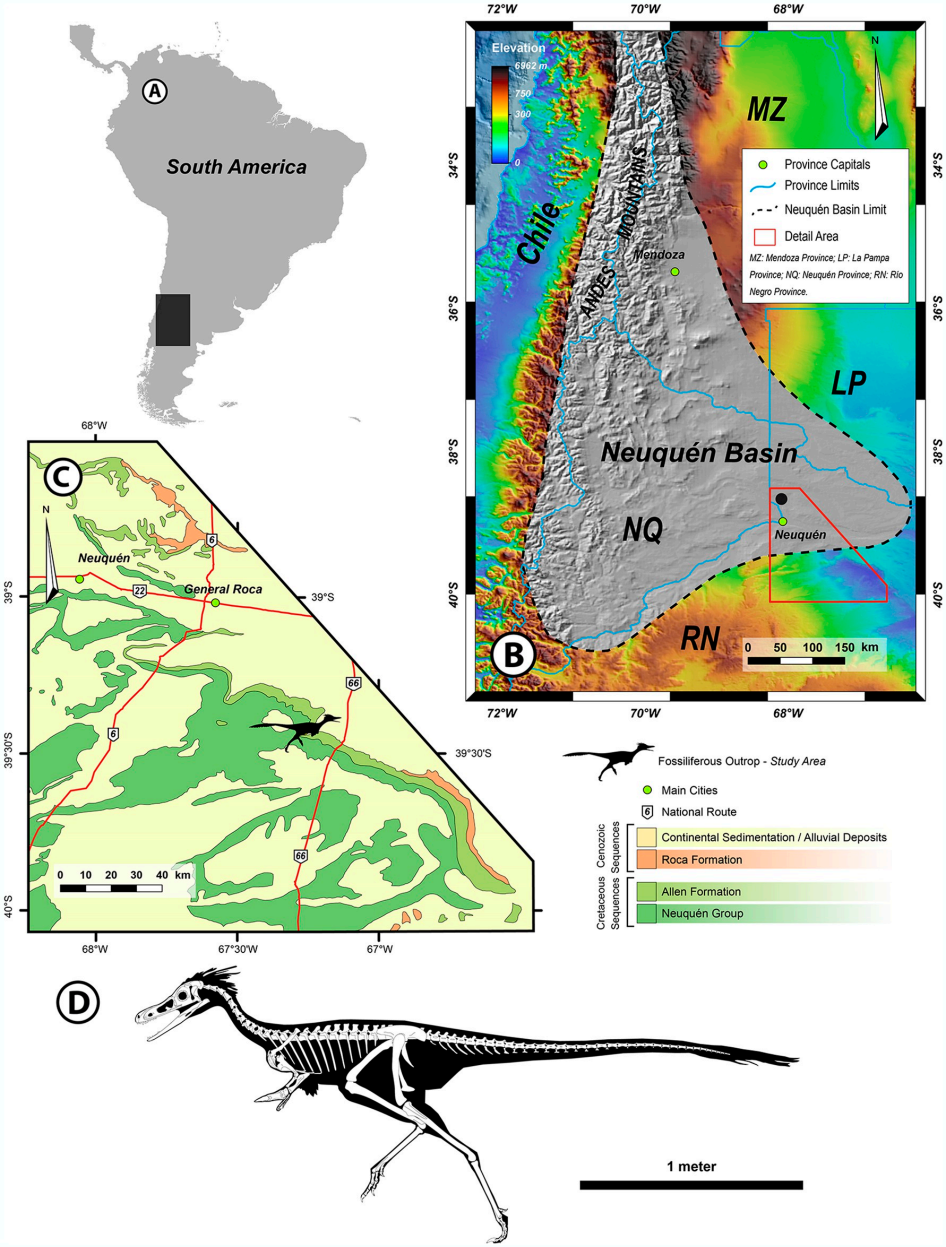
Location of the MPCN-PV 738 locality. A, South America showing the extent of the Cuenca Neuquina (Neuquén Basin) in Argentina. B, Cuenca Neuquina showing the location of the MPCN-PV 738 locality in Río Negro province. C, generalised stratigraphic section outcrop of the Allen and others formations in the area of discovery. D, skeletal reconstruction of MPCN-PV 738.

“Arriagada III” (39°25’55” S; 67°17’40” O) at the Salitral Ojo de Agua locality of Río Negro Province, Argentina. The quarry area is no more than 30–40 m^2^. The horizon is “Egg level 2” within the middle Campanian-lower Maastrichtian Allen Formation [32] (Fig 1).

### 3.3. Taxonomic assignment of MPCN-PV 738

The materials gathered under MPCN-PV 738 that were not described by [32], exhibit characters that allow their assignment to Alvarezsauridae: cervical vertebrae with craniocaudally short and dorsoventrally low neural spines; last sacral vertebrae procoelous with a centrum transversely compressed and sharply keeled ventrally; procoelous caudal vertebrae; coracoid without bicipital tubercle; glenoid cavity laterocaudally oriented; pubic apron reduced to a tiny ridge; pubis with a strongly laterally compressed proximal end [29, 36, 74]. Moreover, some of this material (MPCN-PV 738.2) shows unambiguous synapomorphies of Patagonykinae: the presence of a longitudinal ridge on the lateral surface of coracoid; ventral half of the coracoid strongly sculptured; ventral half of the coracoid medially deflected [33]. Some specimens (MPCN-PV 738.2-3-49) exhibit autapomorphies of *Bonapartenykus ultimus*: ventral portion of coracoid decorated with delicate but profuse grooves (convergent with *Xixianykus*); scapula with a wide notch on the posterior margin of the bone [33].

## 4. Results

### 4.1. XRD study of specimen matrix

The alvarezsaurid specimens collected by Salgado and his crew (both published and unpublished) and the holotype of *Bonapartenykus ultimus* come from the same quarry “Arriagada III”, but our XRD analysis aimed to test if the latter also comes from the “egg level 2” of [32]. This involved analysis of the total rock powder and clay fraction of the sedimentary rock matrix associated with the fossils of MPCA 1290 and MPCN-PV 738 (S2 File: “S1–S4 Figs”) (see Methodology). Macroscopic study of both samples with a binocular microscope indicates that the fossil-bearing sedimentary rock matrix is a loosely consolidated fine-medium quartz sandstone, with moderate selection and roundness. However, XRD analysis of the clay samples revealed diffractograms that show characteristic peaks compatible with an expandable clay of the smectite family, differing only in the type of feldspar and plagioclase mineral species.

### 4.2. Morphological description

#### 4.2.1. Axial skeleton

**Fig 2:**
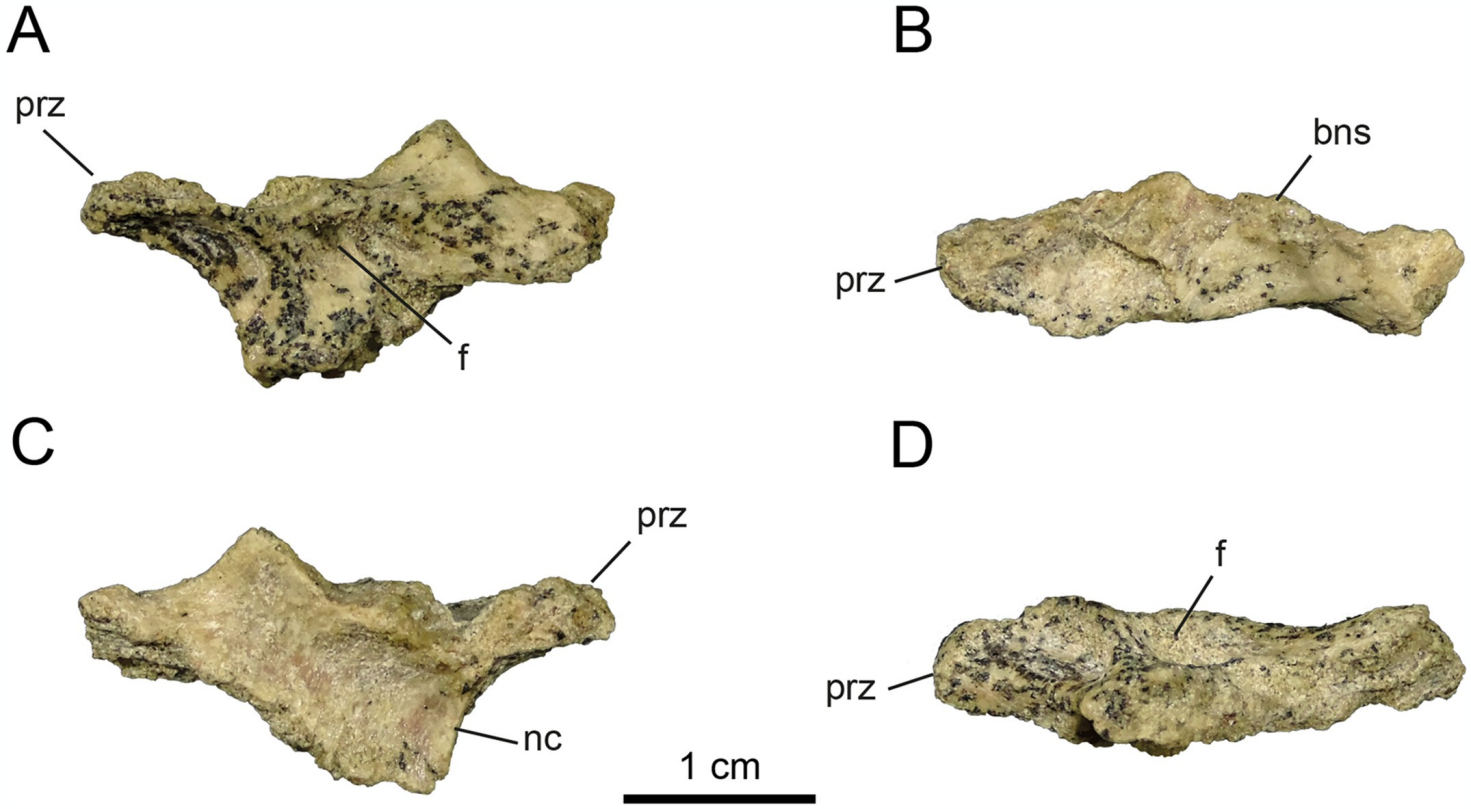
Anterior cervical vertebrae of MPCN-PV 738.47. A, lateral view; B, dorsal view; C, medial view; D, ventral view. Abbreviations: bns, base of neural arch; prz, prezygapophysis; nc, neural channel; f, fossa. Scale bar equal to 1 cm.

**Fig 3:**
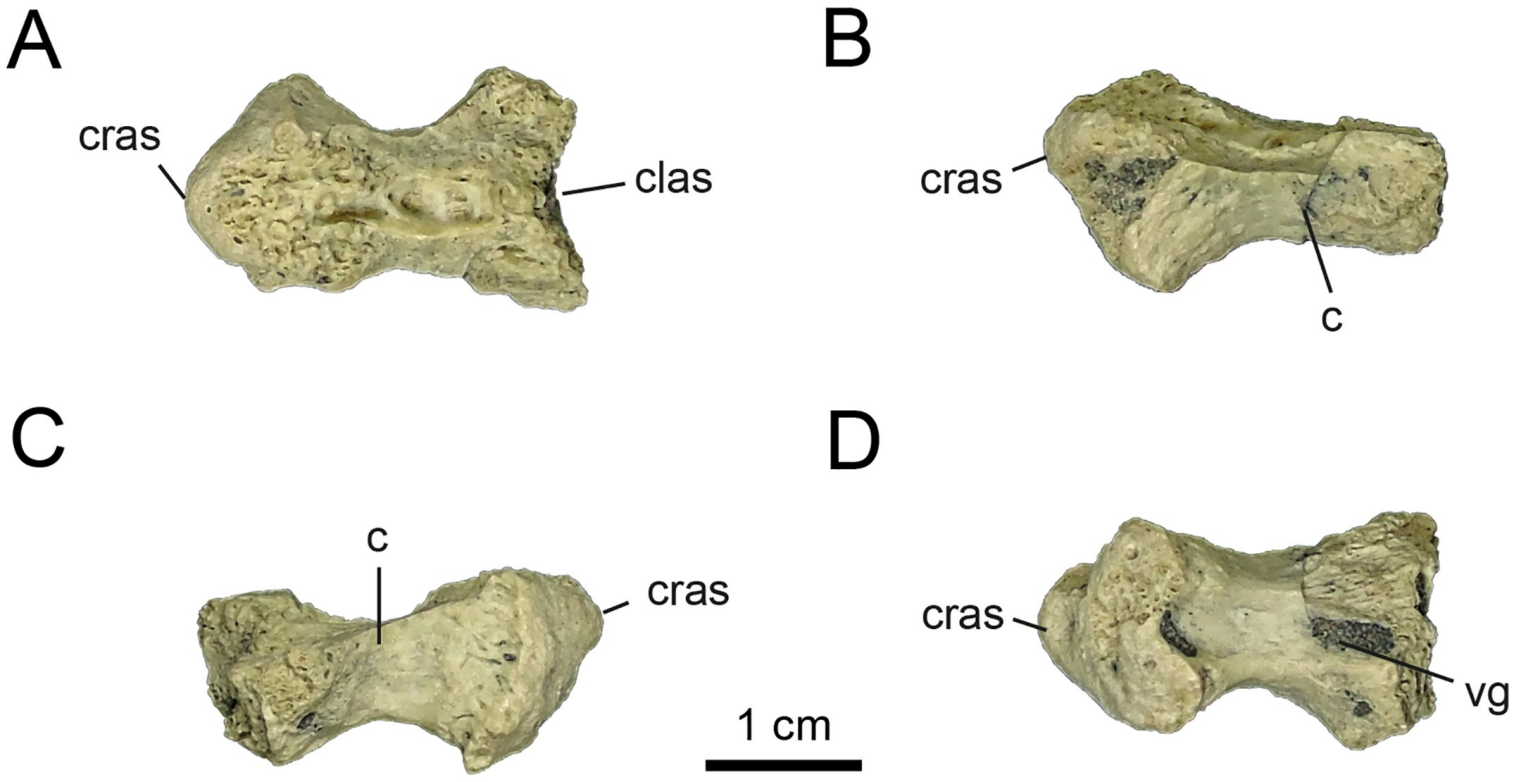
Anterior cervical vertebrae of MPCN-PV 738.32. A, dorsal view; B, left lateral view; C, right lateral view; D, ventral view. Abbreviations: cras, cranial articular surface; clas, caudal articular surface, vg, ventral groove. Scale bar equal to 1 cm.

**Fig 4:**
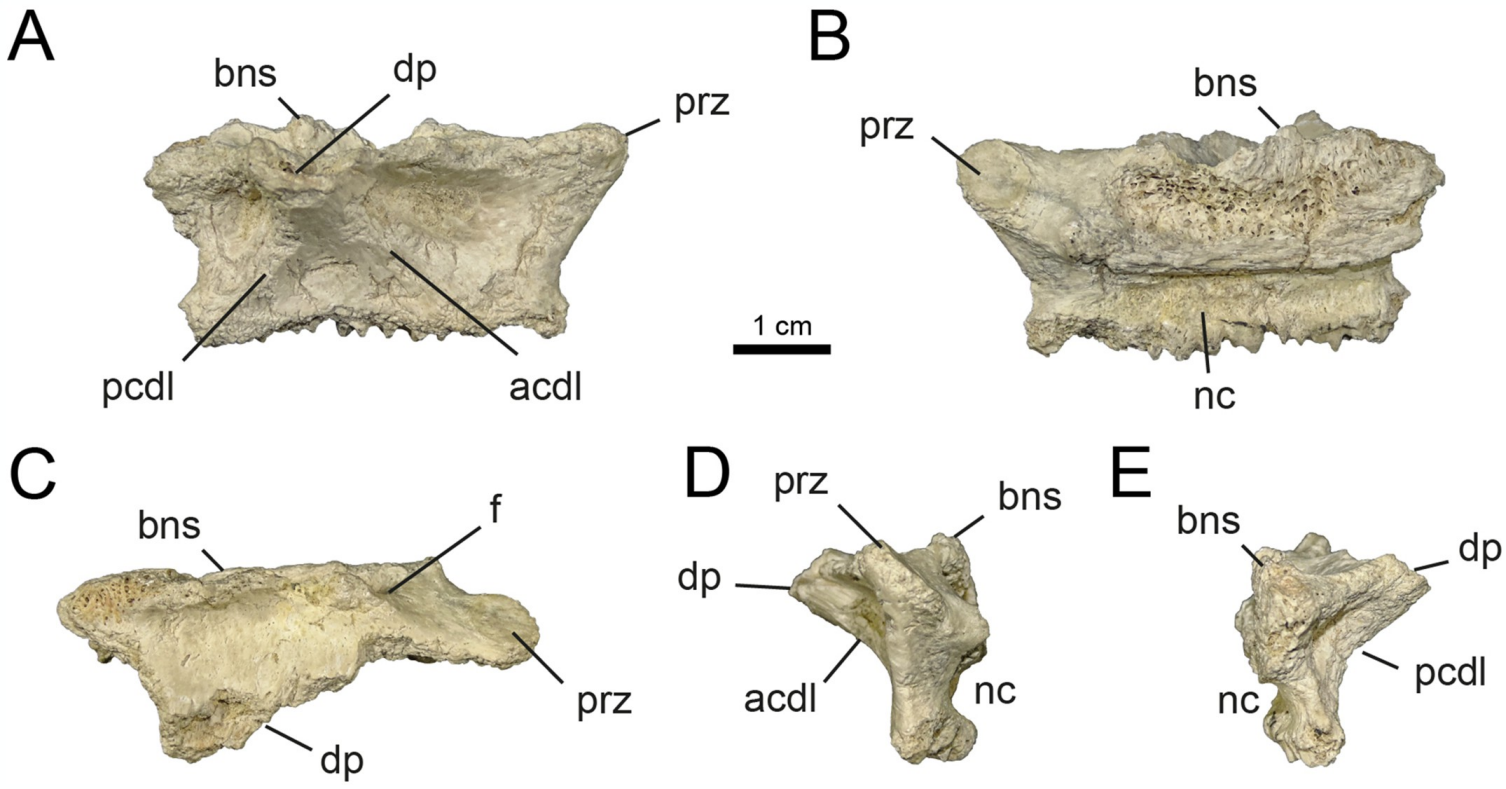
Middle cervical vertebrae of MPCN-PV 738.28. A, lateral view; B, medial view; C, dorsal view; D, cranial view; E, caudal view. Abbreviations: pcdl, posterior centrodiapophyseal lamina; acdl, anterior centrodiapophyseal lamina; bns, base neural spine; prz, prezygapophysis; dp, diapophysis; f, fossa. Scale bar equal to 1 cm.

**Fig 5:**
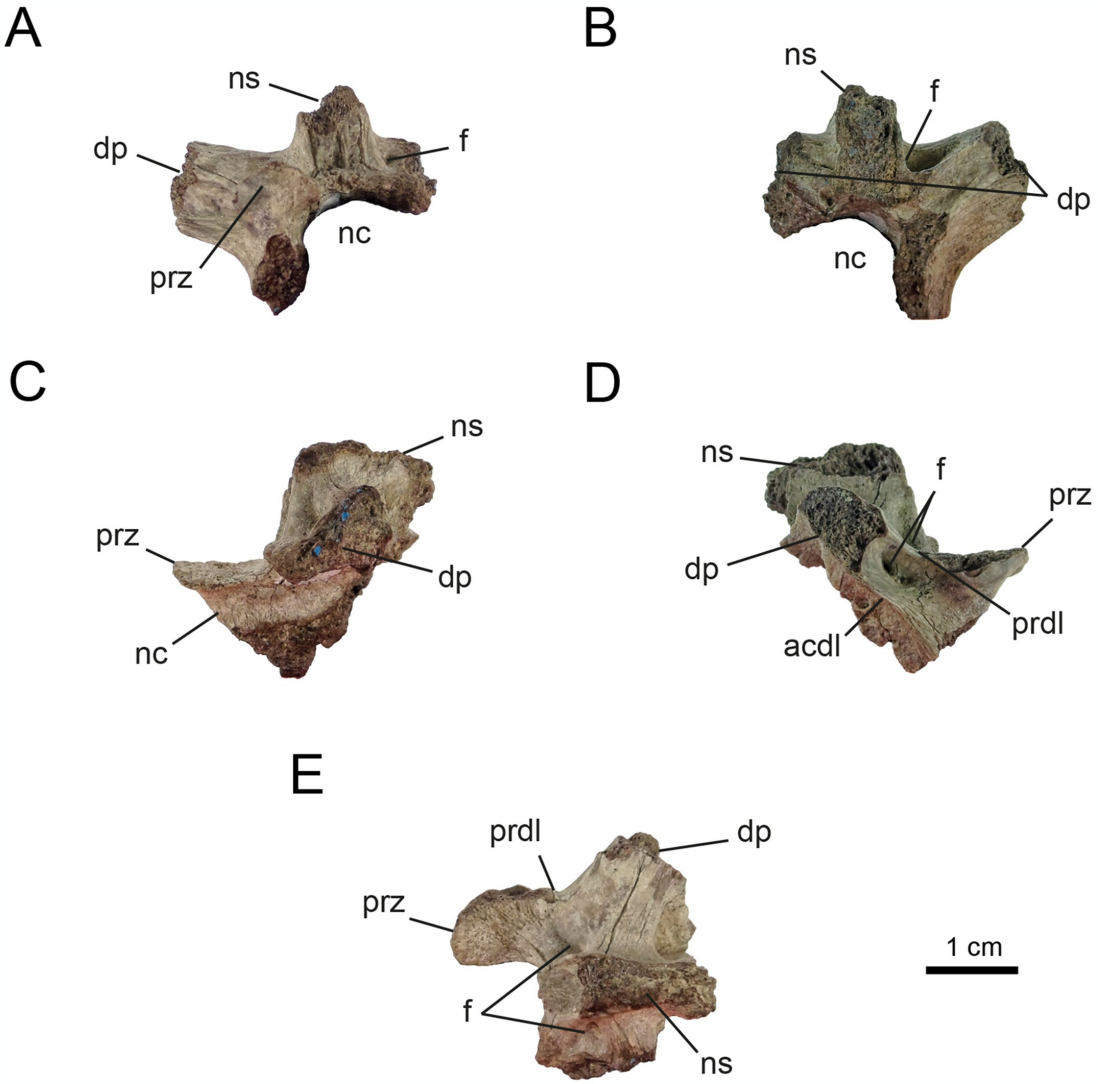
Middle-posterior cervical vertebrae of MPCN-PV 738.26. A, cranial view; B, caudal view; C, left lateral view; D, right lateral view; E, dorsal view. Abbreviations: acdl; anterior centrodiapophyseal lamina; prdl, prezygodipophyseal lamina; prz, prezygapophysis; dp, diapophysis; ns, neural spine; nc, neural channel; f, fossa. Scale bar equal to 1 cm.

**Fig 6:**
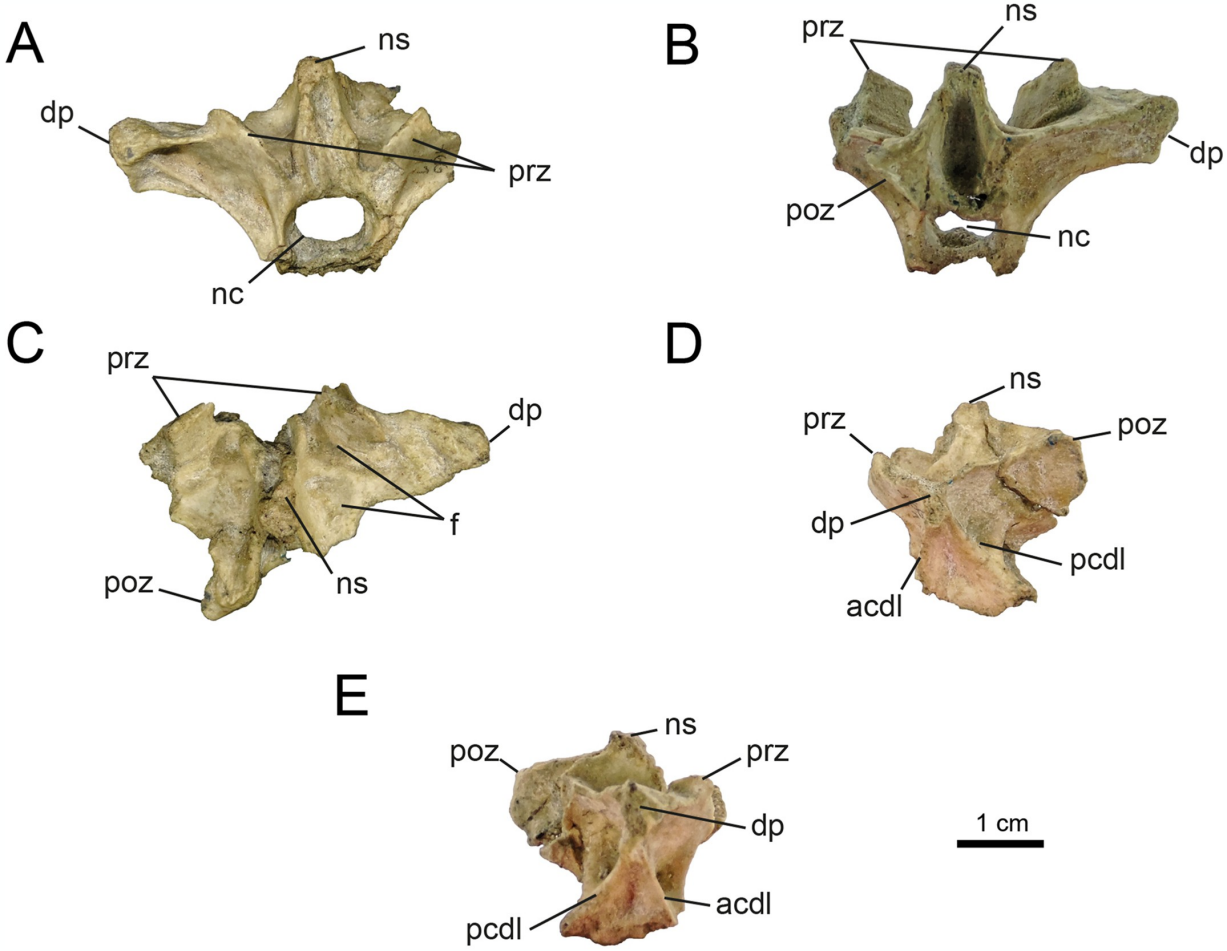
Middle-posterior cervical vertebrae of MPCN-PV 738.27. A, cranial view; B, caudal view; C, dorsal view; D, left lateral view; E, right lateral view. Abbreviations: acdl, anterior centrodiapophyseal lamina; pcdl, posterior centrodiapophyseal lamina; prz, prezygapophysis; poz, postzygapophysis; dp, diapophysis; ns, neural spine; nc, neural channel; f, fossa. Scale bar equal to 1 cm.

*4*.*2*.*1*.*1*. *Cervical vertebrae* (Figs 2–6). The new material includes six neural arches, two of them partially complete (MPCN-PV 738.26 and 738.27). Element MPCN-PV 738.26 lacks both postzygapophyses, the left prezygapophysis, a large portion of both transverse processes, most of the neural spine, and the base of the neural arch. As for MPCN-PV 738.27, this element lacks the left transverse process, a portion of the left prezygapophysis, the right postzygapophysis, and a small anterior portion of the neural spine. Of the remaining four neural arches, two of them are the most complete, preserving the lateral half of the neural arch (MPCN-PV738.28 is significantly larger than MPCN-PV 738.47). In this regard, MPCN-PV 738.28 lacks the left lateral half, right postzygapophysis and part of the right transverse process, while MPCN-PV 738.47 lacks the right lateral half. MPCN-PV 738.43 and 738.52 are neural arch fragments that do not provide additional information given their poor state of preservation. There is also a fragment of a centrum (MPCN-PV 738.32), a prezygapophysis (MPCN-PV 738.51) and three fragments of postzygapophyses (MPCN-PV 738.48, 738.50 and 738.55).

The neural arch of MPCN-PV 738.47 probably represents an anterior cervical vertebra on the basis of its general morphology, *i*.*e*., based on its craniocaudal length, dorsoventral height, shape, and the arrangement of the pre- and postzygapophyses (Fig 2). Regarding the mid-posterior cervical vertebrae, they are probably represented by elements MPCN-PV 738.26, 738.28, 738.51, 738.48, 738.50 and 738.55. As for the MPCN-PV 738.27 element, it represents an element that corresponds to the posterior series based on its general morphology.

MPCN-PV 738.47 is long, narrow, and low. The prezygapophyses are craniocaudally long and mediolaterally narrow, with their articular surfaces craniomedially oriented, as in other alvarezsaurids [1, 2, 6, 11, 12, 14, 16, 19, 21, 28, 36, 69, 74, 75]. This condition differs from the dorsally oriented, convex articular surfaces present in most coelurosaurs [76, 77]. Although the postzygapophysis is not completely preserved, its general morphology would indicate that the articular surfaces are oriented lateroventrally. Although the neural spine is not preserved, the basis of the spinoprezygapophyseal (sprl) and spinopostzygapophyseal (spol) laminae would indicate that the neural spine is short anteroposteriorly and probably located in the centre of the neural arch, as in other alvarezsaurians (e.g., *Alvarezsaurus*, *Shuvuuia*, *Haplocheirus*, *Aorun*, *Bannykus* and *Xiyunykus*; [1, 2, 6, 28, 74]). In lateral view, the postzygapophysis is elevated slightly dorsally relative to the prezygapophysis. All these characteristics could give an idea about its position within the cervical series, tentatively referring it to postions 3–5 of the series. The neural canal is tall compared to the neural arch, which is also observed in early diverging alvarezsaurians (e.g., *Aorun* and *Haplocheirus*; [1, 2]). Furthermore, in cranial view, the prezygapophysis develops above the level of the neural canal, while the postzygapophysis develops below that level (Fig 2). The systems of laminae and pits are well developed, unlike later-diverging alvarezsaurids [74]. The postzygodiapophysial lamina (podl) extends laterally and from the base of the postzygapophysis, linking it to the diapophysis (Fig 2). Ventral to this lamina is the centrodiapophyseal lamina (cdl), which posteriorly delimits the prezygapophyseal centrodiapophyseal fossa (prcdf), and anteriorly the small postzygapophyseal-centrodiapophyseal fossa (pocdf). The prezygapophyseal-centrodiapophyseal fossa is strongly excavated caudomedially, being delimited anteriorly by the prezygoparapophyseal lamina (prpl), posteriorly by the centrodiapophyseal lamina, and dorsally by the prezygodiapophyseal lamina (prdl). Regarding the postzygapophyseal-centrodiapophyseal fossa, it is delimited anteriorly by the centrodiapophyseal lamina, and posterodorsally by the postzygodiapophyseal lamina. The pocdf is strongly excavated craniomedially, being divided into two fossae by a small, dorsoventrally oriented underdeveloped lamina.

MPCN-PV 738.32 is a fragment of a cervical centrum, missing its dorsal half. It is opisthocoelous, a condition common among Alvarezsauridae, but unknown among early diverging alvarezsaurians such as *Haplocheirus* [1]. The anterior and posterior articular surfaces of the centrum are craniodorsally and caudoventrally inclined, respectively, which would indicate that it is an anterior element of the cervical series (Fig 3). This feature is typical of anterior cervical centra of theropods [78, 79]. The ventral surface of the cervical centrum is concave, and bears a pronounced, craniocaudally extending, mediolaterally broad groove (Fig 3), which is flanked by two small lateral ridges. On the anterior end of the ventral surface of the centrum, two laterally positioned bumps form the carotid processes (Fig 3). They are separated medially by the groove described above. The centrum is strongly compressed transversely at its mid-length. The dorsal portion of the centrum reveals bilaterally symmetrical internal pneumatic chambers.

The middle-posterior cervical neural arches are craniocaudally elongated (Figs 4 and 5) when compared to the distal cervical vertebrae (Fig 6). In other alvarezsaurids such as *Alvarezsaurus*, *Mononykus*, *Shuvuuia*, *Ceratonykus*, *Haplocheirus* and *Linhenykus*, the middle-posterior cervicals (elements 5–7) are distinctly the longest elements of the neck [1, 14, 16, 19, 28, 74, 75]. In some dromaeosaurids, including *Deinonychus*, *Sinornithosaurus* and *Bambiraptor* and unenlagiines, the middle-posterior cervicals are longer than the posteriormost ones [47, 80–82], a feature also present more broadly in other paravians [83–86] including avialans [87], as well as in ornithomimosaurians [88, 89]. The pre- and postzygapophyses reduce progressively in size along the cervical sequence (see Figs 4–6 and S2 File: “S5 Fig”), as in other alvarezsaurians [1, 13, 28, 74, 90]. The posteriormost elements show zygapophyses that are as wide as they are long (Fig 6). Well-developed epipophyses are present on the dorsal surface of the postzygapophyses along the craniolateral margin (Figs 5 and 6 and S2 File: “S5 Fig”), such as mentioned for *Haplocheirus* [1]. A small caudoventrally oriented flange is developed along the craniomedial edge of the postzygapophyses (Fig 6 and S2 File: “S5 Fig”). This feature is not present in other alvarezsaurids, so it could represent an autapomorphy of a new species represented by this specimen. As mentioned by [32], the transverse processes, here considered as belonging to a middle cervical, are laterocaudally projected, while those belonging to a posterior cervical are dorsolaterally projected (Figs 5 and 6) as in *Haplocheirus* [1] and *Khulsanurus* [91]. The neural spine is craniocaudally short, robust and vertically oriented. The neural canals are as wide as high and subcircular in cross-section (Figs 5 and 6).

The infrazygapophyseal fossa, is developed on the posterior surface of the transverse processes of the middle-posterior cervicals. It becomes increasingly deeper and decreasing in size throughout the series (Figs 4–6 and S2 File: “S6 Fig”), as in other alvarezsaurids. Distally in the series, the posterior centrodiapophyseal lamina is posteroventrally projected, while the anterior centrodiapophyseal lamina is anteroventrally projected. Both join dorsally where they contact each other along the transverse process, at an increasingly acute angle (Figs 5 and 6). Along the series, the centrodiapophyseal fossa goes from being craniocaudally and slightly excavated to narrow craniocaudally and deeply excavated. A similar increase in excavation occurs with the anterior fossa (centrodiapophyseal-prezygapophyseal fossa), which is ventrally delimited by the anterior centrodiapophyseal lamina and dorsally by the prezygodiapophyseal lamina. The centrodiapophyseal-postzygapophyseal fossa, which is delimited ventrally by the posterior centrodiapophyseal lamina, and dorsally by the postzygodiapophyseal lamina, becomes wide and strongly excavated distally in the series. In addition, throughout the succession, the prezygodiapophyseal lamina is reduced, while the postzygodiapophyseal lamina is well-developed as result of the laterodorsal projection of the transverse process and the rise of the diapophyses. The spinodiapophyseal lamina is reduced throughout the series in such a way that it is almost vestigial (Figs 5 and 6).

Along the series, the prezygodiapophyseal lamina is shortened, while the postzygodiapophyseal lamina is well-developed as a result of laterodorsal projection of the transverse process and the ascent of the diapophysis (Figs 5 and 6).

In dorsal view, the transverse processes of the anterior neural arches have a strongly concave spinodiapophyseal fossa running along their entire length. Nevertheless, in the middle cervical vertebrae, a prominent spinodiapophyseal lamina separates two fossae, the anterior and posterior spinodiapophyseal fossa. In the posterior cervical neural arches, this lamina is reduced in such a way that it is almost vestigial in comparison with the middle section of the neck.

**Fig 7:**
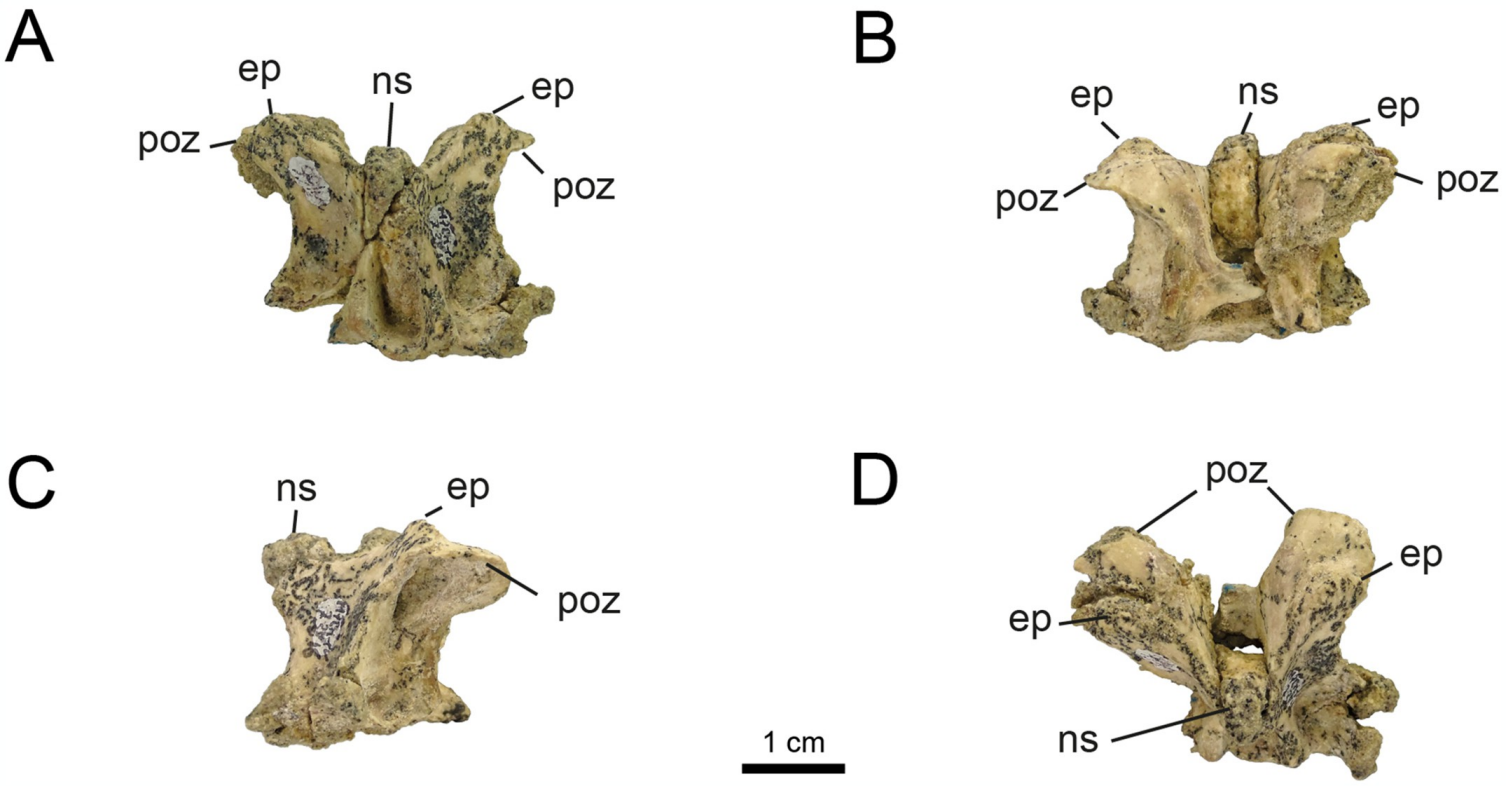
Cervico-dorsal vertebrae of MPCN-PV 738.16. A, cranial view; B, caudal view; C, left lateral view; D, right lateral view. Abbreviations: poz, postzygapophysis; ep, epipophysis; ns, neural spine. Scale bar equal to 1 cm.

**Fig 8:**
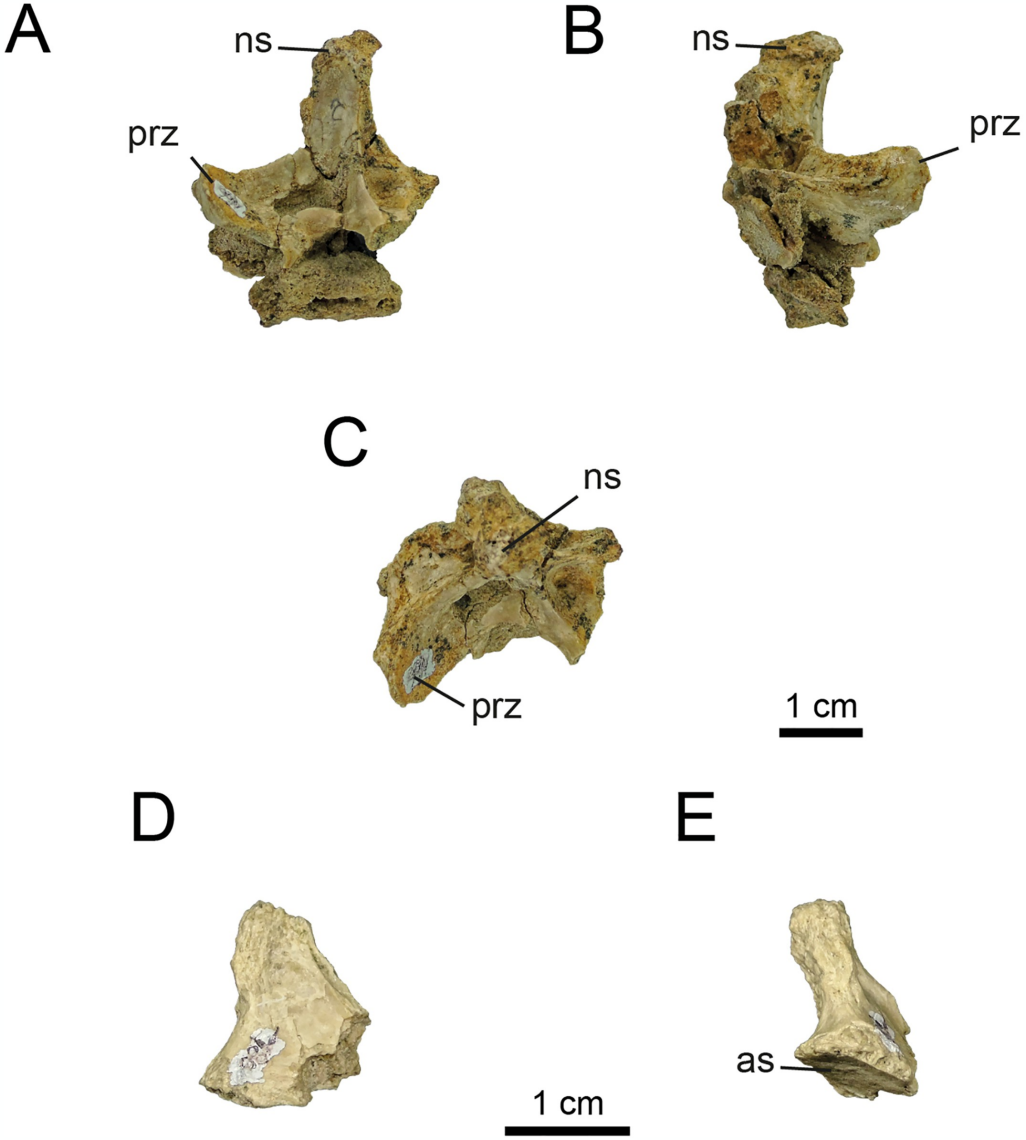
Cervico-dorsal vertebrae of MPCN-PV 738.15 and MPCN-PV 738.49. A, MPCN-PV 738.15 in cranial view; B, right lateral view; C, dorsal view; D, MPCN-PV 738.49 in lateral view; and E, caudal view. Abbreviations: prz, prezygapophysis; ns, neural spine; as, articular surface. Scale bar equal to 1 cm.

*4.2.1.2. Dorsal vertebrae* (Figs 7 and 8). The dorsal series is known from the elements MPCN-PV 738.15 and 738.16, which only preserve the partially complete neural arch. Both elements are considered here as belonging to the cervico-dorsal transition, based on the morphology of the elements known from this sector in Parvicursorinae (*e*.*g*., *Mononykus*, *Parvicursor*; [74, 75, 92]). The neural arches are very high compared to their craniocaudal length, with a ratio of 1.6. This ratio is similar to that observed in *Mononykus* and *Parvicursor* [75, 92]. The prezygapophyses are oval in dorsal view, wide and strongly dorsolaterally projected. The postzygapophyses are as long as wide, and ventromedially projected (Fig 7). The epipophyses are prominent and located laterodorsally above the postzygapophyses (Fig 7). The neural spine is dorsoventrally high and craniocaudally short (Figs 7 and 8).

An isolated postzygapophysis is part of the material (MPCN-PV 738.49; Fig 8). In dorsal view, this element is ellipsoidal and transversely compressed, a condition observed in *Patagonykus* and the holotype of *Bonapartenykus* [33, 36]. The postzygapophysis is transversely wide and craniocaudally shortened as in the holotype of *Bonapartenykus*. The posterior margin is notched, as in the holotype of *Bonapartenykus* [33]. In the original description of *Bonapartenykus*, [33] mentioned that the spinopostzygapophyseal lamina is wide and thick, and ends abruptly, which they interpret as an autapomorphy of the genus. This characteristic is present in element MPCN-PV 738.49 but also in *Patagonykus* (MCF-PVPH-37; pers. ob.), suggesting it is better interpreted as a synapomorphy of the Patagonykinae.

**Fig 9:**
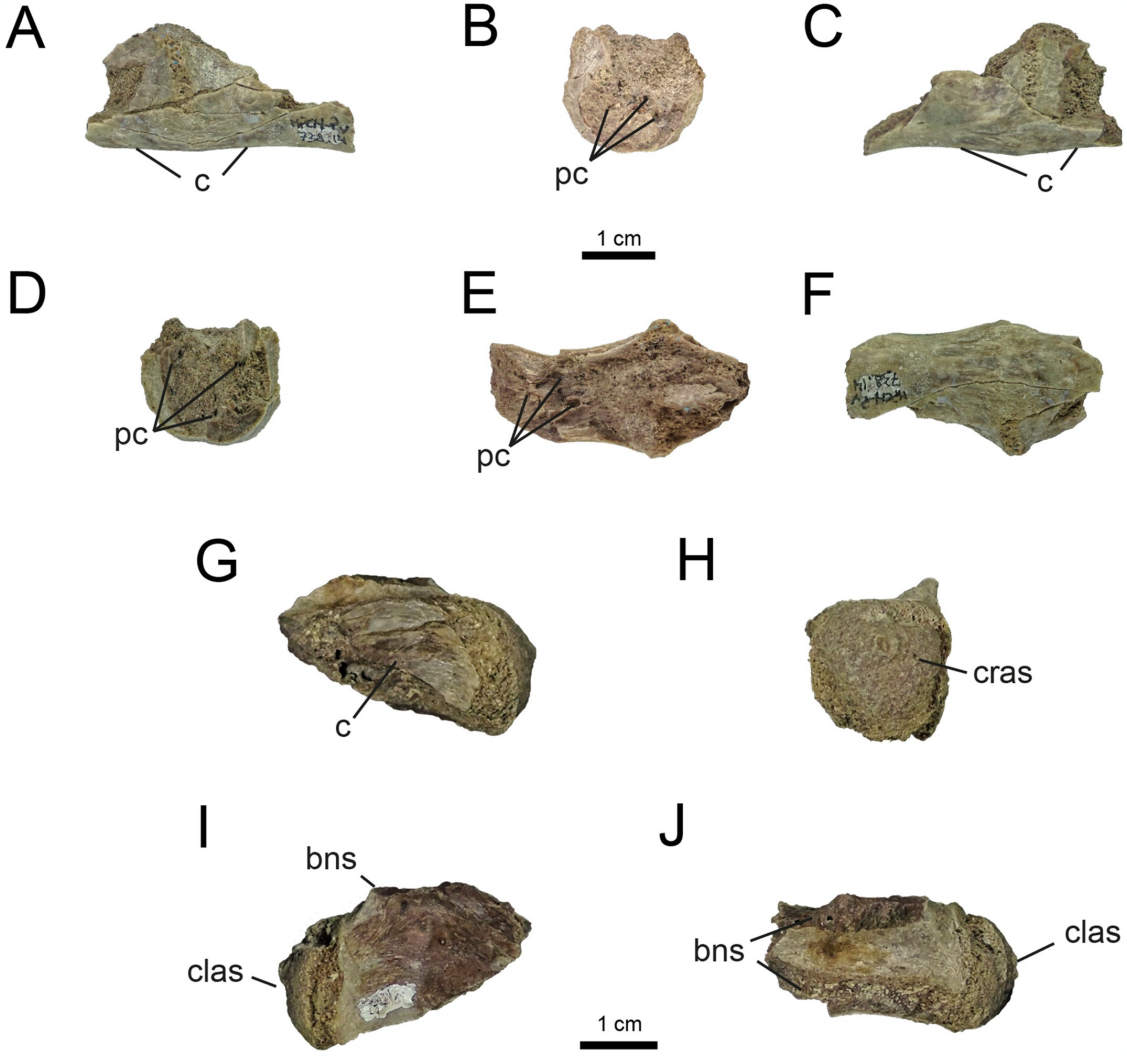
Sacral vertebrae of MPCN-PV 738. A, MPCN-PV 738.14 left lateral view; B, cranial view; C, right lateral view; D, caudal view; E, dorsal view; F, ventral view. G, MPCN-PV 738.31 in left lateral view; H, caudal view; I, right lateral view; J, dorsal view. Abbreviations: c, centrum; pc, pneumatic chambers; bns, base neural spine; cras, cranial articular surface; clas, caudal articular surface. Scale bar equal to 1 cm.

*4.2.1.3. Sacral vertebrae* (Fig 9). Three sacral vertebrae are preserved. These were briefly described by [32]. As those authors mentioned, the two anteriormost sacral vertebrae (MPCN-PV 738.14; Fig 9A–9F) are strongly fused to each other, and both are represented by a partially complete centrum. In *Shishugounykus inexpectus* (IVPP V23567), *Patagonykus puertai* (MCF-PVPH-37), and Parvicursorinae (*e*.*g*., *Mononykus*, *Shuvuuia*, *Parvicursor*, *Nemegtonykus*; [12, 74, 75, 92]) a strong fusion between the sacral vertebrae is observed. The second element, previously undescribed, is here interpreted as the last sacral vertebra (MPCN-PV 738.31; Fig 9G–9J), and is represented by a much more complete centrum.

Like in other alvarezsaurians, pneumatic fossae are absent in the sacral series. The ventral surfaces of the anteriormost sacral centra are convex, with a shallow longitudinal furrow (Fig 9F), as occurs in *Alvarezsaurus*, *Xixianykus*, and *Linhenykus* [18, 28, 69]. Although these elements lack much of their dorsal portions, they are laterally compressed, giving the whole bone a sub-cylindrical shape (Fig 9), as in other alvarezsaurians (*e*.*g*., *Shishugounykus*, *Patagonykus*, *Mononykus*, *Shuvuuia*, *Nemegtonykus*, *Parvicursor*; [3, 12, 36, 74, 92]. Based on this feature, we agree with Salgado et al. (2009)’s original allocation of these bones as the second and third sacral centra. We also interpret the more fragmentary centrum (the smallest) as the proximal-most element, based on knowledge of the sacral series in alvarezsaurians. As in *Shishugounykus* [3], MPCN-PV 738.14 bears small internal pneumatic chambers that are camellate and bilaterally arranged (Fig 9D and 9E).

The last sacral MPCN-PV 738.31 is transversely compressed, dorsoventrally high, and bears a ventral keel (Fig 9G–9J), as in some Patagonian and Asian forms (*e*.*g*., *Patagonykus* and *Mononykus*, [36, 75]). The distal articular surface is hemispherical (Fig 9G–9J), with the convex portion oriented posterodorsally, but not to the degree observed in *Patagonykus*.

Although this element is fragmentary, both lateral surfaces bear a short longitudinal lamina on the mid-dorsal portion of the centrum (Fig 9I). This lamina subdivides the centrum into two slightly concave dorsal and ventral halves. Ventral to the transverse process there is a small concavity. This morphology is also known in *Patagonykus*, so it probably represents a synapomorphy of Patagonykinae.

*4*.*2*.*1*.*4*. *Caudal vertebrae (Figs 13–19)*. In specimen MPCN-PV 738, eleven caudal elements from different sectors of the tail are known (*contra* six caudal elements originally described by [32], pp. 72). All centra are procoelous (previously mentioned for six caudals by [32]), with the anterior articular surfaces mediolaterally wider than the posterior surfaces, as in other alvarezsaurids.

**Fig 10:**
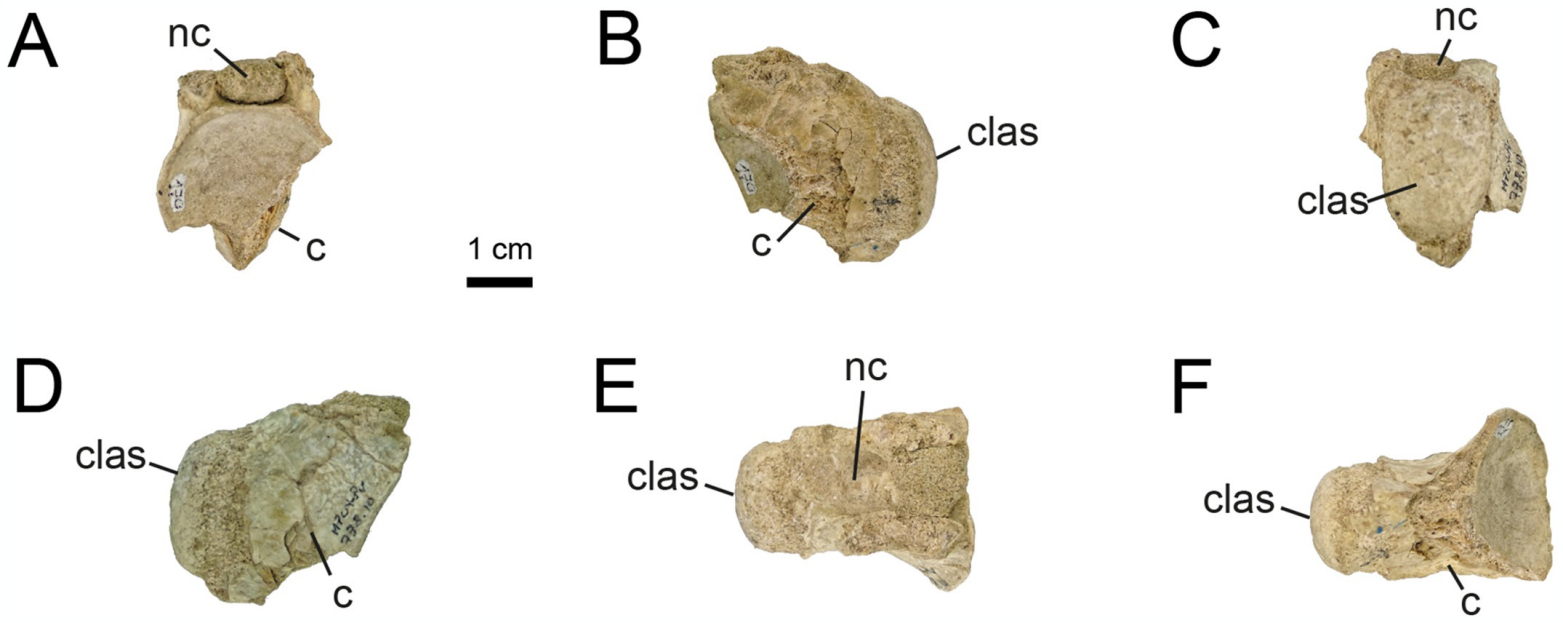
Proximal caudal vertebrae of MPCN-PV 738.10. A, cranial view; B, left lateral view; C, caudal view; D, right lateral view; E, dorsal view; F, ventral view. Abbreviations: clas, caudal articular surface; nc, neural channel; c, centrum. Scale bar equal to 1 cm.

**Fig 11:**
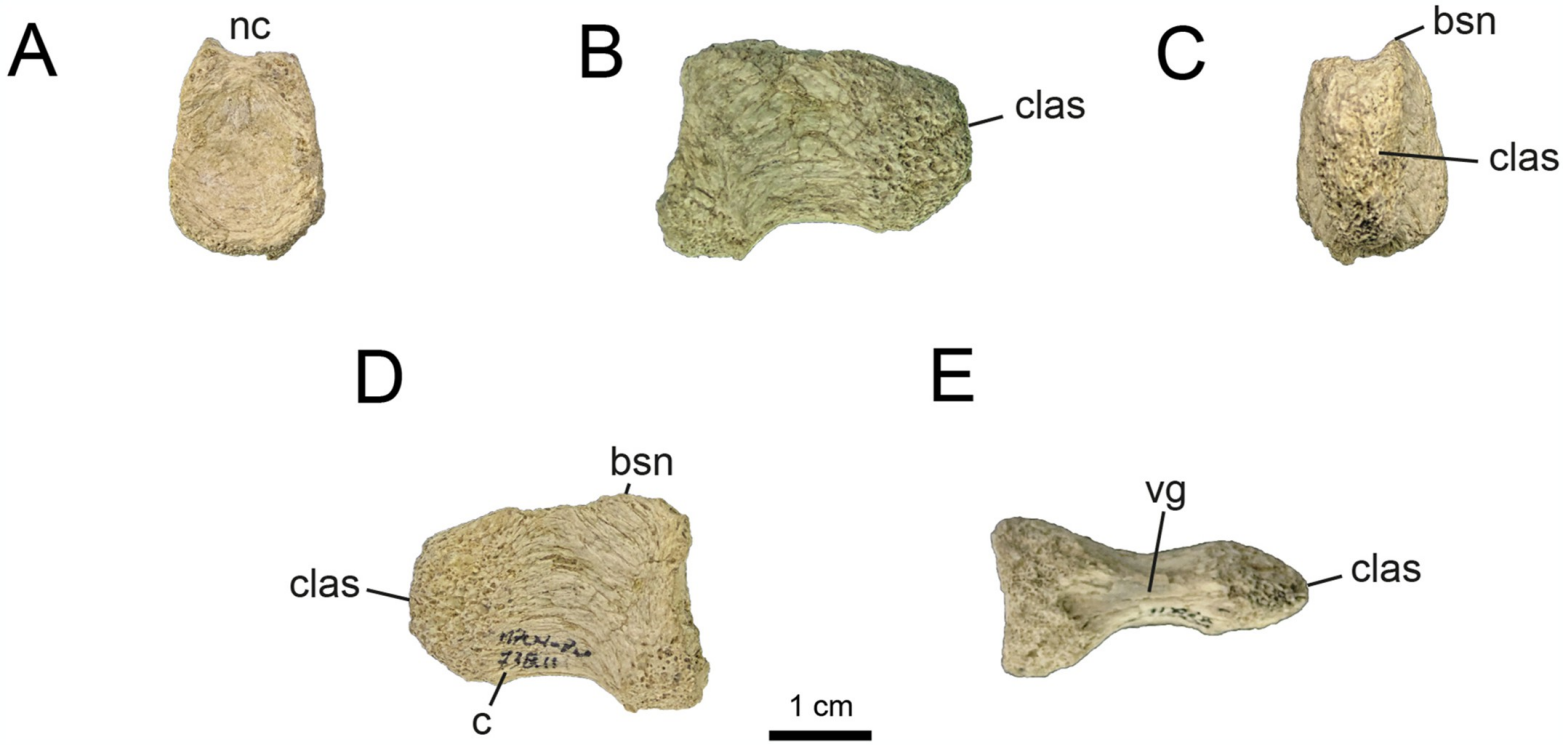
Proximal caudal vertebrae of MPCN-PV 738.11. A, cranial view; B, left lateral view; C, caudal view; D, right lateral view; E, ventral view. Abbreviations: bsn, base neural arch; clas, caudal articular surface; vg, ventral groove; c, centrum. Scale bar equal to 1 cm.

**Fig 12:**
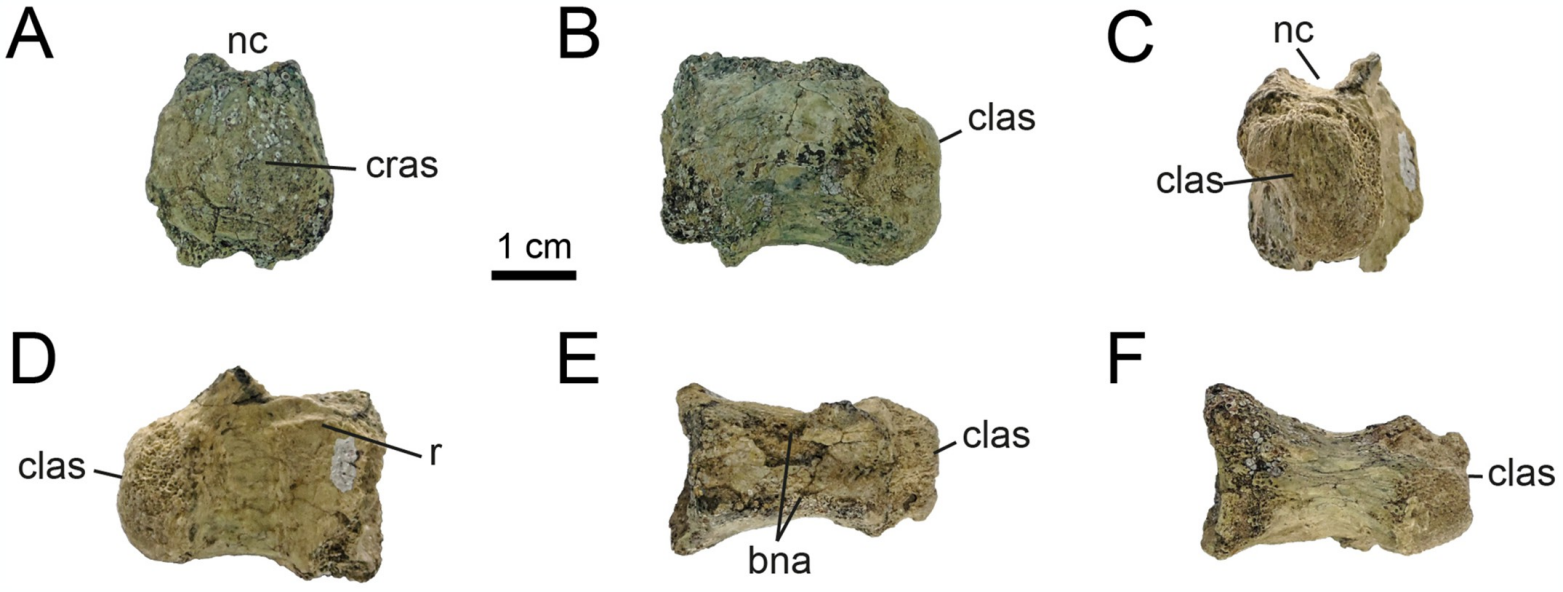
Proximal caudal vertebrae of MPCN-PV 738.29. A, cranial view; B, left lateral view; C, caudal view; D, right lateral view; E, dorsal view; F, ventral view. Abbreviations: cras, cranial articular surface; clas, caudal articular surface; bna, base neural arch; nc, neural channel; r, ridge. Scale bar equal to 1 cm.

Based on its general morphology (i.e., length/height, length/width, and height/width ratios) and prominent articular surfaces, MPCN-PV 738.10 is tentatively referred as the first caudal (Fig 10). The centra MPCN-PV 738.11 and 738.29 are eroded and slightly deformed. Based on their dimensions, they could be proximal caudals, tentatively allocated to positions 2–4 of the series. Like the proximal caudal vertebrae of other alvarezsaurids, these elements are characterised by having relatively high lateral surfaces that are slightly concave craniocaudally and convex dorsoventrally (Figs 10–12). Contra [32], the MPCN-PV 738.11 vertebral centrum shows signs of small subcircular depressions on both lateral surfaces (Fig 11).

While elements MPCN-PV 738.30, 738.37, 738.38 and 738.40 are fragmentary remains (sometimes only their articular surfaces are preserved; S2 File: “S7 Fig”), they can be identified as proximal caudals due to their strong lateral compression, and likely represent positions 5–8.

**Fig 13:**
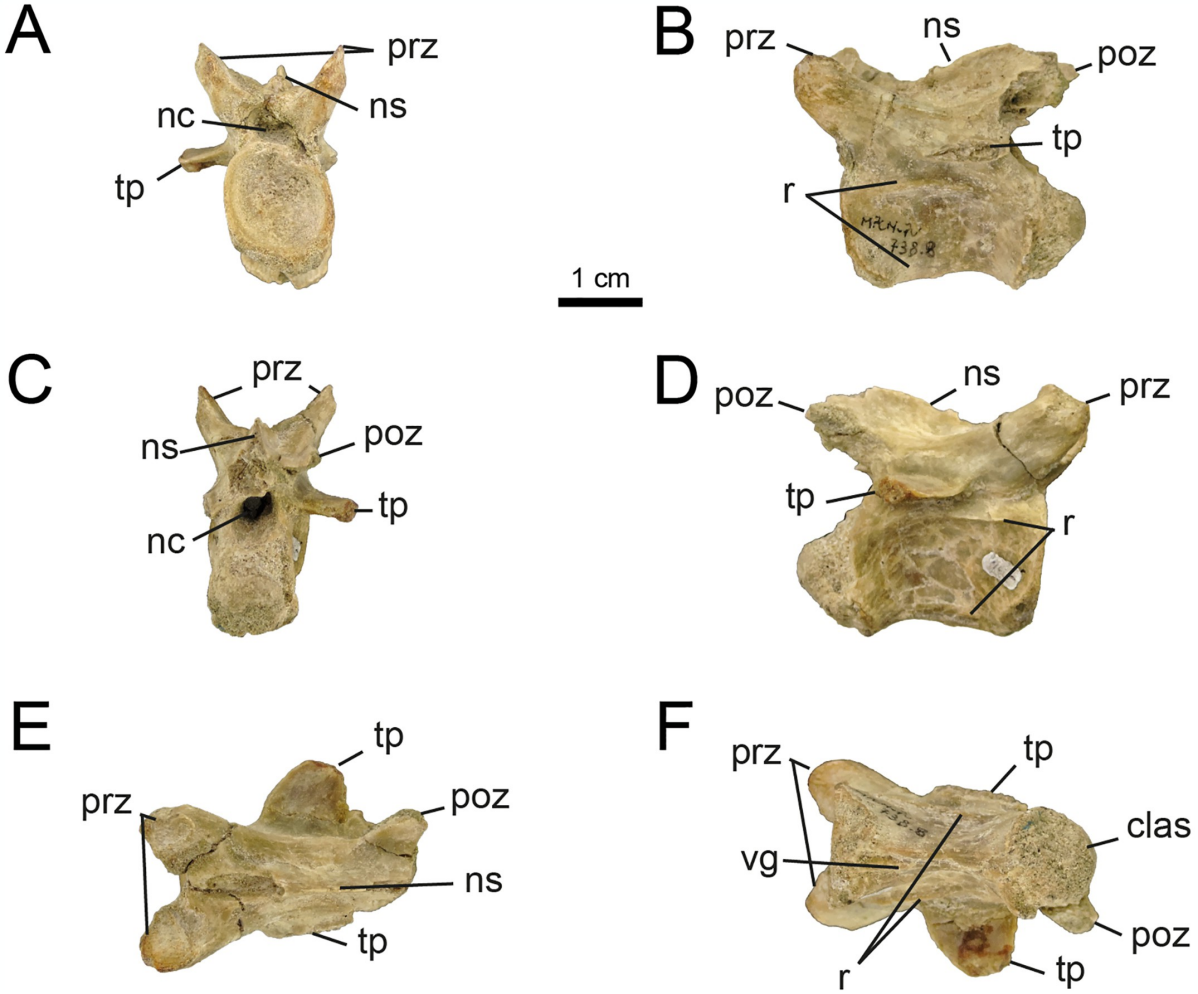
Proximal caudal vertebrae of MPCN-PV 738.8. A, cranial view; B, left lateral view; C, caudal view; D, right lateral view; E, dorsal view; F, ventral view. Abbreviations: clas, caudal articular surface; prz, prezygapophysis; poz, postzygapophysis; tp, transverse process; vg, ventral groove; r, ridge. Scale bar equal to 1 cm.

**Fig 14:**
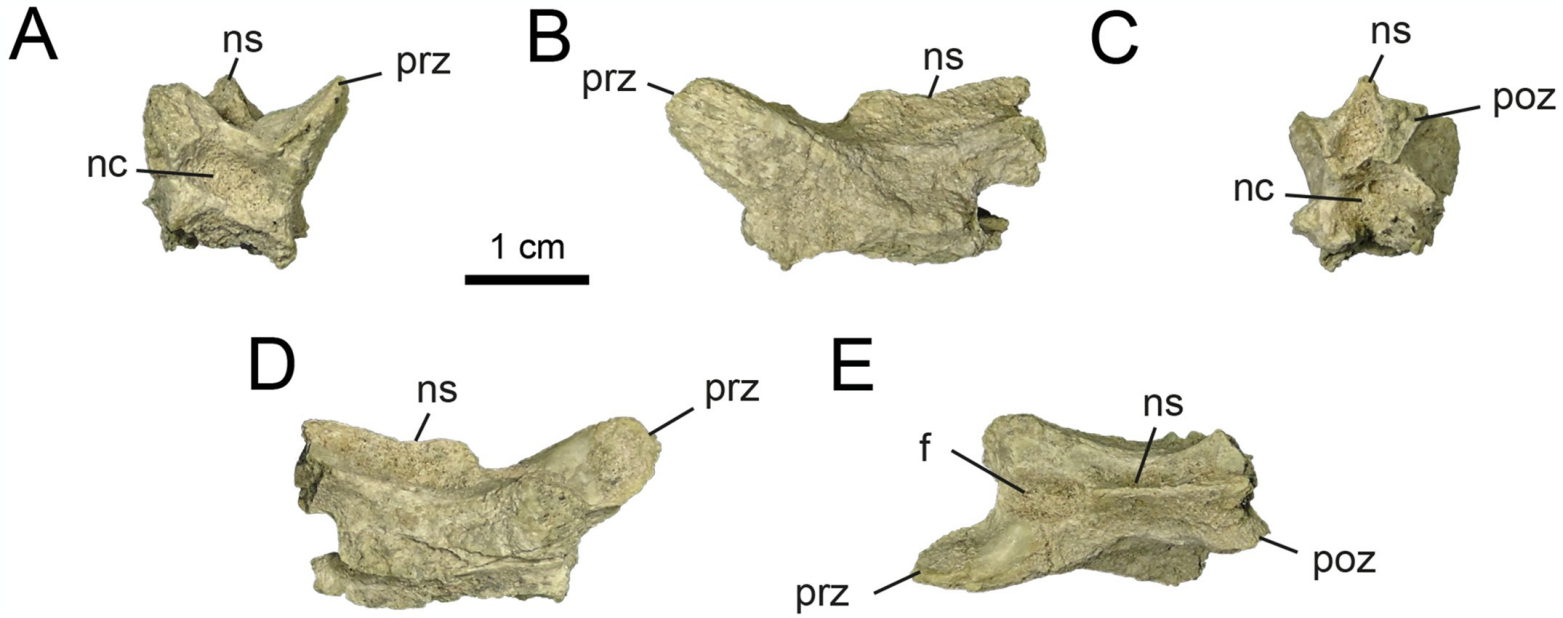
Proximal caudal vertebrae of MPCN-PV 738.58. A, cranial view; B, left lateral view; C, caudal view; D, right lateral view; E, dorsal view. Abbreviations: prz, prezygapophysis; ns, neural spine; poz, postzygapophysis; nc, neural channel; f, fossa. Scale bar equal to 1 cm.

The caudal vertebrae MPCN-PV 738.8 and 738.58 are the most complete axial elements and the only ones that provide abundant anatomical information. These elements correspond to positions 8 and 9 of the caudal series, based on the angle of projection of the transverse processes and the anterolateral and posterolateral orientation of pre- and postzygapophyses, respectively (Figs 13 and 14). The neural arch is longer than high, and the prezygapophyses extend cranially beyond the level of the centrum, whereas the postzygapophyses end at the same caudal level as the posterior articular surface (Figs 13 and 14). The neural spine is low, craniocaudally extended and posteriorly inclined, presumably at the level of the postzygapophyses, as occurs in specimens MPC 100/975, MPC-D 100/203 and IVPP V15988. The prezygapophyses are longer than wide (oval outline in dorsal view; Figs 13 and 14), with their major axes craniolaterally oriented, whereas the postzygapophyses are small and caudolaterally projected. The transverse processes are caudolaterally projected, being as long as wide, slightly inclined ventrally, and situated distally on the centra (Fig 13). As mentioned by [32], ventral to the transverse process there is a longitudinal flange that runs through the centrum (Fig 13). This character has not been mentioned in other alvarezsaurids, so it could represent an autapomorphy of *Bonapartenykus*. Ventrally, the centra show a sharp ventral edge bearing a longitudinal groove, as observed by [32].

MPCN-PV 738.9 is an isolated postzygapophysis tentatively referred to the seventh or eighth caudal vertebra based on the caudal morphology of specimens MPC-D 100/203 and IVPP V15988.

**Fig 15:**
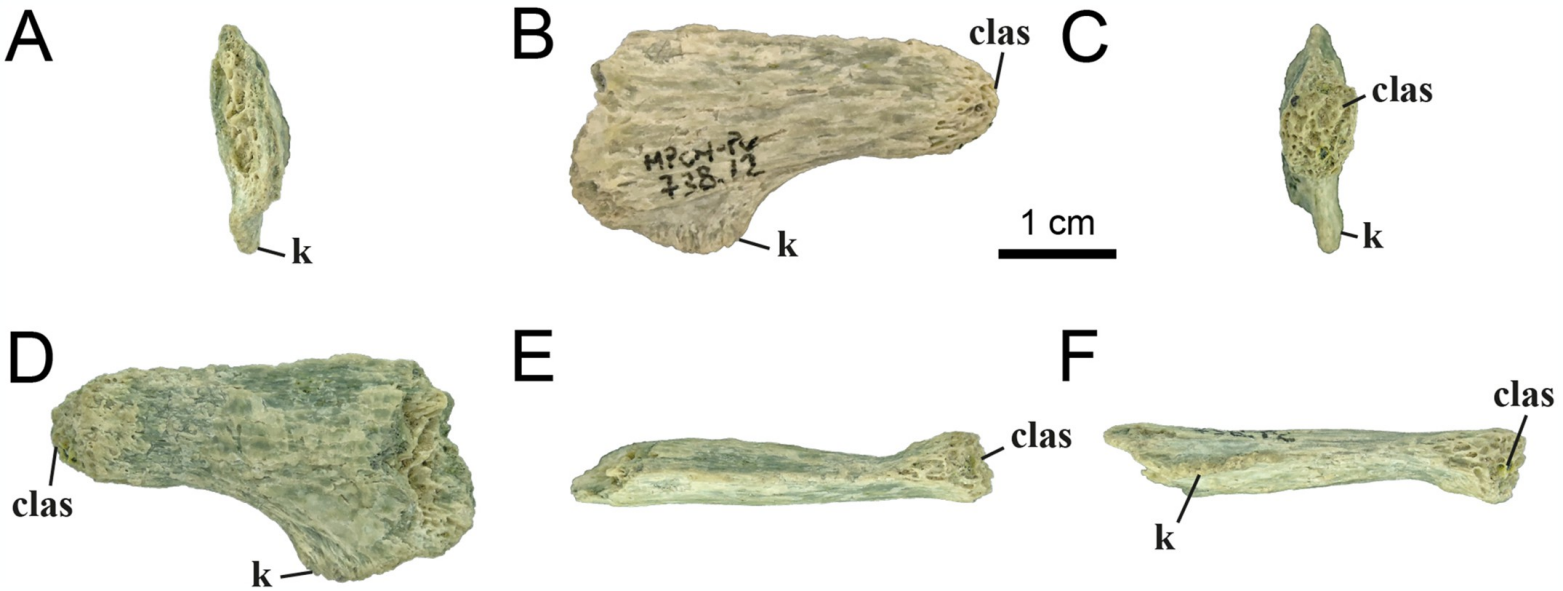
Distal caudal vertebrae of MPCN-PV 738.12. A, cranial view; B, left lateral view; C, caudal view; D, right lateral view; E, dorsal view; F, ventral view. Abbreviations: clas, caudal articular surface; k, keel. Scale bar equal to 1 cm.

The element MPCN-PV 738.12 is strongly compressed laterally, being six times longer than wide. Although it is poorly preserved, it is strongly procoelous, and it has a prominent ventral longitudinal keel (Fig 15). For these reasons, we identify it as a distal caudal vertebra, but we are unable to assign it to a specific position within the caudal series.

*4*.*2*.*1*.*5*. *Dorsal ribs*. A probable left dorsal rib (MPCN-PV 738.36; S2 File: “S8A–S8D Fig”) and a fragment of dorsal rib (MPCN-PV 738.35; S2 File: “S8E and S8F Fig”) are preserved. The capitulum is not preserved, but the tuberculum is very short with an elliptical and craniocaudally compressed articular surface. MPCN-PV 738.36 is proximally slender, and distally massive, where it adopts a kidney-shaped contour in cross-section.

*4*.*2*.*1*.*6*. *Haemal arches*. MPCN-PV 738.7 is interpreted as a proximal-middle haemal arch (S2 File: “S9 Fig”). It is, dorsoventrally elongate and robust. Dorsally it bifurcates in two rami. On the anterior surface of the ventral blade, there is a small and robust crest. On the posterior surface, there is a longitudinal groove. Dorsally the element is as wide as long, but distally is wider than long. It is compressed craniocaudally.

#### 4.2.2. Appendicular skeleton

**Fig 16:**
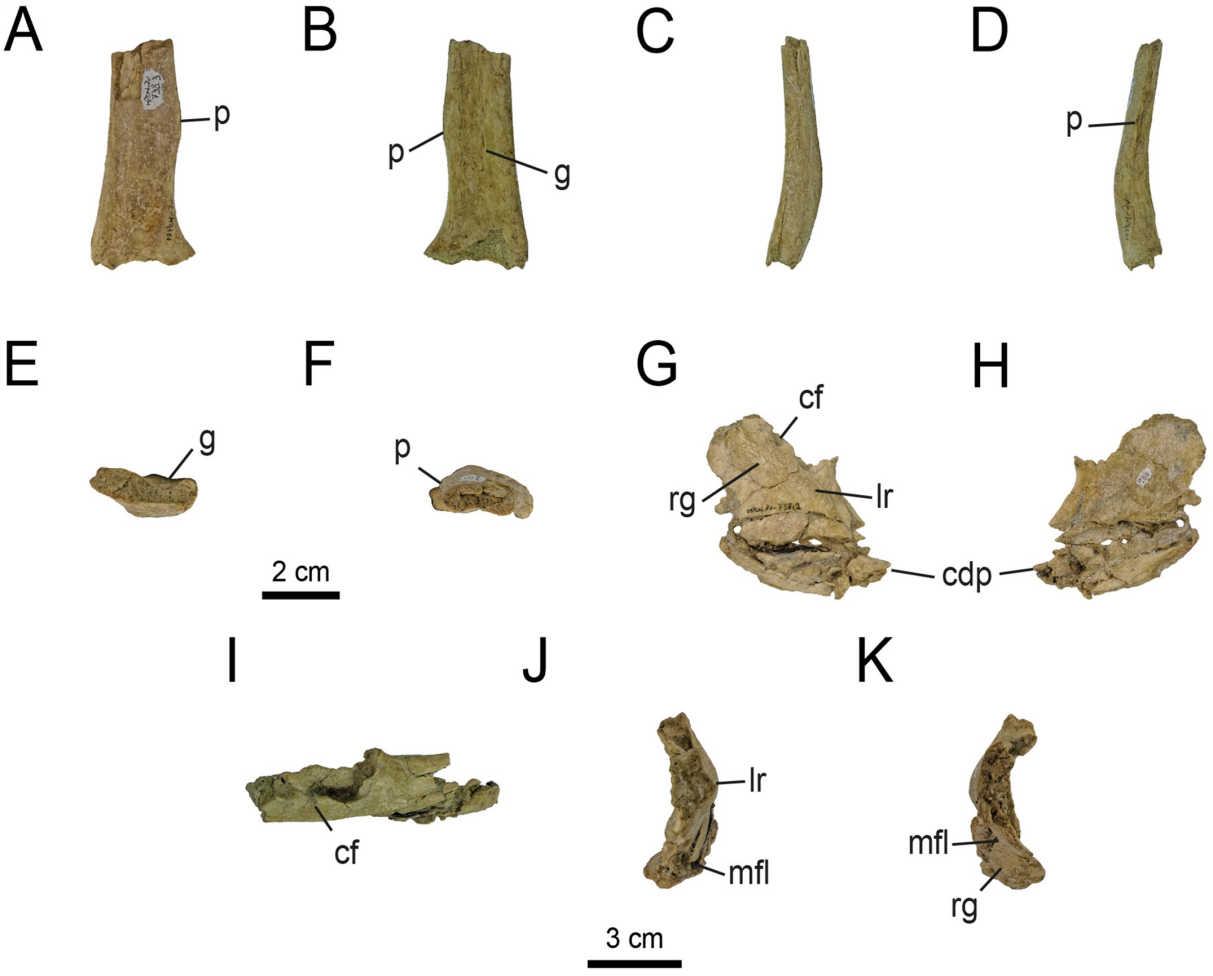
Left scapulocoracoid of MPCN-PV 738. A, MPCA 1290. A, MPCN-PV 738.3 in lateral view; B, medial view; C, cranial view; D, caudal view; E, distal view; F, proximal view; G, MPCN-PV 738.2 in lateral view; H, medial view; I, dorsal view; J, cranial view; K, caudal view. Scale bar equal to 3 cm.

*4*.*2*.*2*.*1*. *Pectoral girdle*. *4*.*2*.*2*.*1*.*1*. *Scapula and coracoid* (Fig 16): The left scapulocoracoid of specimen MPCN-PV 738 (comprising MPCN-PV 738.3 and 738.2) is well preserved despite being divided into two elements (Fig 16). These elements are proportionally smaller relative to the scapulocoracoids previously known for *Bonapartenykus*. Only the proximal half of the scapular blade is preserved. The coracoid is missing its proximalmost and distalmost portions. The scapular blade is medially and craniodorsally deflected, unlike in *Alvarezsaurus calvoi* (MUCPv-54) and *Xiyunykus pengi* (IVPP V22783), where the scapular blade deflects caudodorsally. As in early diverging (e.g. *Haplocheirus sollers* and *Bannykus wulatensis*; [1, 6]) and later-diverging (e.g. *Mononykus olecranus* and *Shuvuuia deserti*; [74]) alvarezsaurians, the scapulocoracoid is characterised by a straight scapular blade. Although the distal end of the scapular blade is not preserved, it likely would be compressed, since the dorsal and ventral margins tend to converge distally. This condition is comparable with *Alvarezsaurus*, the holotype of *Bonapartenykus* and to a lesser extent with *Xiyunykus*, but contrary to the condition observed in most alvarezsaurids, where the distal portion expands craniocaudally and ends abruptly [1, 6, 74, 75].

The fragment of left scapular blade bears a convex lateral surface and its medial surface is longitudinally crossed by a shallow groove. Such a groove divides the medial surface into dorsal and ventral sections, whose surfaces are convex dorsoventrally (kidney-shaped in cross-section), as observed by [32]. This character, originally described in element MPCN-PV 738.3, is also present in the holotype of *Bonapartenykus* (MPCA 1290). The lateroventral edge of the proximal end is transversely thickened as originally observed by [32], and occurs in the type material of *Bonapartenykus* (MPCA 1290). In addition, a small and sharp, gently convex process is described, which develops on the ventral edge (Fig 16D). This character is also observed in the holotypes of *Bonapartenykus* and *Mononykus* [75]. On the ventral margin between this process and the glenoid cavity, there is a well-developed and craniodistally extended notch, a condition also observed in *Mononykus* (op. cit.) and the holotype of *Bonapartenykus*.

In lateral view, the coracoid has a shallow ventral blade and elongate posteroventral process (Fig 16G and 16H), as in *Ceratonykus*, the holotypes of *Bonapartenykus*, *Shuvuuia*, *Mononykus*, *Patagonykus*, *Alnashetri* (MPCA 377), *Haplocheirus*, *Xiyunykus* and *Fukuivenator* [1, 33, 35, 36, 47, 74, 75, 93] and probably in *Alvarezsaurus*. The ventral half of the blade is strongly flexed medially, as observed in *Fukuivenator*, *Ceratonykus*, *Patagonykus* and the holotype of *Bonapartenykus* [16, 33, 36, 93]. In this last taxon, the angle of flexion is much greater than in *Fukuivenator*, *Ceratonykus*, *Patagonykus* and MPCN-PV 738.2. Along the lateral margin of the blade of MPCN-PV 738.2 there is a longitudinal crest (Fig 16G), which is less pronounced than in the holotype of *Bonapartenykus*, and that could be an ontogenetic effect. This ridge is projected at a lower angle than in the *Bonapartenykus* holotype and divides the coracoids in two zones. The ventral surface of the coracoid of MPCN-PV 738 is characterised by being convex throughout its craniocaudal length. The grooves and striations that develop on the ventral surface of the coracoid of MPCN-PV 738 are limited to the longitudinal crest, contrary to the condition observed in the holotype of *Bonapartenykus*, whose grooves and striations are developed not only on the ventral surface of the coracoid, but above the longitudinal ridge over the caudalmost zone of the dorsal surface of the coracoid. The cranial margin of the coracoid is mediolaterally thick, as occurs in early diverging alvarezsaurians (*Haplocheirus* and *Xiyunykus*; [1, 6]) and Patagonian forms [28, 33, 36], and cranially truncated, as in Coelurosauria more generally [46, 76, 94, 95]. Caudally, the coracoid is dorsoventrally low, and ends in an acute projection (Fig 16G), a condition also observed in the holotypes of *Bonapartenykus* and *Patagonykus*.

This element lacks a biceps tubercle (= bicipital tubercle or coracoid tubercle; [1, 33]), as is the case in Patagonian alvarezsaurids, except *Alnashetri* [35], and later-diverging Asian forms except *Mononykus* [75]. This structure is present in early diverging alvarezsaurians, such as *Bannykus*, *Haplocheirus* and *Xiyunykus* [1, 6]. The coracoid foramen is situated cranially to the glenoid fossa (Fig 16G). The coracoid foramen is elliptical in lateral view as in the holotype of *Bonapartenykus*. In MPCN-PV 738 as well as in the holotype of *Bonapartenykus*, there are several ridges that are developed caudal to the glenoid fossa, which could correspond to the insertion of the *M*. *coracobrachialis brevis* (Fig 16G).

**Fig 17:**
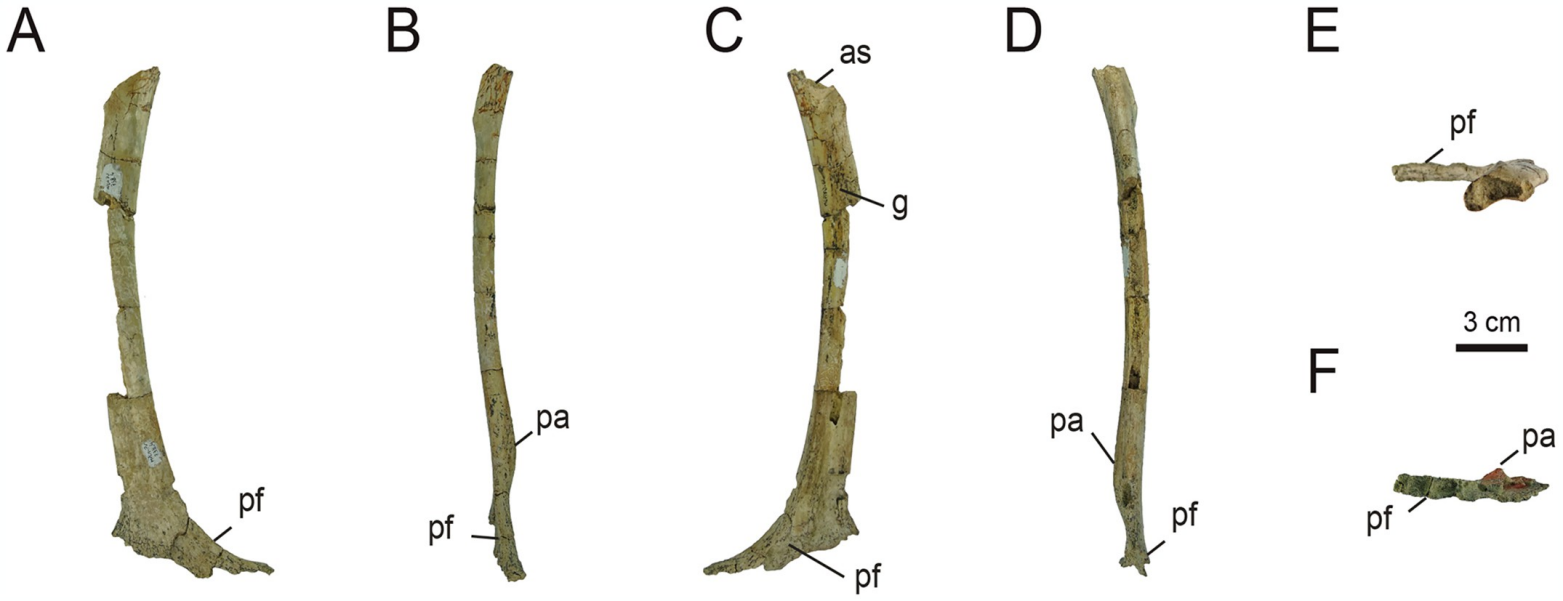
Left pubis of MPCN-PV 738.4. A, lateral view; B, caudal view; C, medial view; D, cranial view; E, proximal view; F, distal view. Abbreviations: g, groove; as, acetabular surface; pf, pubic foot; pa, pubic apron. Scale bar equal to 3 cm.

*4*.*2*.*2*.*2*. *Pelvic girdle*. *4*.*2*.*2*.*2*.*1*. *Pubis* (Fig 17): Specimen MPCN-PV 738 preserves a nearly complete left pubis, lacking only a mid-posterior portion on the caudal margin (Fig 17). Originally, [32] described a proximal fragment of a left pubis; however, it was possible to reconstruct a larger part of this element. The shaft of the pubis in general is long and slender, a condition similar to that observed in *Patagonykus* [36] and the holotype of *Bonapartenykus* [33]. This condition contrasts with the Parvicursorinae, where the distal shaft is rod-like [8, 18, 24, 74, 75]. In general, the pubic shaft in MPCN-PV 738 is craniocaudally bowed, with its anterior margin convex and its posterior margin concave (Fig 17). This condition differs subtly from that observed in *Patagonykus* ([36], fig. 22), where the shaft is slightly straight cranially and bowed caudally as occurs in MPCN-PV 738. However, in the holotype of *Bonapartenykus*, the shaft is cranially subtly curved, with a slightly straight to slightly convex anterior margin, and a concave posterior margin, while distally the shaft is straight ([33], Fig 5). In cranial or caudal view, the shaft is slightly sigmoidal (Fig 17), as occurs in Patagonykinae, but straighter with respect to the condition observed in the holotypes of *Bonapartenykus* and *Patagonykus*. Although the proximal end is not totally preserved, the morphology of this element could indicate a posteroventral orientation (= mesopubic condition *sensu* [96]) based on the base of iliac and ischiac pedicels that are preserved.

In cross section, MPCN-PV 738.4 is oval as in *Patagonykus* [36] and the holotype of *Bonapartenykus* [33], unlike Asian forms where it is subcircular. However, in the holotype of *Bonapartenykus* it maintains its proportions (of length and width) in cross-section along of all of the pubic shaft, unlike *Patagonykus* and MPCN-PV 738.4, whose proximal-medial sector is strongly compressed lateromedially.

The medial surface of the shaft is smooth. It bears a longitudinal furrow that deepens distally (Fig 17) and divides the medial surface into cranial and caudal sections. The surfaces of these two sections are convex craniocaudally, lending the shaft a kidney shape in cross-section. This condition is absent in the holotype of *Bonapartenykus*, and is subtly perceptible in *Patagonykus*. The lateral surface is also smooth, but slightly convex (Fig 17). Above the proximal end of the caudal section, there is a prominent tubercle that develops obliquely, as occurs in *Patagonykus*, although in MPCN-PV 738.4 it is incipient. As mentioned by [32], this structure possibly represents the insertion of the *M*. *ambiens* [32, 97].

Distally, on its medial surface, there is a small ridge on the cranial margin, which represents a reduced pubic apron (Fig 17), a feature that is also observed in *Patagonykus* and the holotype of *Bonapartenykus*, but is absent in most later-diverging species [18, 33, 36]. Below this structure, there is a prominent, craniocaudally expanded pubic foot. Although not completely preserved, its lateral surface is slightly concave with fine grooves and distal rugosities, as observed in *Patagonykus* [36].

**Fig 18:**
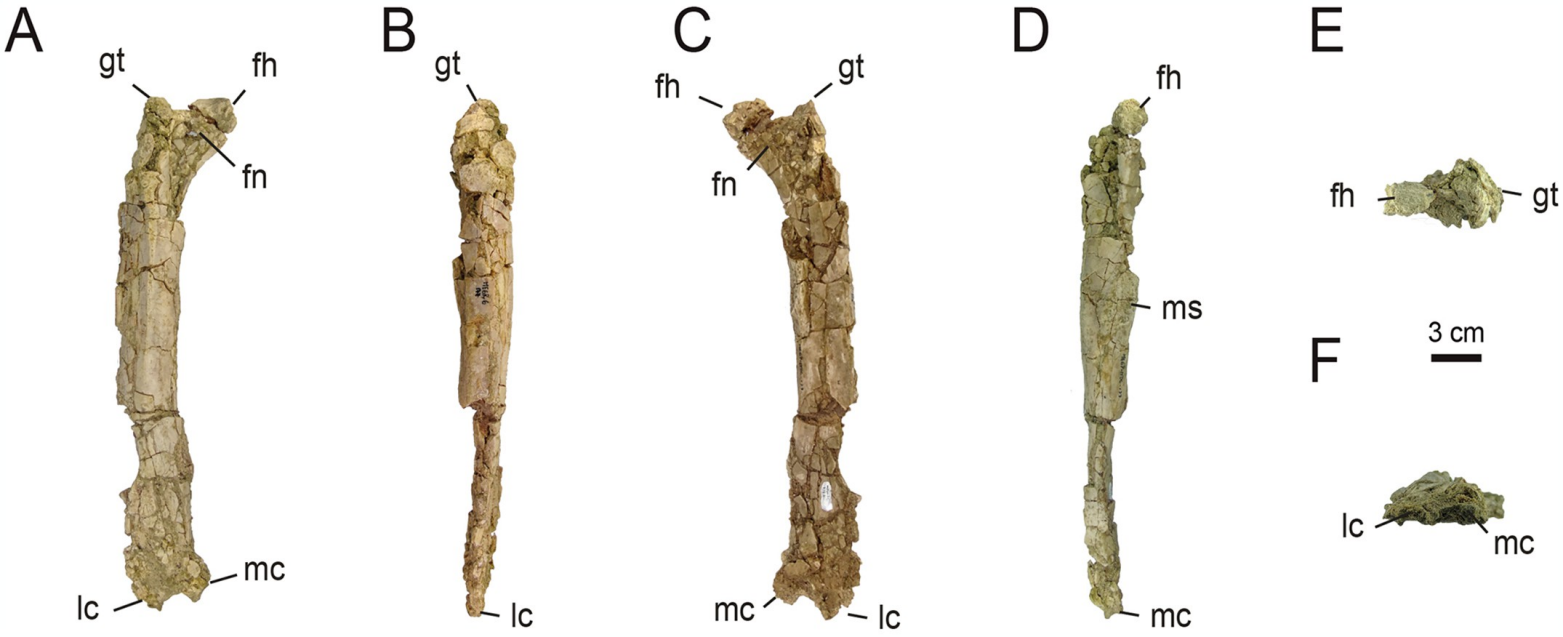
Right femur of MPCN-PV 738.1. A, cranial view; B, lateral view; C, caudal view; D, medial view; E, proximal view; F, distal view. Abbreviations: gt, greater trochanter; fn, femoral neck; fh, femoral head; lc, lateral condyle; mc, medial condyle; ms, muscle scar. Scale bar equal to 3 cm.

*4*.*2*.*2*.*3*. *Hindlimb*. *4*.*2*.*2*.*3*.*1*. *Femur* (Fig 18): Right femur MPCN-PV 738.1 (catalogued previously as MEPyG-177) is almost complete, but badly preserved. It experienced craniocaudal crushing, and lacks most of its distal end. [58] assigned this bone to Iguanodontia, but [33] considered that it is assignable to Alvarezsauridae because it lacks of a pendant fourth trochanter, and bears a basitrochanteric fossa [33, 98]. We accept this last assignation.

In anterior or posterior view, the femoral shaft is straight (Fig 18), as occurs in *Xiyunykus* [6], the holotype of *Bonapartenykus* [33], and probably in many Patagonian forms [28, 31, 36]. This condition contrasts with that observed in later-diverging alvarezsaurids [8, 18, 75], and early diverging forms such as *Alnashetri*, *Haplocheirus* and *Tugulusaurus*, where the femoral shaft is slightly bowed proximally and straight to slightly curved distally [1, 5, 34]. Nevertheless, this condition in MPCN-PV 738.1 could be an artifact of craniocaudal collapse. In lateral or medial view, it is slightly sigmoidal (Fig 18), with the shaft being laterally convex in the proximal part and medially convex distally, as occurs in Coelurosauria [1, 6, 8, 75, 84, 95, 99–108].

In general, although this element is highly crushed, the proportions of the preserved parts indicate that the femur of MPCN-PV 738 was long and robust, as in early diverging alvarezsaurians and probably in *Patagonykus* [1, 6, 36], with the exception of the holotype of *Bonapartenykus*, since its proportions indicate a short and robust element [33]. This differs in Parvicursorinae, where the femur is elongated and slender.

The femoral head is robust, medially and slightly anteriorly projected (Fig 18), as in alvarezsaurids. The condition is not discernible in *Bonapartenykus*, although it is probable that this condition was present in this last genus, based on the preserved portion at the base of its femoral head. The femoral head is craniocaudally narrow, as occurs in *Anomalipes* [109], unlike other taxa where the femoral head is subcircular in cross-section [5, 6, 31, 47, 69, 75, 92]. Posterior to the femoral head, there is a wide, prominent and oblique groove (Fig 18), which corresponds to the passage of the *ligamentum capitis femoris* [62]. This groove is present in *Zuolong salleei* [110], although is not seen in other alvarezsaurids.

In anterior view, the femoral head and greater trochanter are separated by a cleft (Fig 18), a condition observable in *Patagonykus* [36], *Achillesaurus* [31], Asian later-diverging forms [69, 75, 92], and probably in *Alvarezsaurus* and in the holotype of *Bonapartenykus*. This condition contrasts with that observed in *Xiyunykus* and *Tugulusaurus*, where the femoral head is confluent with the greater trochanter. As mentioned by [58], in MPCN-PV 738 the greater trochanter is convex dorsally, flat laterally and the upper edge is at the same level as the dorsal surface of the femoral head, and craniocaudally expanded (Fig 18). However, there is no evidence of, at least, a partial fusion between the greater, lesser and posterior trochanters to form a trochanteric ridge. The posterior trochanter is prominent, proximally placed, and flat transversely, and probably its dorsal projection would have been lower than the greater trochanter. In postero-medial view, there is a rugose area on the shaft bearing numerous small tubercles, which is the scar for the insertion of the *M*. *caudofemoralis longus* [97].

In caudal view, there is a well-developed longitudinal lateral crest (Fig 18), as observed in some dromaeosaurids [47, 100, 111, 112]. This crest constitutes the caudal intermuscular line that separates the insertion areas of *M*. *femorotibialis externus* (cranially) and *M*. *adductor femoris* (caudomedially). As in the holotype of *Bonapartenykus*, the lateral surface of the femur bears a large, sculptured bulge, which probably constitutes the scar for the insertion of the *M*. *iliofemoralis externus* [33, 97].

The caudal surface of the shaft is highly crushed, but the medial and lateral condyles are observable, which are mutually separated by a deep popliteal fossa, which in turn takes the form of a longitudinal groove (Fig 18). Presumably, the lateral condyle is slightly larger than the medial condyle (Fig 18). No femoral ectocondylar tuber is preserved.

**Fig 19:**
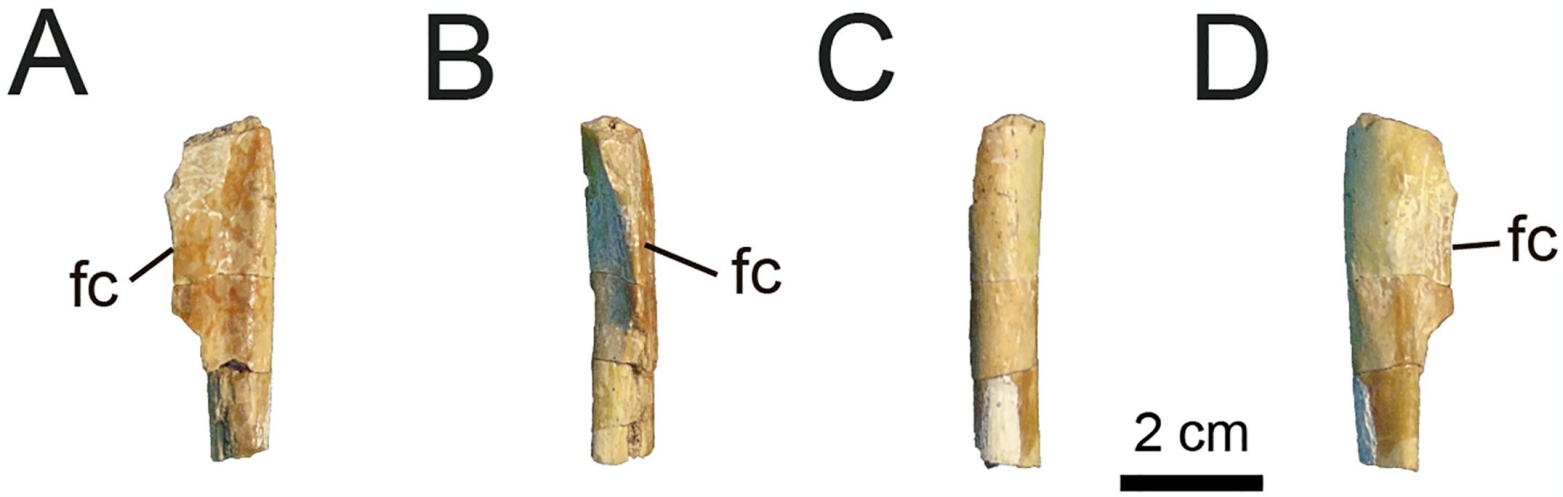
Right tibia of MPCN-PV 738.24. A, cranial view; B, lateral view; C, medial view; D, caudal view; E, proximal view; F, distal view. Abbreviations: fc, fibular crest. Scale bar equal to 2 cm.

*4*.*2*.*2*.*3*.*2*. *Tibia* (Fig 19): MPCN-PV 738.24 is represented by a proximal portion of the right tibial shaft. It is recognised primarily by the fibular crest, since it lacks its proximal joint surface. The shaft of the tibia of MPCN-PV 738.24 appears to be straight (Fig 19). Proximally, the shaft is subtriangular in cross-section (Fig 19), whereas distally it is subcircular. The fibular crest is strongly prominent and sharp (Fig 19), unlike in early diverging alvarezsaurians such as *Shishugounykus* [3], *Haplocheirus* [1], and *Xiyunykus* [6], some later-diverging forms such as *Mononykus*, *Shuvuuia*, and *Linhenykus* [69, 74, 75], or even Patagonian forms such as *Patagonykus* [36]. This crest rises on the lateral surface probably close to the proximal end, as occurs in other alvarezsaurians [1–3, 5, 6, 36, 69, 74]. In anterior view, the fibular crest has a quadrangular shape with a concave distal margin (Fig 19). In addition, it is strongly compressed craniocaudally (Fig 19).

The posterior surface of the tibial shaft is flat, whereas the medial surface is convex to slightly flat on the anteromedial surface. On the anterior surface, near the base of the fibular crest, there is a groove that is oriented proximodistally which becomes more marked distally. Proximally, this groove divides the shaft into two convex sections.

*4*.*2*.*2*.*3*.*3*. *Fibula* (S2 File: *“S10 Fig”)*: The fibula of MPCN-PV 738 is represented by a proximal end of a right element, which is highly eroded. Proximally, the fibular shaft is craniocaudally expanded and laterally compressed, becoming distally slender, as occurs in Patagonian forms [36] and in Parvicursorinae [69, 74, 75]. In proximal view, the fibula is crescentic in shape (S2 File: “S10 Fig”), being less cranially compressed than caudally (S2 File: “S10 Fig”), as occurs in *Patagonykus* [36]. In turn, the proximal articular surface is convex and posterodistally inclined, as observed in *Patagonykus*, and the medial surface is flat, unlike the condition present in specimen PVPH-37, where the medial surface is concave.

**Fig 20:**
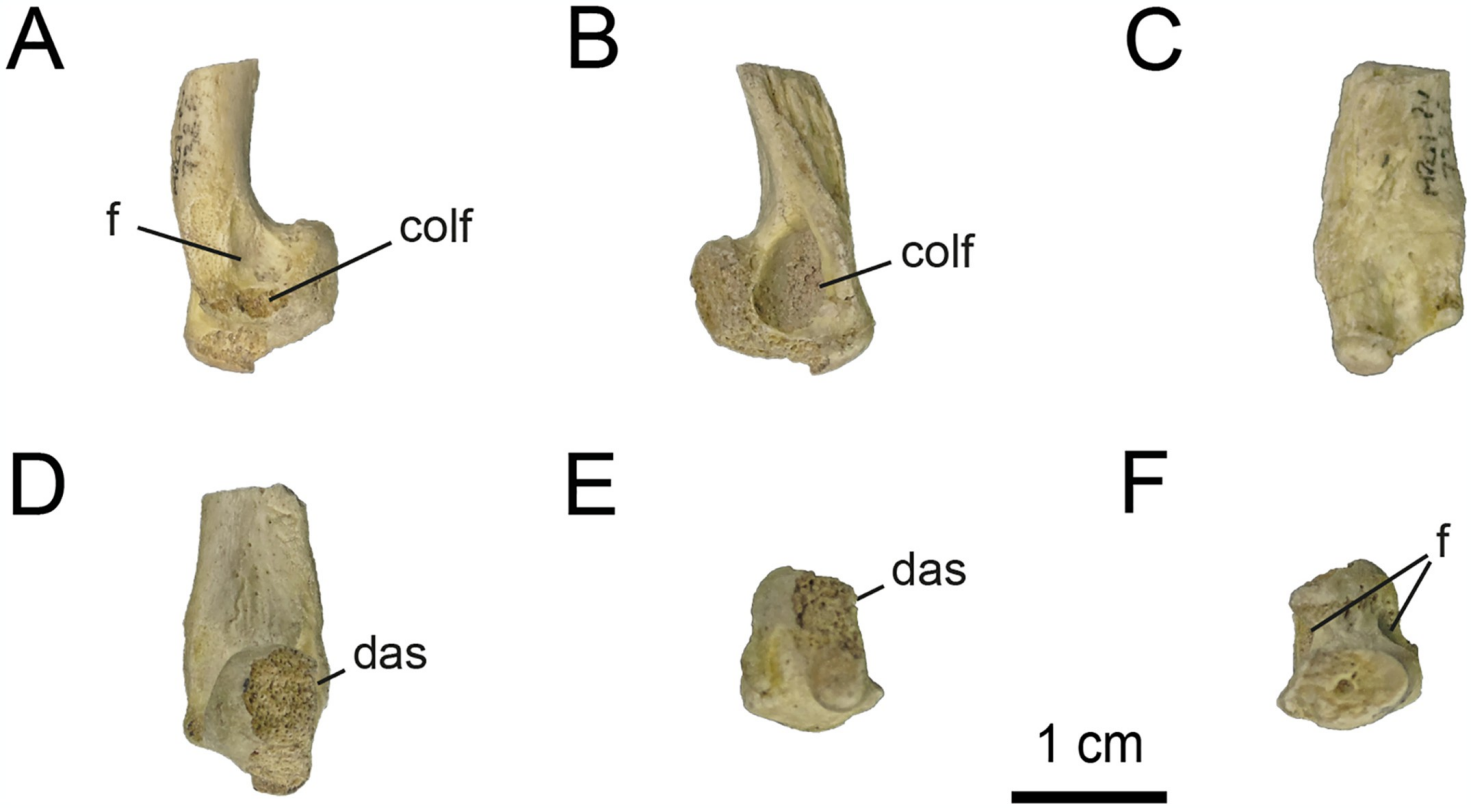
Right metatarsal I of MPCN-PV 738.6. A, lateral view; B, medial view; C, cranial view; D, caudal view; E, distal view; F, proximal view. Abbreviations: f, fossa; das, distal articular surface; colf, collateral ligament fossa. Scale bar equal to 1 cm.

*4*.*2*.*2*.*3*.*4*. *Metatarsal I* (Fig 20): Right metatarsal I is well-preserved, but it lacks its proximal end. Therefore, it is impossible to know the shape of its proximal end, although it probably would have been attenuated proximally where it articulated with metatarsal II, and would be a simple butt joint lying against the axis of metatarsal II, as in other alvarezsaurids (e.g., *Mononykus*, *Shuvuuia*, *Albinykus* and *Linhenykus*; [15, 20, 69, 75]), tyrannosauroids [66, 113], compsognathids [114], some ornithomimosaurians [115, 116], oviraptorosaurians [117, 118], dromaeosaurids [47, 103, 119, 120], and troodontids [83, 101, 121].

The shaft is proximodistally short as in most coelurosaurs and spike-like, craniocaudally compressed but expanded distally, as in some maniraptorans (e.g., *Buitreraptor*, *Neuquenraptor*, and *Kol*;[17, 112, 122]). The shaft is strongly craniocaudally compressed and transversely expanded (Fig 20), with the medial surface sharpened and the lateral surface rounded, as in unenlagiines. Proximodistally, the axis is slightly curved craniocaudally, unlike the ‘‘J” shaped bone of avialans [6, 17, 46, 95]. The posterolateral surface of the shaft is slightly flat, as opposed to the condition observed in Unenlagiinae [122]. Distally, on the lateral part of this element, there is a small, tab-shaped process, as in *Buitreraptor* and *Neuquenraptor*, although in the latter it is more developed (MPCA 245 and MCF PVPH 77, respectively).

In cranial or caudal view, the distal articular end is asymmetrical, with the lateral condyle more distally developed that the medial one. The lateral condyle is large and has a transversely expanded trochlear ring, where the two condyles are separated by a deep groove (Fig 20), a similar condition to some dromaeosaurids (e.g., *Neuquenraptor*, *Mahakala*, *Deinonychus*, *Velociraptor*, *Bambiraptor* and *Adasaurus*; [112, 119, 123–126]). In distal view, the articular end is strongly developed cranially, which is denoted in the shape of the rounded and robust condyle, which has a smooth surface (Fig 20). On both lateral and medial sides, there are the pits of collateral ligaments (Fig 20), different from *Kol*, where they converge caudally [17]. Both pits are centrally positioned, and have a sub-triangular contour, with the lateral pit being the larger and deeper, as occurs in *Buitreraptor* [122].

*4*.*2*.*2*.*3*.*5*. *Metatarsal II* (S2 File: *“S11 Fig”)*: Three fragments corresponding to the proximal, middle and distal sectors of metatarsal II are preserved (MPCN-PV 738.5). The proximal articular surface is slightly concave craniocaudally and expanded posteriorly with an oval contour. The medial margin is slightly convex, whereas the margin lateral is concave. This element has a subtriangular cross-section along its distal half (S2 File: “S11 Fig”). Thus, the anterior and medial surfaces of the shaft are slightly convex, whereas the lateral surface is flat and posteromedially inclined.

*4*.*2*.*2*.*3*.*6*. *Metatarsal III* (S2 File: *“S12 Fig”)*: Three elements are interpreted as fragments corresponding to the proximo-medial/distal sector of metatarsal III, representing three individuals probably from different ontogenetic stages. The two smallest fragments would correspond to the distal portion of a left metatarsal III, probably belonging to juvenile individuals by their proportions, whereas the largest fragment is the proximo-medial sector of a right metatarsal III, representing a larger individual. In general, the two smallest fragments are well-preserved, whereas the other is highly eroded.

Right metatarsal III of MPCN-PV 738 is elongated and slender, as in other Alvarezsauria [1, 2, 6, 15–17, 28, 74, 75]. It is slightly bowed craniocaudally (S2 File: “S12 Fig”), as in *Kol ghuva* ([17]; Fig 2). The cranial surface of the shaft is convex along its proximo-distal length, as in early diverging alvarezsaurids (i.e., *Haplocheirus*, *Bannykus*, *Alvarezsaurus*, and *Achillesaurus*). The distal portion of the cranial surface expands transversely, which can also be seen in the other two smaller fragments (S2 File: “S12 Fig”). This condition is observed only in some Parvicursorinae, such as *Kol*, *Mononykus*, *Albertonykus*, and *Linhenykus* [17, 19, 25, 75], and unenlagiines [122]. The caudal surface is clearly pinched, as occurs in intermediate alvarezsaurids (i.e., *Alvarezsaurus* and *Achillesaurus*), which produces a sinuous midline groove that defines the contact surfaces for metatarsals II and IV. These grooves for metatarsals II and IV run parallel and longitudinally along the shaft on the caudal surface of metatarsal III (S2 File: “S12 Fig”). There is no evidence of the proximal end of metatarsal III being fully concealed between metatarsals II and IV, as observed in Parvicursorinae. This morphology corresponds to a subarctometatarsalian condition, intermediate between the Patagonian forms and the Parvicursorinae [17, 19, 25, 28, 75].

The proximal end of the shaft is subtriangular in cross-section, as occurs in *Achillesaurus*, but not as compressed lateromedially as in this latter taxon. The condition in MPCN-PV 738 contrasts with that observed in early diverging forms such as *Haplocheirus*, *Bannykus*, *Alnashetri*, *Alvarezsaurus* and *Patagonykus*, where the shaft is strongly compressed, resulting in an elliptical cross-section. In MPCN-PV 738, the distal shaft is T-shaped in cross-section, as occurs in Parvicursorinae and the early diverging alvarezsaurid *Xiyunykus* ([6]; Fig 1), although in that taxon this condition is less pronounced.

*4*.*2*.*2*.*3*.*7*. *Pedal phalanges* (S2 File: *“S13*–*15 Figs”)*: Phalanx II-3 (ungual): The element MPCN-PV 738.46 was originally described by [32] as a right indeterminate ungual pedal phalanx. Based on the general morphology of *Mononykus*, *Shuvuuia*, *Parvicursor* and *Kol*, we here assign this element as a right ungual phalanx belonging to digit II of the foot. This element is partially complete, lacking its most distal portion.

MPCN-PV 738.46 is short craniocaudally compared to unguals III and IV, compressed transversely, and slightly curved distally (S2 File: “S13 Fig”), more than in later-diverging Asian forms. Its proximal articular surface is dorsoventrally much more extended than the ventral sector (S2 File: “S13 Fig”). Laterally on the proximoventral corner of the element, there is a flexor tubercle, which is moderately developed (S2 File: “S13 Fig”). The shaft is curved medially in dorsal view (S2 File: “S13 Fig”), and the lateral surface is much more convex than the medial surface, as occurs in other alvarezsaurids. As mentioned by [32], the lateral and medial surfaces bear grooves (S2 File: “S13 Fig”), but the external is much deeper and bifurcated further proximally than in *Patagonykus*.

Dorsally, both surfaces converge in a sharp edge or carina, which is distally displaced towards the medial surface. The same morphology occurs ventrally, with the difference that this edge is positioned on the opposite side. The proximal articular surface is much higher than wide, asymmetrical, and subtriangular in shape.

Phalanx III-1: MPCN-PV 738.22 is considered a proximal fragment of a right pedal phalanx III-1. This element only preserves its proximal-most sector. This phalanx is probably the largest of the pes, as occurs in other alvarezsaurids. In lateral view, this element thins distally, giving the entire conserved phalanx an isosceles shape (S2 File: “S14 Fig”), as occurs in other alvarezsaurids (e.g., *Kol ghuva*; [17]) and unenlagiines (e.g., *Neuquenraptor argentinus*; [112]). Unlike unenlagiines, the dorsal margin of phalanx III-1 of alvarezsaurids shows a small convexity proximally, and a thin distal end. Dorsally, the ventral surface is the more compressed of the two, giving the entire element an asymmetrical shape in dorsal view. The proximal articular surface is dorsoventrally convex, with both dorsal and ventral margins equally strongly developed. Likewise, the proximal surface is higher dorsoventrally than wide lateromedially, and asymmetric in contour (S2 File: “S14 Fig”). The anteroventral surface is slightly concave lateromedially, with medial and lateral crests of small size, only restricted to the most proximal sector.
Phalanx III-2: Originally, this piece was considered by [32] as a left pedal phalanx III-1. Here it is reinterpreted as a left pedal phalanx III-2 based on its dimensions and general morphology. This element lacks only the ventrolateral portion of the distal end.

MPCN-PV 738.18 is stouter and probably shorter than III-1. It is suboval in cross-section along the shaft, being wider than high. This morphology is different from that described for other alvarezsaurids (eg, *Shishugounykus* and *Kol*, [3, 17]), but similar to the morphology observed in Unenlagiinae (e.g., *Neuquenraptor*, [47]). Like the previous element, this phalanx becomes thinner distally when viewed laterally (S2 File: “S14 Fig”), but is wider transversely before the posterior articular surface. In addition, the ventral margin is strongly concave in lateral view (flexion of shaft slightly bow-shaped), as in other alvarezsaurids. Laterodistally, on both surfaces of the trochlea, a deep, well-defined, subelliptical collateral ligament pit is present (S2 File: “S14 Fig”).

The distal articular surface is wider than high, but is as wide as the proximal articular surface, and the condyles are well-developed. In dorsal view, a deep and craniocaudally extensive extensor depression can be observed, giving this depression a subtriangular shape (S2 File: “S14 Fig”). The shaft is straight and not bow-shaped, as occurs in phalanx III-1. Proximoventrally, there are small and robust ridges, defining a shallow but wide groove.

The proximal articular surface is asymmetrical, subpentagonal, and it is as wide as high (S2 File: “S14 Fig”). As in the anterior element, the dorsal and ventral margins are strongly developed.

Phalanx III-4 (ungual): MPCN-PV 738.44 was considered by [32] as an indeterminate ungual pedal phalanx; here it is interpreted as an ungual pedal phalanx belonging to digit III, based on its dimensions and morphology. Although it is a very fragmentary element, it is short and weakly arcuate (S2 File: “S14 Fig”). Its angle of curvature is lesser than in phalanx II-3 but greater than in phalanx IV-5, as occurs in other alvarezsaurids (e.g., *Kol ghuva*, [17]). Both grooves are well defined, and distally they are positioned ventrally.
Phalanx IV-2: The element MPCN-PV 738.23 is partially complete; it is short craniocaudally and robust. In lateral view, it as a triangular shape because of a dorsoventrally expanded proximal articular surface (S2 File: “S15 Fig”). Unlike *Kol*, the shaft is ventrally concave [17]. Distally, the collateral ligament pits are present, being very deep, tear-shaped, and centrally positioned (S2 File: “S15 Fig”). Although much of the dorsal sector is not preserved, the condyles would be asymmetric, on the basis on the groove that separates the two condyles. Proximoventrally, this phalanx is flat, unlike other alvarezsaurids and unenlagiines. The contour of its proximal articular surface is subquadrangular and strongly concave dorsoventrally.
Phalanx IV-3: The right phalanx IV-3 (MPCN-PV 738.19) is sub-equal in length and robustness to the preceding phalanx, but it is notably lateromedially compressed. It is strongly asymmetrical when viewed in dorsal view (S2 File: “S15 Fig”). On the medial side, and located proximally on the shaft, there is a well-developed bump (S2 File: “S15 Fig”), similar to that observed in *Neuquenraptor* [112]. The distal articular surface is ginglymoid, with the trochlear rings bearing slightly developed collateral ligament pits, which are located in the middle of the trochlea. In dorsal view, the extensor fossa is subtriangular, shallow and well-defined (S2 File: “S15 Fig”). The proximal articular surface is weakly concave, and the dorsal margin of the proximal articular surface does not extend anteriorly (S2 File: “S15 Fig”) in comparison with more proximal phalanges.
Phalanx IV-4: Two phalanges of different sizes are preserved; they are interpreted as right and left phalanges IV-4, probably representing two individuals of different sizes. Originally, the element MPCN-PV 738.21 was considered by [32] as a right pedal phalanx IV-3. However, the morphology of phalanx IV-4 present in MPCN-PV 738.21 is very distinctive from the rest.

In general, both phalanges are well preserved, although specimen MPCN-PV 738.21 lacks its proximodorsal portion. MPCN-PV 738.21 and 738.20 are characterised by being craniocaudally short and slightly longer than wide (S2 File: “S15 Fig”), as occurs in *Mononykus* and *Kol* [17, 75]. The proximal articular surfaces of both phalanges are strongly concave dorsoventrally, and convex laterally, and the dorsal margins of the surfaces extend cranially (S2 File: “S15 Fig”), different from *Mononykus*, *Shuvuuia* and *Kol* [15, 17, 75]. Unlike the Parvicursorinae, the distal articular surface is ginglymoid with the presence of a dorsal extensor groove. In turn, the medial and lateral surfaces of the distal joint are excavated by a round fossa, presumably for the insertion of collateral ligaments (S2 File: “S15 Fig”). Distally, on the ventral surface and on both sides of the longitudinal furrow, rounded ridges develop (S2 File: “S15 Fig”) as mentioned for *Kol* [17].

Phalanx IV-5 (ungual): MPCN-PV 738.45 was considered by [32] as an indeterminate ungual phalanx. Although this element is fragmentary, it is considered as an ungual pedal phalanx IV, on the basis on its slight ventral curvature (S2 File: “S15 Fig”), as in other alvarezsaurids. On both surfaces, longitudinal and probably symmetrical grooves are present. These are singular and do not extend to the distal margin.
Phalanges of indeterminate position: The fragments MPCN-PV 738.39 and 738.41 are proximal articular surfaces (S2 File: “S15 Fig”). These are subpentagonal and slightly concave, probably representing fragments of digit III. MPCN-PV 738.17 is small and short craniocaudally. While this piece lacks its dorsal portion, its morphology is similar to that of digit IV.

MPCN-PV 738.54 it is a proximodorsal fragment of an ungual phalanx, not originally described by [32]. This piece preserves most parts of its proximal articular surface, probably with subcircular contours. On both surfaces, the longitudinal grooves are observed.

## 5. Discussion

### 5.1. Egg level 2

Mineralogical analyses using X-ray diffraction on both the total rock powder and clay fraction from samples of MPCA 1290 and MPCN-PV 738 do not refute the hypothesis that the holotype of *Bonapartenykus ultimus* (MPCA 1290) and the fossil materials of MPCN-PV 738 come from the same site and stratigraphic level. To confirm this, XRD-ray diffraction profiles of all fossiliferous levels at the site should be collected and compared, but this work is beyond the scope of this study.

### 5.2. *Bonapartenykus ultimus*, MPCN-PV 738 and the patagonykine body plan

Overlapping material from these two collections of bones (the holotypic material of *Bonapartenykus ultimus* and the material collected by Salgado and his crew and accessioned under MPCN-PV 738) is limited to a scapulocoracoid, pubis, and a femur. As noted above, there are minor differences in these three bones that lead us to tentatively consider the referral of all of them to the same species.

[33] diagnosed *Bonapartenykus* by the strongly medially deflected ventral portion of the coracoid. This feature, however, is also present in *Patagonykus* and the parvicursorine *Xixianykus*, which indicates it is a synapomorphy of Patagonykinae that was convergently acquired by *Xixianykus*. The scapulocoracoids of MPCA 1290–7 (left scapulocoracoid of *Bonapartenykus*) and MPCN-PV 738.2 (and *Patagonykus*) have an angle of the medially deflected coracoid that ranges between 117° to 120°, whereas in MPCA 1290–1 (right scapulocoracoid of *Bonapartenykus*) it is 109°. In this sense, in cranial view, the angle of the medially deflected coracoid is slightly greater in MPCA 1290–1 (L-shaped) than in MPCA 1290–7 and MPCN-PV 738.2 (or even *Patagonykus*). This morphological feature is correlated with the morphology of the transversal ridge that is craniocaudally developed on the lateral surface of the coracoid. In MPCA 1290–7 and MPCN-PV 738.2, this ridge is robust and dorsoventrally broad (arc-like in cranial or caudal view), unlike MPCA 1290–1 where it is prominent and laterally developed (V-shaped in cranial or caudal view) and culminates cranially in a well-developed process followed by a notch. Additionally, the deep and narrow grooves and bumps that are present on the ventral surface of the coracoid of MPCA 1290–7 and MPCN-PV 738.2 do not surpass the level of the longitudinal ridge, contrary to the condition observed in MPCA 1290–1, in which deep and narrow grooves and bumps develop dorsal to the longitudinal ridge (over the proximal region of the dorsal surface of the coracoid).

In MPCA 1290–1 the coracoid part of the glenoid cavity is longer and wider than in MPCA 1290–7 and probably MPCN-PV 738.2. In MPCA 1290–7 and MPCN-PV 738.2, the angle between the anterior margin of the scapula and the dorsal margin of the coracoid does not exceed 90°, whereas in MPCA 1290–1 this angle is ~105°.

The coracoid foramen of MPCA 1290–1 is elliptical, with its major axis in a anterodorsal-posteroventral direction, but in MPCA 1290–7 and MPCN-PV 738.2 this orientation is difficult to determine. In scapulocoracoids MPCA 1290–7 and MPCN-PV 738.2, this foramen is located cranioventrally with respect to the glenoid cavity, contrasting with the condition observed in MPCA 1290–1, where its location is restricted ventrally with respect to the glenoid cavity. It is worth mentioning that these locations are with respect to the orientation of the scapula considered in this study (see Material and methods).

The craniocaudal length/dorsoventral height ratio of the coracoid differs in the *Bonapartenykus* holotype (r = 1.67) and MPCN-PV 738.2 (r = 1.45), but this proportional difference may vary ontogenetically. This difference in the angle formed by the longitudinal ridge of the coracoid that is observed in the holotype of *Bonapartenykus* and MPCN-PV 738.2 is also observed in some coelurosaurs [127].

In general, the morphology of the pubic shaft in MPCN-PV 738.4 is dorsoventrally elongated and mediolaterally slender, contrary to the condition observed in the holotype of *Bonapartenykus ultimus* whose shaft is mediolaterally broader. In the pubis of MPCN-PV 738.4, the shaft is caudoventrally curved, arc-like, with a convex anterior margin and a concave posterior margin. However, the shaft in the holotype of *Bonapartenykus ultimus* is subtly curved cranially, with a slightly straight to slightly convex anterior margin and a concave posterior margin, and is distally straight. In cranial or caudal view, the pubic shaft of MPCN-PV 738.4 is sigmoid, unlike the holotype of *Bonapartenykus ultimus* whose shaft is distally straight. Besides, the pubis in the holotype of *Bonapartenykus ultimus* is strongly opisthopubic, whereas the proximal end preserved in MPCN-PV 738.4 could indicate a slightly mesopubic condition (similar to that seen in *Patagonykus*).

Although the femur described here is slightly damaged, and the femur in the *Bonapartenykus* holotype is incompletely known, we consider that MPCN-PV 738.1 may well correspond to a larger individual of the same species. As mentioned by [41], the appendicular elements of the holotype of *Bonapartenykus* (e.g., estimated femur length of 173 mm) indicate that this specimen would have a body mass of around 43.75 kg and a body length of 3.06 m, whereas the MCPN-PV 738.1 femur (length = 304 mm) suggests that this specimen would have a larger body mass of around 59.88 kg and a larger body length of 3.31 m (see [41]).

Based on the comparison detailed above and the subtle nature of the noted differences, we currently consider all the elements catalogued under accession number MPCN-PV 738 as cf. *Bonapartenykus ultimus*. These materials and those of the holotype of *Bonapartenykus ultimus* show differences that can be plausibly interpreted as individual variation, so for the moment we favor the existence of a single alvarezsaurid species at this locality.

If there is only one species at this locality, the specimens analysed here would make *Bonapartenykus* the best known alvarezsaurid from South America, including the presence of elements of different sizes that could be indicative of different ontogenetic stages. Moreover, many of the mentioned characters that differ between overlapping elements could be explained by allometric differences in the size of muscular features across the developmental trajectory of the taxon (e.g., coracoid). In this sense, the availability of morphological variation in this assemblage may be due to ontogeny, sexual dimorphism, or just individual intraspecific variation that should be studied further.

An alternative hypothesis identifying more than a single species at the locality would provide evidence of a sympatric and synchronous association of alvarezsaurids in the ecosystem represented by the Allen Formation. The coexistence of more than one alvarezsaurian species has already been documented in multiple formations including the Bajo de la Carpa Formation in Argentina as well as the Shishugou, Baruungoyot, Djadokhta, Nemegt, as well as in the Tugulu Group [1–3, 12, 20, 41, 75, 93]. To clearly distinguish between these two hypotheses, future fieldwork at the Arriagada III locality to recover additional alvarezsaurid material would be invaluable.

### 5.3. Phylogenetic position of MPCN-PV 738

**Fig 21:**
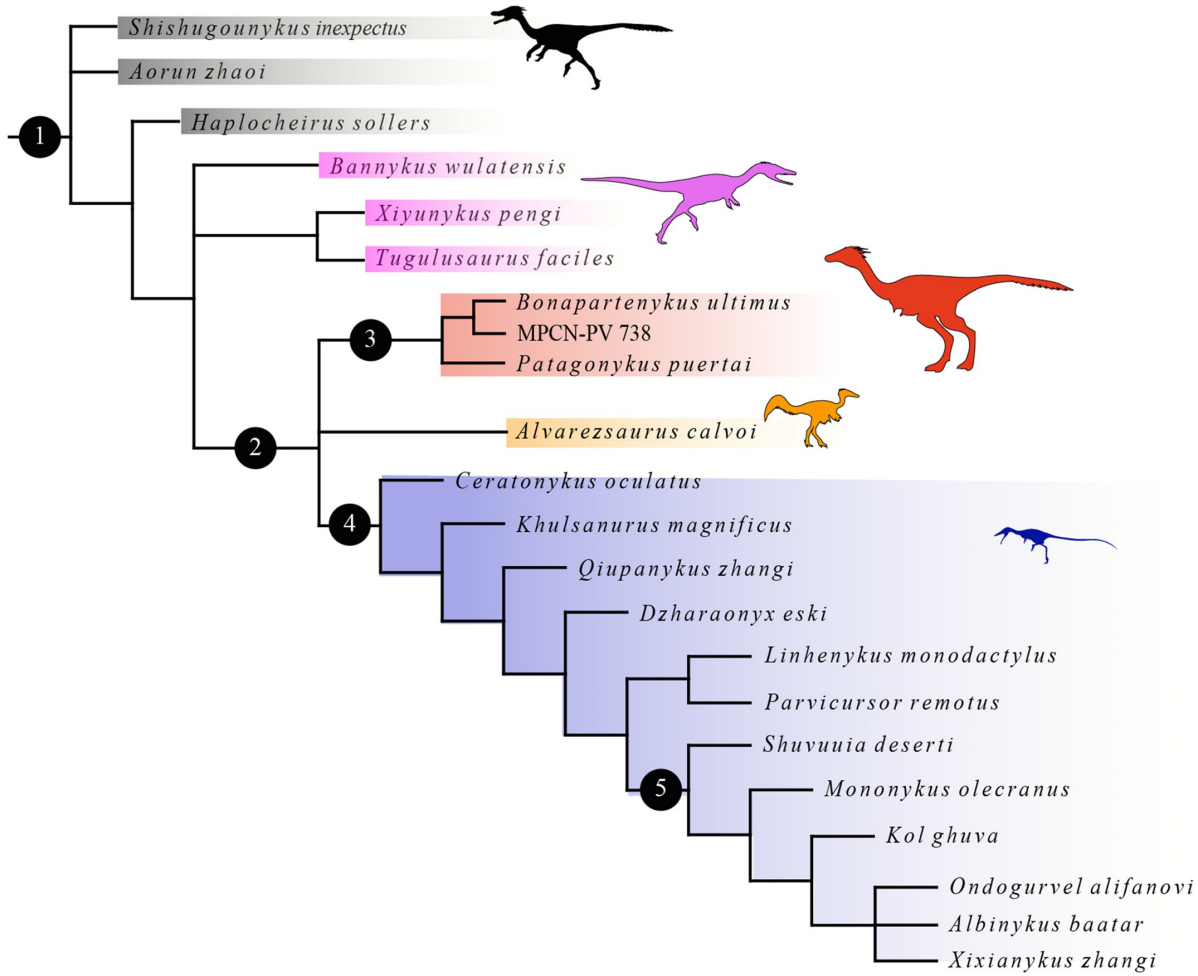
Detail of alvarezsaurian interrelationships from the reduced consensus (3313 steps, consistency index = 0.223, retention index = 0.611) with *Heptasteornis*, *Albertonykus*, *Nemegtonykus*, *Trierarchuncus*, *Stokesosaurus*, *Segnosaurus*, *Gigantoraptor*, *Unenlagia* plus *Neuquenraptor*, *Sinornithoides*, *Qiupanykus* and *Achillesaurus* excluded posteriori to increase resolution. 1, Alvarezsauria; 2, Alvarezsauridae; 3, Patagonykinae; 4, Parvicursorinae; and 5, Mononykini.

The phylogenetic analysis resulted in 999,999 most parsimonious trees (MPTs) of 3393 steps, a Consistency Index (CI) of 0.223, and a Retention Index (RI) of 0.611, which combined into a strict consensus tree revealing a large maniraptoriform polytomy. The IterPCR methodology identified eleven unstable taxa that were excluded from the reduced consensus (i.e., *Heptasteornis*, *Albertonykus*, *Nemegtonykus*, *Trierarchuncus*, *Stokesosaurus*, *Segnosaurus*, *Gigantoraptor*, *Unenlagia* plus *Neuquenraptor*, *Sinornithoides*, *Qiupanykus*, and *Achillesaurus*). The reduced consensus produced a novel and well-resolved topology for Alvarezsauria (Fig 21), and the implementation of the pcrjak script identified a significant increase in Bremer support values for several nodes within Alvarezsauria. A more detailed analysis of the phylogenetic results of the clade will be treated elsewhere, but here we will discuss the evidence related to the phylogenetic position of the materials catalogued under MPCN-PV 738 and its bearing on their taxonomic identification.

All of the materials assigned to MPCN-PV 738 were scored as a single terminal to determite its phylogenetic position. *Bonapartenykus* was scored solely based on the holotype (S2 File: “Data Matrix”). MPCN-PV 738 is unequivocally retrieved as the sister taxon of *Bonapartenykus*, and both are nested within Patagonykinae along with *Patagonykus puertai* (Fig 21). Four synapomorphies support the monophyly of Patagonykinae: spinopostzygapophyseal lamina in dorsal vertebrae well developed and terminates abruptly on dorsal surface of postzygapophysis (302:1); a well-developed lateral ridge extending along of margin lateral on the coracoid (363:1); coracoid with ventral portion directed ventromedially at obtuse angle to dorsal portion (365:1); and pubic boot with projection anteriorly and posteriorly (482:0). All of these characters are present in MPCN-PV 738, justifying its patagonykine affinities. The characters that support the sister group relationship of *Bonapartenykus ultimus* with MPCN-PV 738 are as follow: postzygapophyses of the dorsal vertebrae with small, flange-like medial extensions of the postzygapophyseal facets (298:1); ventral half of lateral surface of coracoid with rugose and heavily textured cortical bone surface (364:1); medially deflected coracoid less than 110° (645:2); and coracoid foramen has an elliptical shape, with the main axis oriented in an anterodorsal-posteroventral direction (646:1).

### 5.4. New insights into the patagonykine body plan

Previously, the body plan of the Patagonykinae was largely limited to the remains preserved in *Patagonykus puertai*, which included an incomplete pectoral girdle, forelimb, hindlimb, and tail. MPNC-PV 738 provides new information on previously unknown aspects of the patagonykine body plan, aiding in its reconstruction. Regarding the axial skeleton, we now have a better understanding of the neck and cervicodorsal transition, with the development of prominent structures and fossae suggesting a robust neck compared to the more simplified and gracile cervical vertebrae of the Parvicursorinae. We now see that the patagonykine sacral series exhibits significant pneumaticity. Additional materials have been described for the tail region revealing characters unique to Patagonian forms. Only a few anatomical characters observed may be related to ontogenetic variation which concern the pectoral girdle. In the hindlimb, newly added information includes a partially complete metatarsal I, the distal end of metatarsal III showing a transition from a sub-arctometatarsalian to an arctometatarsalian condition, and pedal phalanges suggesting a symmetrical foot morphology associated with locomotor advantages.

### 5.5. Bioerosion traces

The holotype of *Bonapartenykus ultimus* MPCA 1290 and specimens collectively catalogued under MPCN-PV 738, exhibit different types of fossil traces. For example, in the holotype of *Bonapartenykus ultimus*, there are "puncture" marks on the surface of all bones. In the elements assigned to MPCN-PV 738, there are two types of fossil traces, "parallel-edge furrows" and "overlapped grooves". These trace differences are suggestive of taphonomic differences e.g., differences in how and when they were exposed and buried. It has been argued that highly disjointed skeletons suggest significant subaerial exposure [128, 129]. The morphology of the traces classified as "punctures" coincide with large, robust and conical teeth. The only theropods known so far from the Allen Formation are abelisaurids, alvarezsaurids, and dromaeosaurids. The teeth of both alvarezsaurids and dromaeosaurids are small, transversely compressed and lack denticles with usually less than 2 millimetres between teeth (e.g., [41, 130]). The large size of the ‘punctures’ and the wider spacing between them therefore fits best with the known abelisaurids of the formation. The "parallel-edge furrows" and "overlapped grooves" were most likely produced by a mammal because they had the active scavenging behavior to produce these type of marks [131], although scavenging behaviour remains very understudied in theropods.

## 6. Conclusions

In the present study, we redescribe alvarezsaurid fossil material collected and partially described by [32], and describe alvarezsaurid fossil materials collected at the same site by different expeditions for the first time. XRD data and excavation notes support the assignment of all elements catalogued under MPCN-PV-738 to the same site and stratigraphic level as the holotype of *Bonapartenykus ultimus* (MPCA 1290). Character data and phylogenetic analysis results support the patagonykine affinities of the MPCN-PV-738 material and its tentative referral to *B*. *ultimus*. Among this expanded material of *B*. *ultimus*, individuals of different sizes and possibly different ontogenetic stages are represented. However, owing to the fragmentary nature of overlapping elements and the subtle nature of morphological differences, a definitive referral to *B*. *ultimus* is not possible for the moment.

Our analysis of the different pattern of biotraces in MPCA 1290 and MPCN-PV 738 indicates that this association represents an assemblage of alvarezsaurid bones with different taphonomic histories e.g., differences in how and when they were exposed and buried.

**Fig 22:**
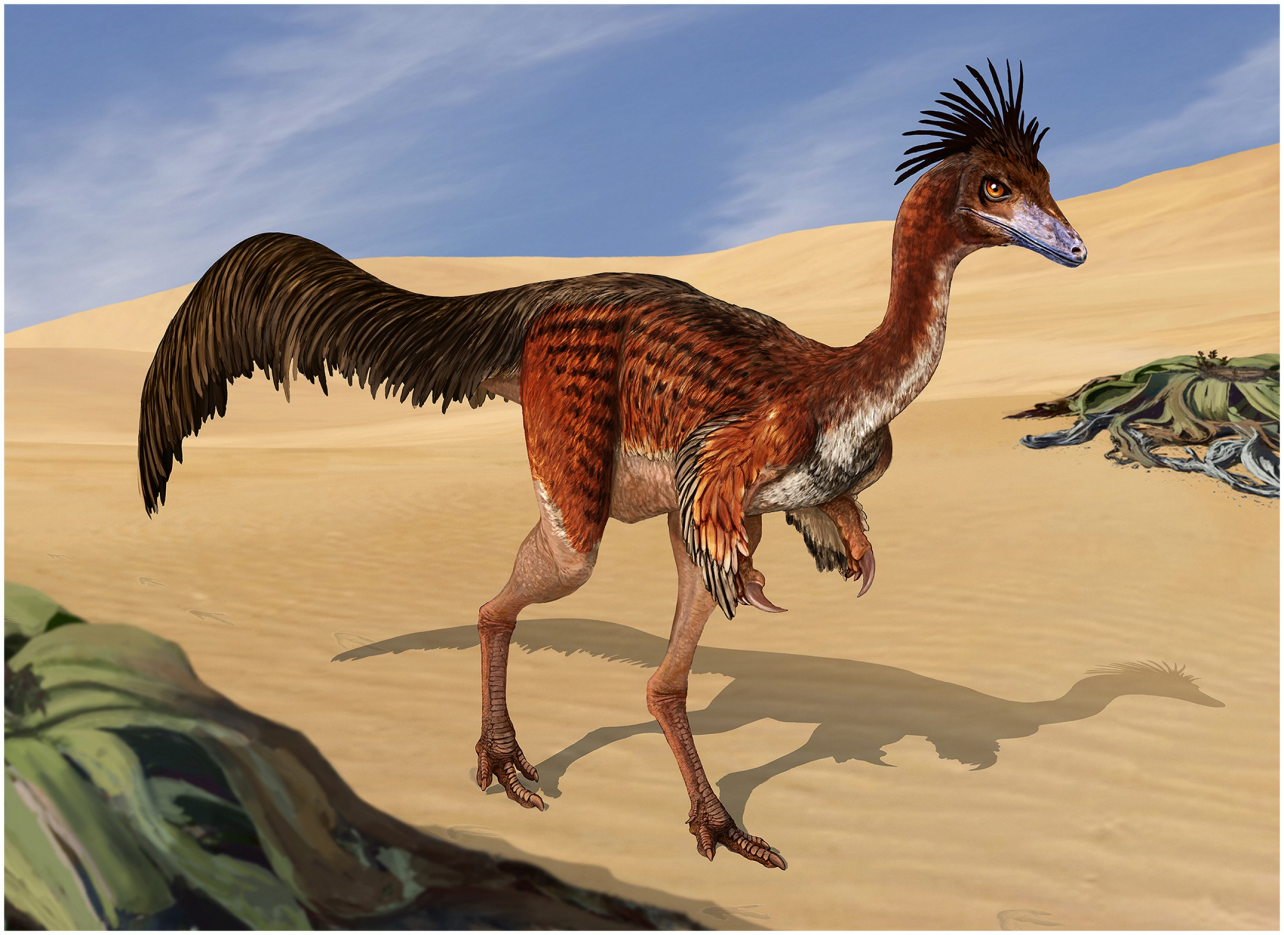
Life reconstruction of an individual of MPCN-PV 738. Artwork by Abel G. Montes.

While our results favor a monospecific alvarezsaurid assemblage for the moment, future research and quarrying efforts at the Salitral Ojo de Agua locality may unearth evidence that can further test our current interpretation. The elements described here as MPCN-PV 738 shed new light on the body plan of Patagonykinae, and allow a more precise reconstruction of the neck, pectoral girdle, hindlimb and tail of this clade (Fig 22).

## Supporting information

S1 File Characters, modifications, and characters ordered. (XLSX)

S2 File XRD study of sediments, morphological description, and data matrix. (DOCX)
