# Supplementary material for: New information on Bonapartenykus (Alvarezsauridae: Theropoda) from the Allen Formation (middle Campanian-lower Maastrichtian) of Río Negro Province, Patagonia, Argentina clarifies the Patagonykinae body plan
Source: PLoS One. 2025 Jan 30;20(1):e0308366. doi: 10.1371/journal.pone.0308366 (PMC11781669; doi:10.1371/journal.pone.0308366)
Supplement: S2 File — (DOCX) [file pone.0308366.s002.docx]

**S2. Supplemental information and figures for “New information on *Bonapartenykus* (Alvarezsauridae: Theropoda) from the Allen Formation (Campanian-Maastrichtian), Río Negro province, Patagonia, Argentina clarifies reconstruction of Patagonykinae body plan”.**

**A. XRD STUDY OF SEDIMENTS**

**B. MORPHOLOGICAL DESCRIPTION**

**C. DATA MATRIX**

**A. XRD STUDY OF SEDIMENTS**

**
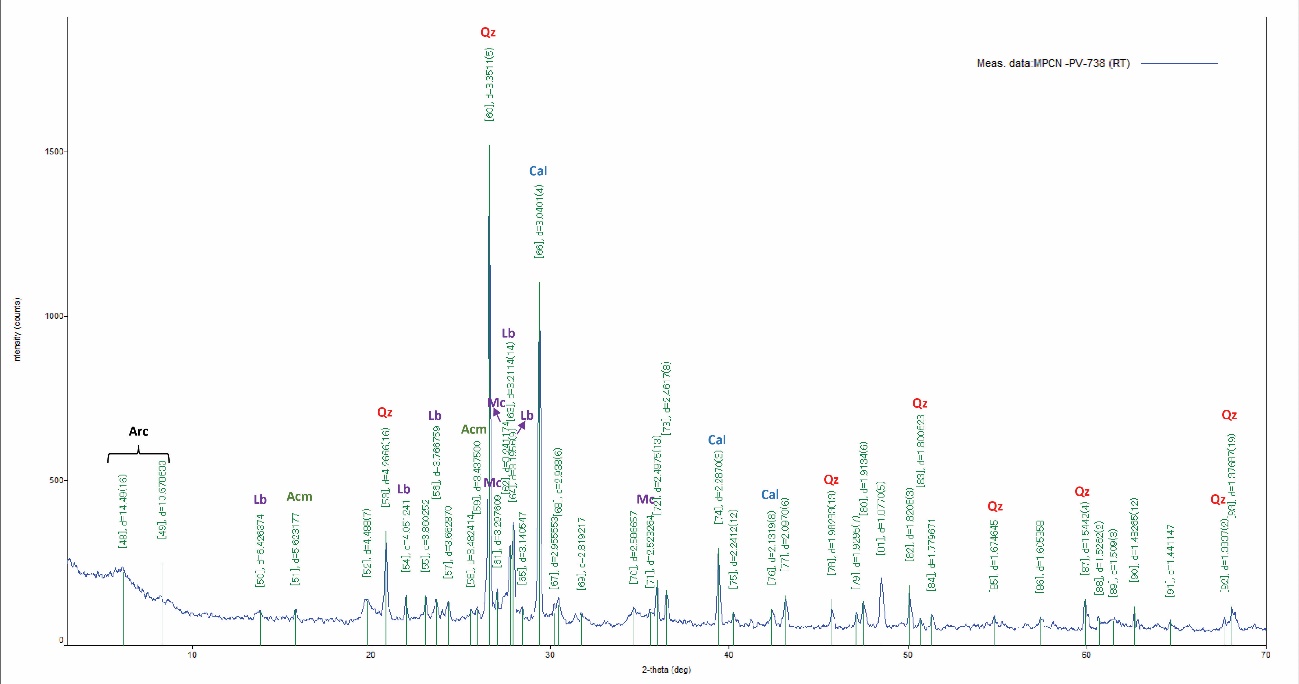
**

**FIGURE S1.** Total rock diffractogram for sample MPCN-PV-738. Abbreviations: Qz, quartz; Lb, labradorite; Mc, microcline; Acm, analcima; Cal, calcite; Arc, clays.

**
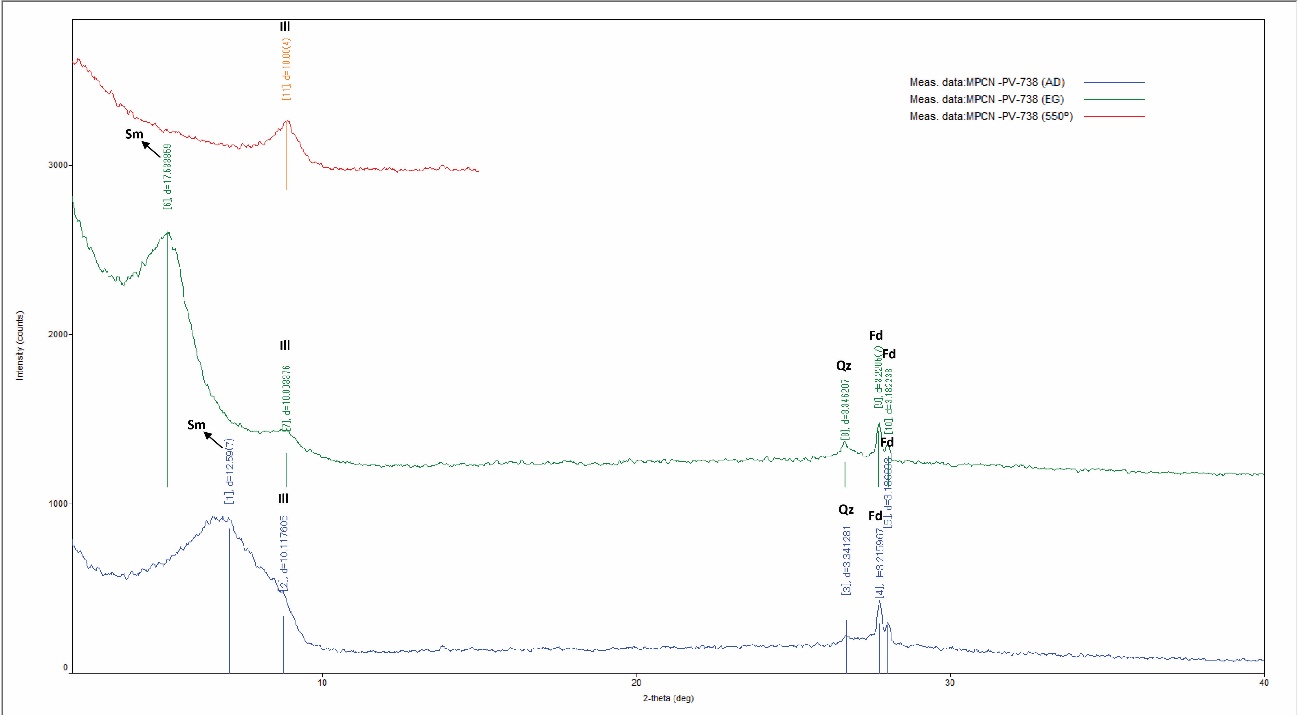
**

**FIGURE S2.** AD diffractograms: air-dried; EG: glycol; 550º: calcined, corresponding to the clay fraction of the sample MPCN-PV-738. Abbreviations: Smc, smectite; Ill, illite; Qz: quartz; Fd, feldspar.

**
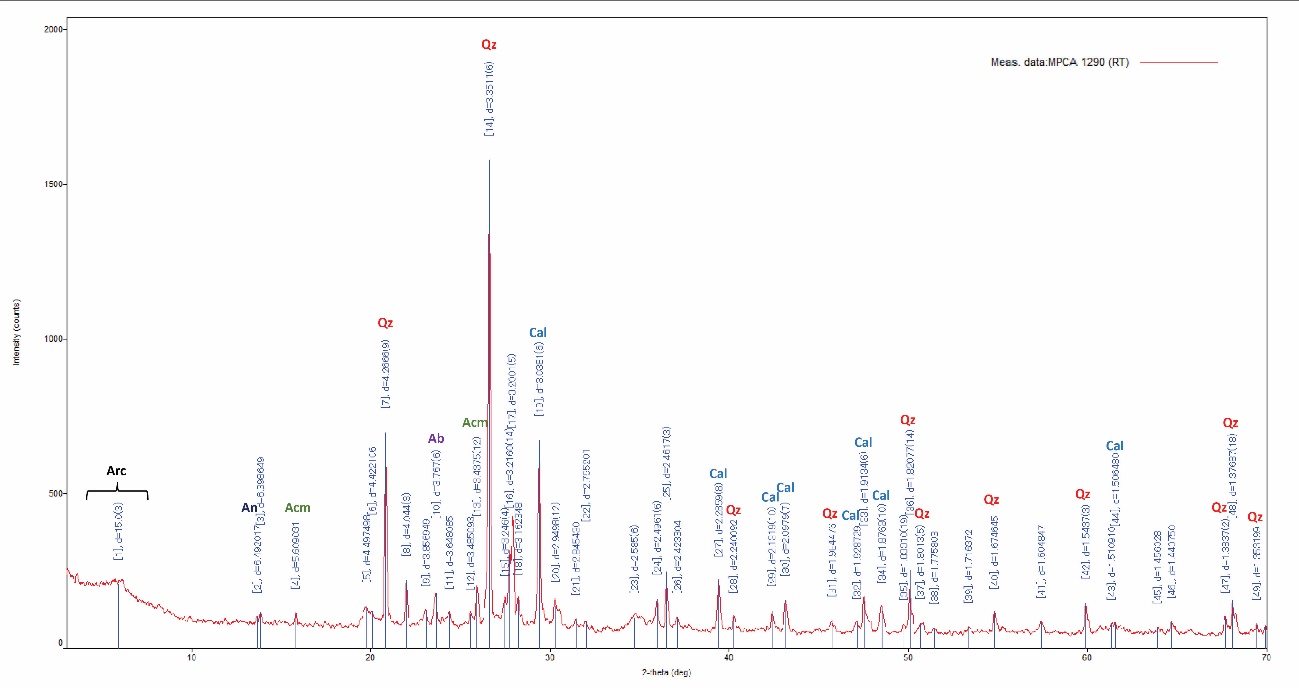
**

**FIGURE S3.** Total rock diffractogram corresponding to the MNCA 1290 sample. Abbreviations: Qz, quartz; Pg, plagioclase; Fd, feldspar; An, analcima; Ba, barite; Cal, calcite; Arc, clays.

**
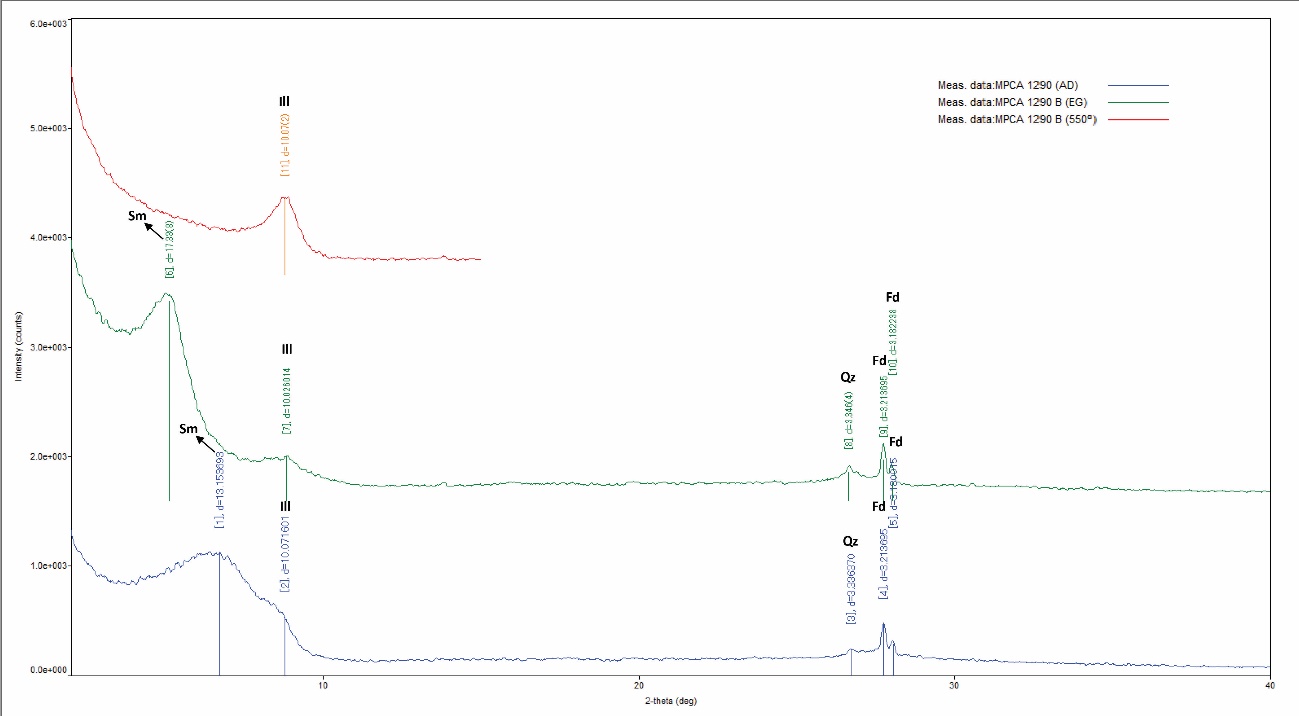
**

**FIGURE S4.** AD diffractograms: air-dried; EG: glycol; 550º: calcined, corresponding to the clay fraction of the sample MPCA-1290. Abbreviations: Smc, smectite; Ill, illite; Qz, quartz; Fd, feldspar.

**B. MORPHOLOGICAL DESCRIPTION**

**
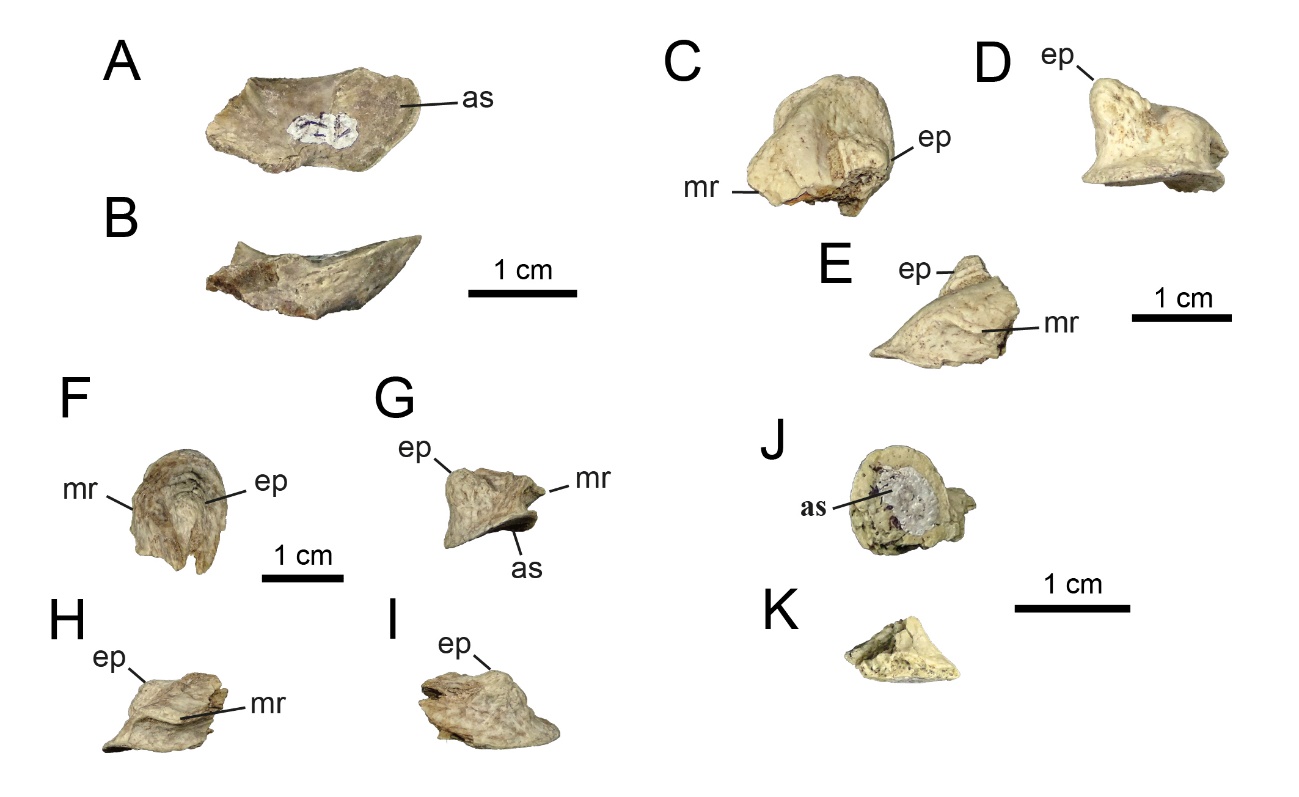
**

**FIGURE S5.** Zygapophyses of middle-posterior cervical vertebrae of MPCN-PV 738. A, MPCN-PV 738.51 in dorsal view; B, MPCN-PV 738.51 in lateral view; C, MPCN-PV 738.50 in dorsal view; D, MPCN-PV 738.50 in caudal view; E, MPCN-PV 738.50 in medial view; F, MPCN-PV 738.48 in dorsal view; G, MPCN-PV 738.48 in caudal view; H, MPCN-PV 738.48 in medial view; I, MPCN-PV 738.48 in lateral view; J, MPCN-PV 738.55 in ventral view; K, MPCN-PV 738.55 in medial view. Abbreviations: as, articular surface; mr, medial ridge; ep, epipophysis. Scale bar equal to 1 cm.


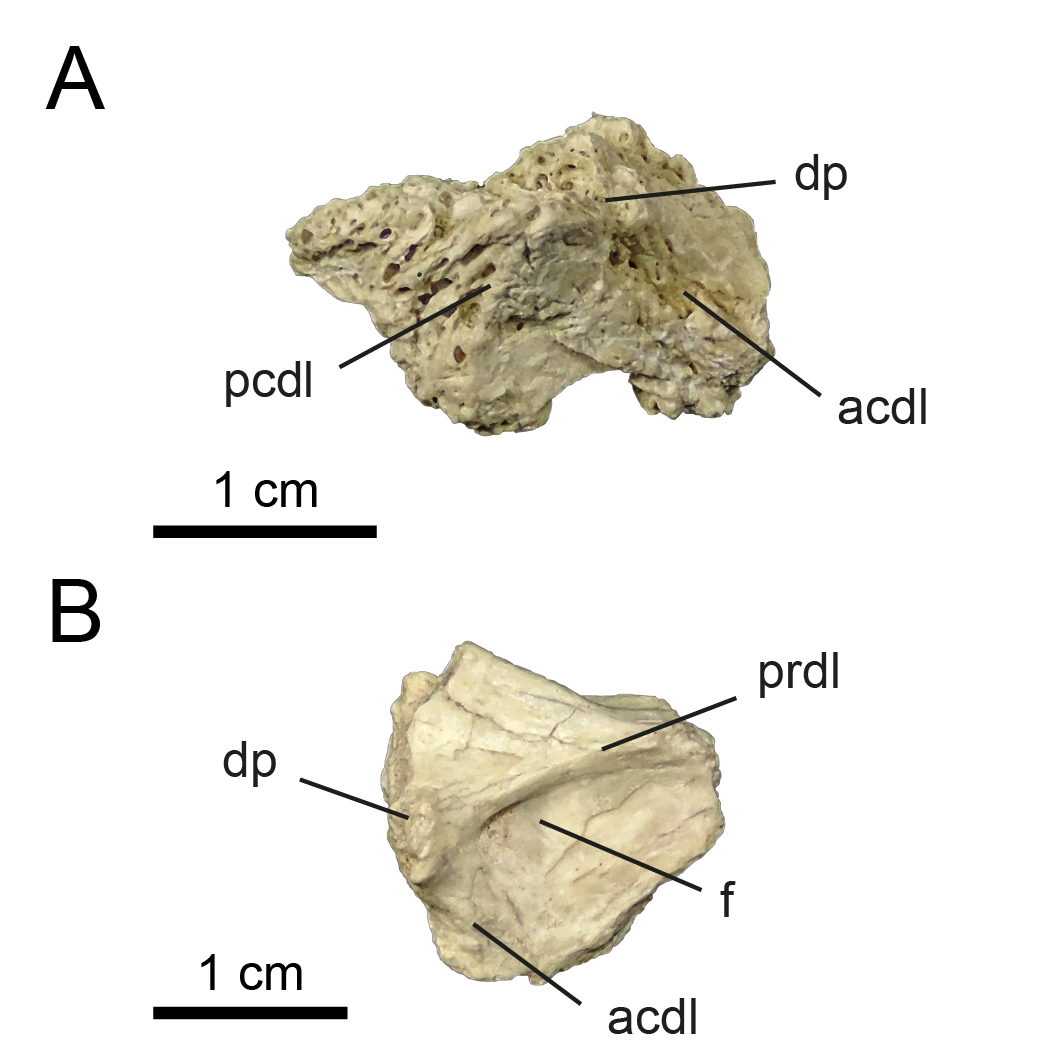


**FIGURE S6.** Fragment neural arches of cervical vertebrae of MPCN-PV 738. A, MPCN-PV 738.52 in lateral view; and B, MPCN-PV 738.43 in lateral view. Abbreviations: pcdl, posterior centrodiapophyseal lamina; acdl, anterior centrodiapophyseal lamina; prdl, prezygodiapophyseal lamina; dp, diapophysis; f, fossa. Scale bar equal to 1 cm.


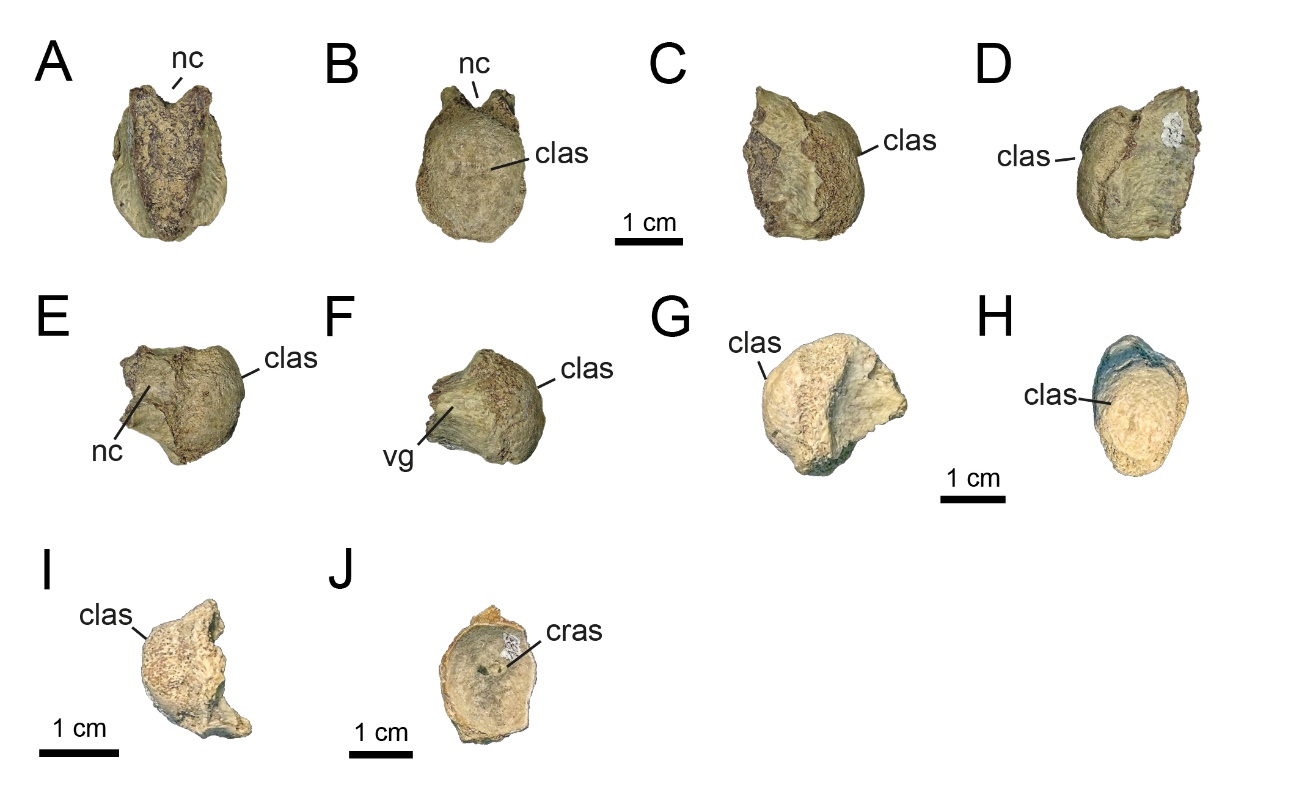


**FIGURE S7.** Caudal vertebrae of MPCN-PV 738. A, MPCN-PV 738.30 in cranial view; B, caudal view; C, MPCN-PV 738.37 in left lateral view; D, right lateral view; E, dorsal view; F, ventral view; G, MPCN-PV 738.38 right lateral view; H, caudal view; I, MPCN-PV 738.40 right lateral view; J, cranial view. Abbreviations: clas, caudal articular surface; cras, cranial articular surface; nc, neural channel; vg, ventral groove. Scale bar equal to 1 cm.


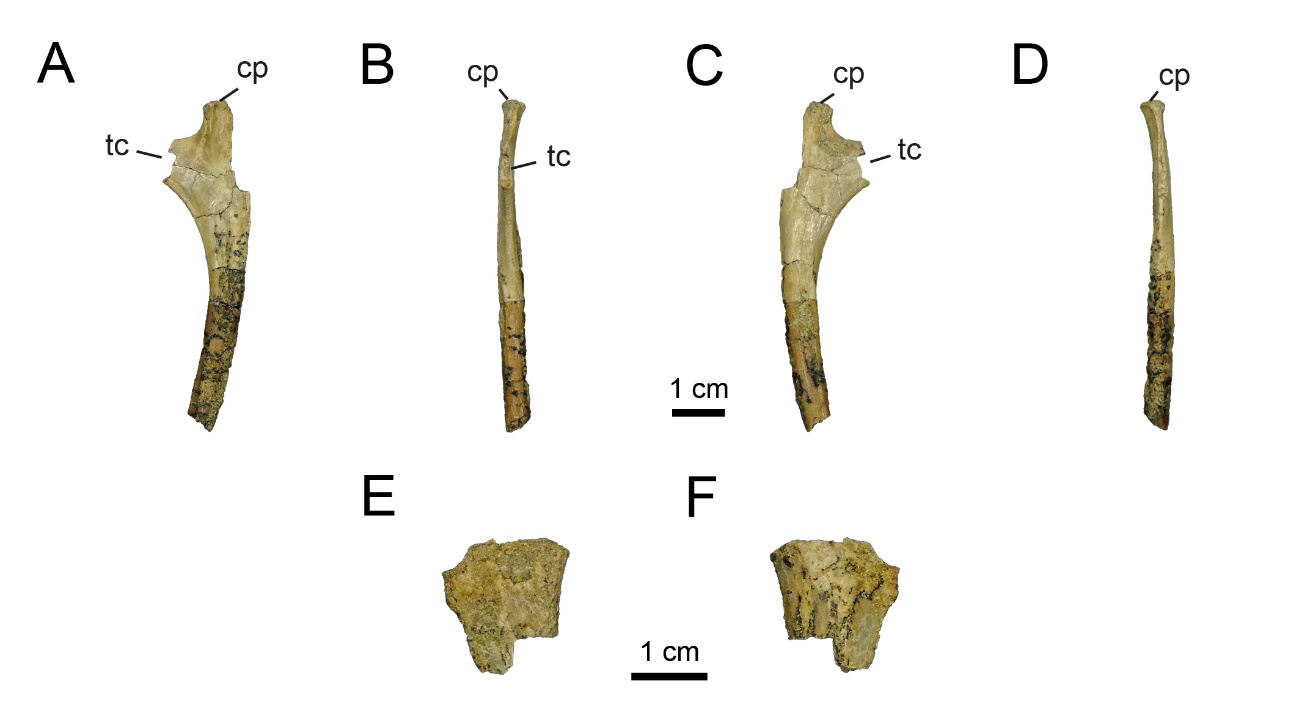


**FIGURE S8.** Dorsal rib of MPCN-PV 738. A, MPCN-PV 738.36 in cranial view; B, medial view; C, caudal view; D, lateral view. E and F, MPCN-PV 738.35 in cranial and caudal view view. Abbreviations: cp, capitulum; etc, tuberculum. Scale bar equal to 1 cm.


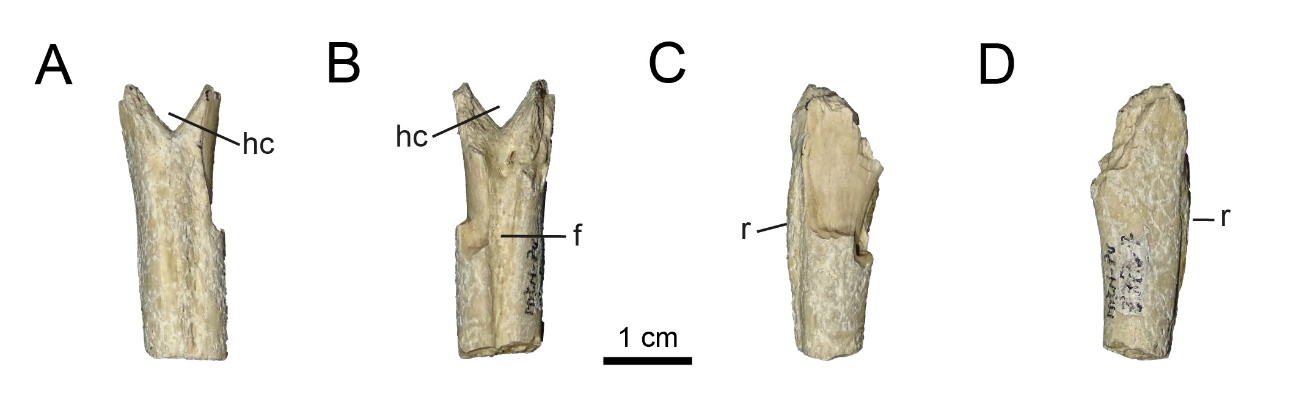


**FIGURE S9.** Proximal-middle chevron of MPCN-PV 738.7. A, cranial view; B, caudal view; C, left lateral view; D, right lateral view. Abbreviations: hc, haemal channel; r, ridge; f, fossa. Scale bar equal to 1 cm.


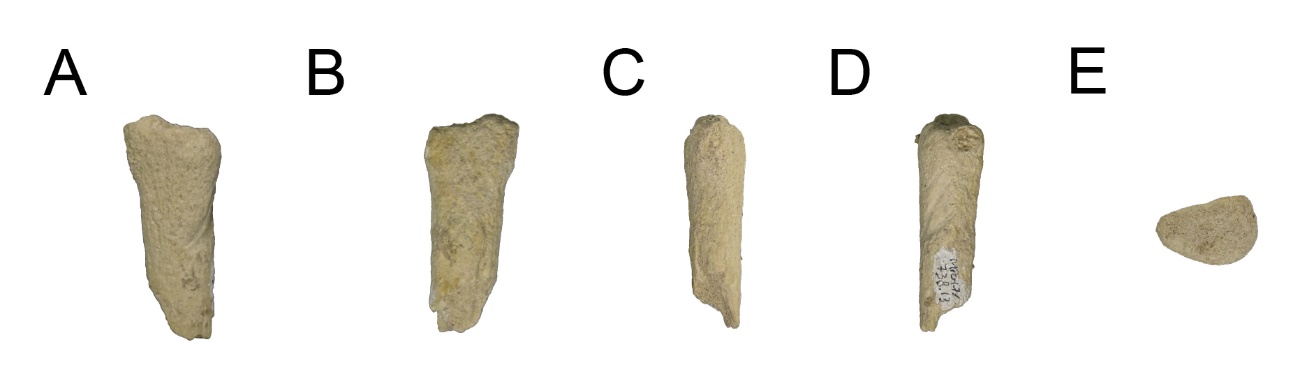


**FIGURE S10.** Right fibula of specimen MPCN-PV 738.13. A, lateral view; B, medial view; C, cranial view; D, caudal view; E, proximal view. Scale bar equal to 2 cm.


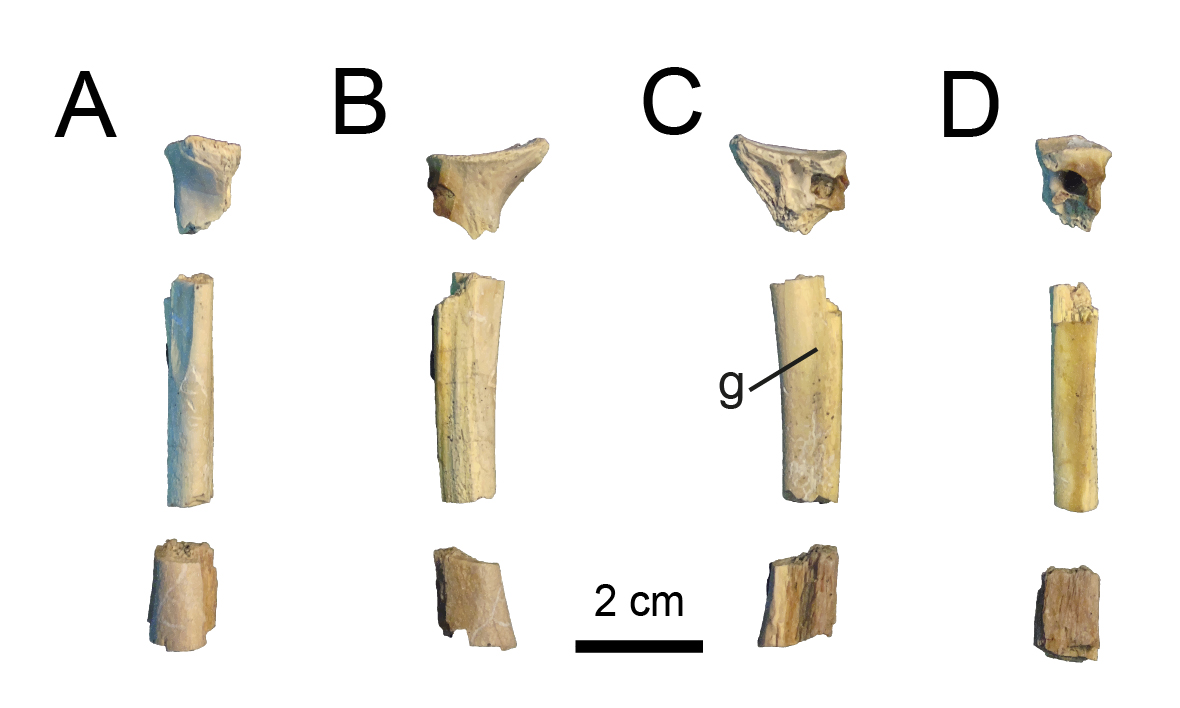


**FIGURE S11.** Left metatarsal II of MPCN-PV 738.5. A, cranial view; B, medial view; C, lateral view; D, caudal view. Abbreviations: g, groove. Scale bar equal to 2 cm.


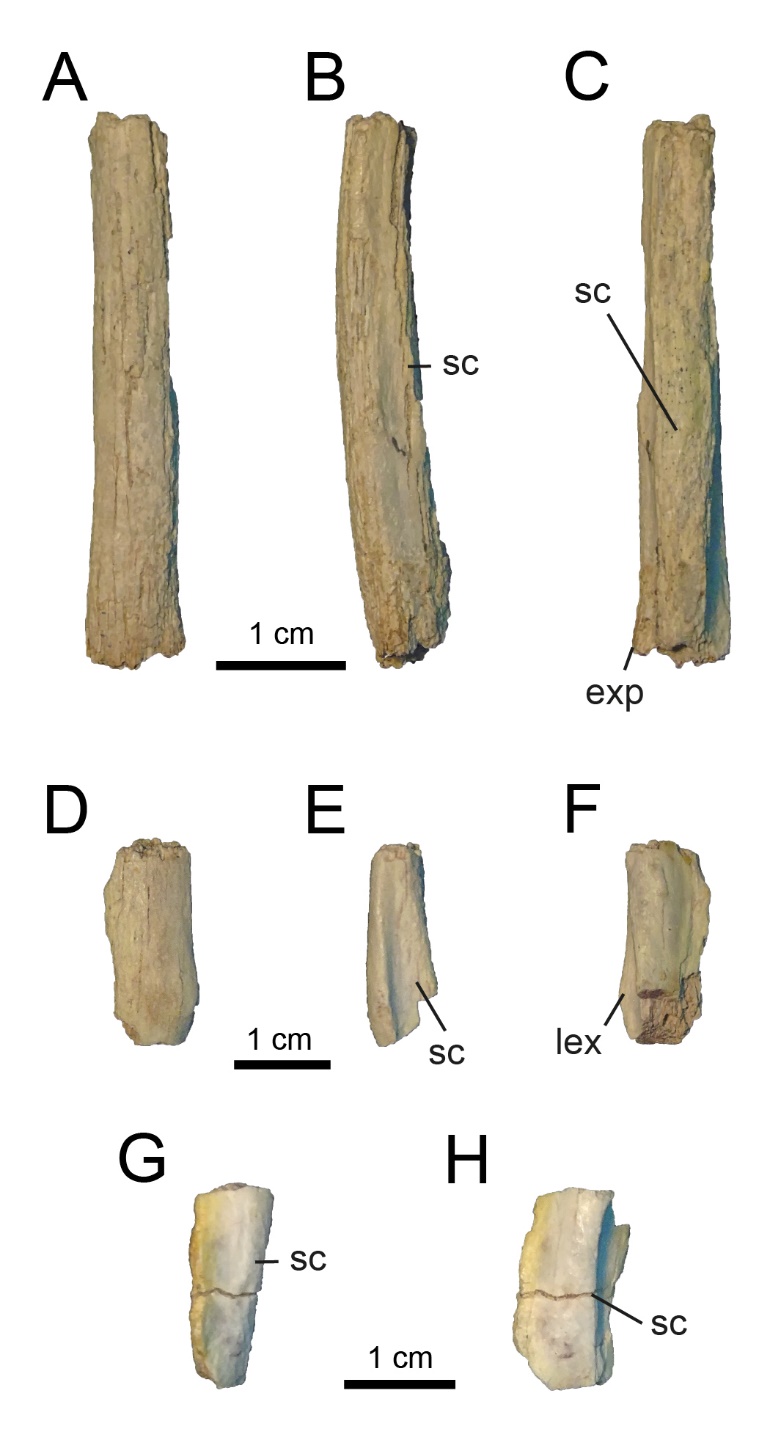


**FIGURE S12.** Metatarsal III of MPCN-PV 738. MPCN-PV 738.25 in cranial (A), lateral (B), and caudal (C) views; MPCN-PV 738.33 in cranial (D), medial (E), and caudal (F) views; MPCN-PV 738.34 in medial (G) and caudal view (H). Abbreviations: exp, lateral expansion; sc, shaft constriction. Scale bar equal to 1 cm.


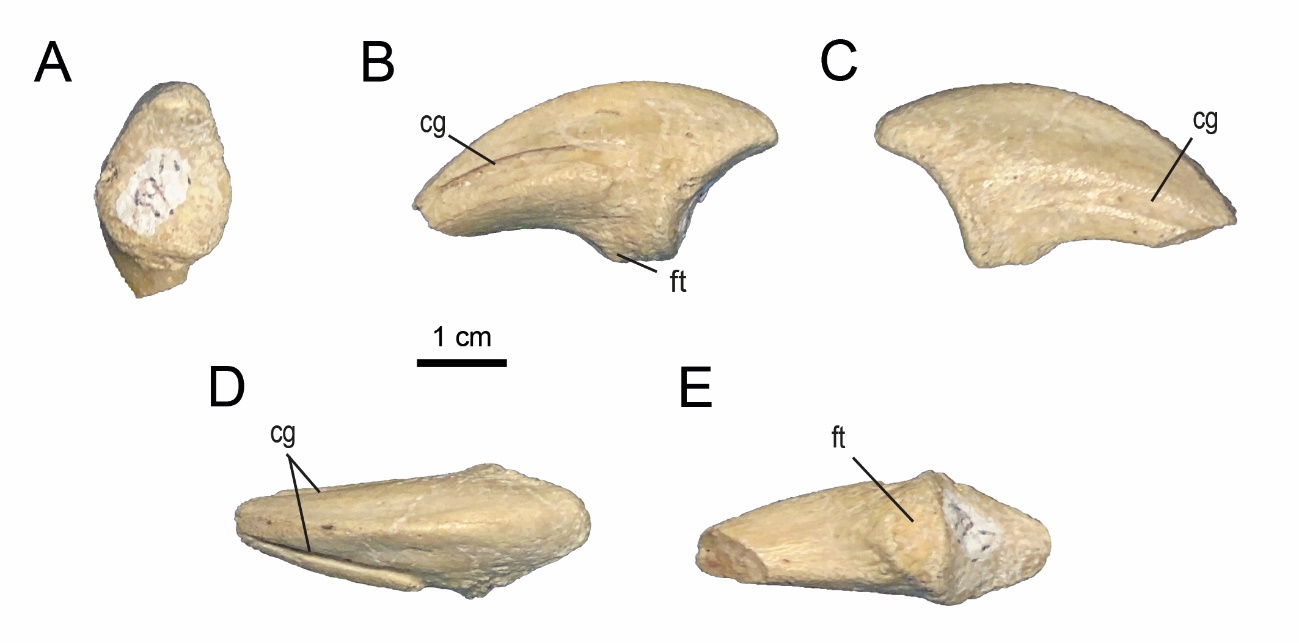


**FIGURE S13.** Ungueal phalanx of pedal digit II of MPCN-PV 738.46. A, cranial view; B, lateral view; C, medial view; D, dorsal view; E, ventral view. Abbreviations: cg, collateral groove; ft, flexor tubercle. Scale bar equal to 1 cm.

**
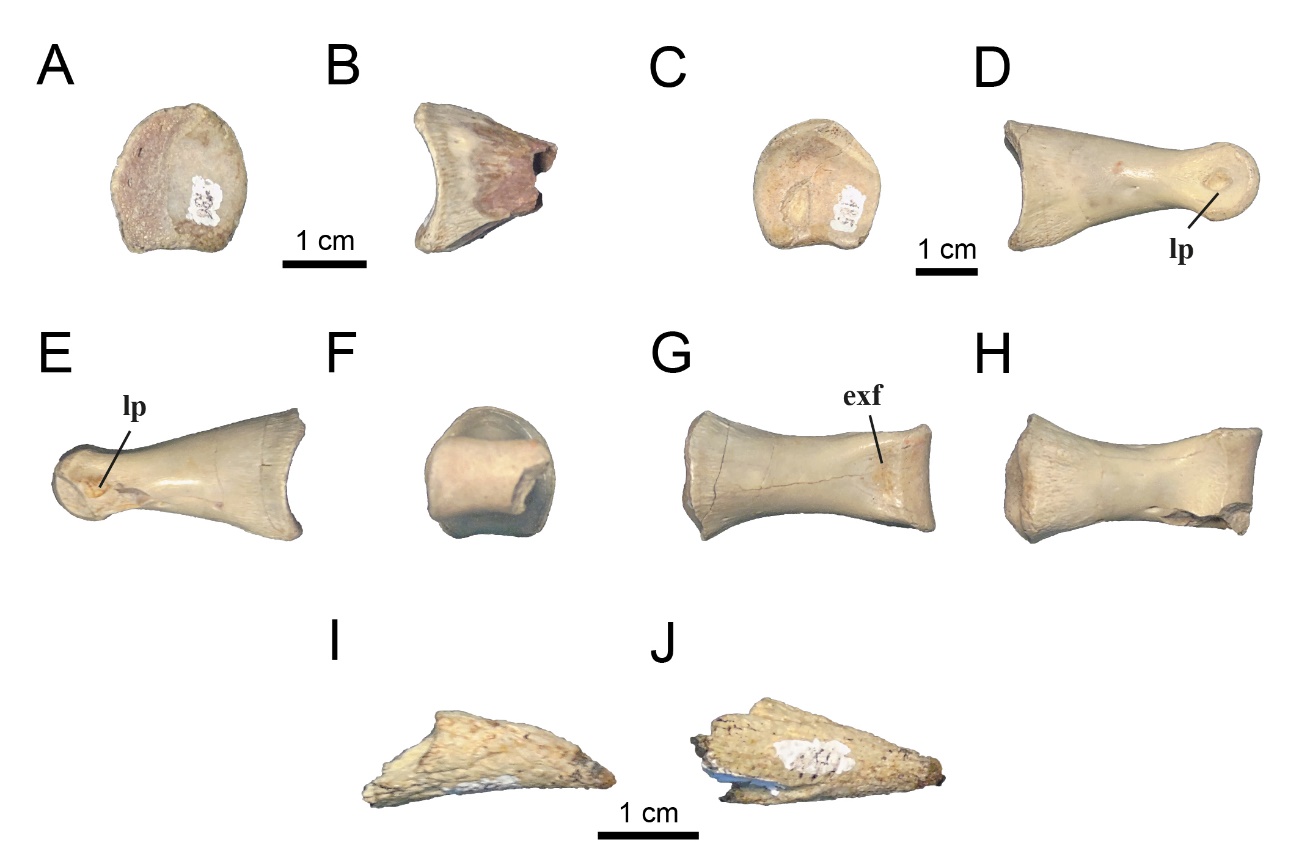
**

**FIGURE S14.** Pedal phalanges of digit III of MPCN-PV 738. A, MPCN-PV 738.22 right phalanx III-1 in cranial and lateral view (B); C, MPCN-PV 738.18 left pedal phalanx III-2 in cranial, medial (D), lateral (E), caudal (F), dorsal (G), and ventral views (H); I, MPCN-PV 738.44 ungueal phalanx III-4 in lateral or medial view, and dorsal view (J). Abbreviations: lp, ligament pit; exf, extensor fossa. Scale bar equal to 1 cm.


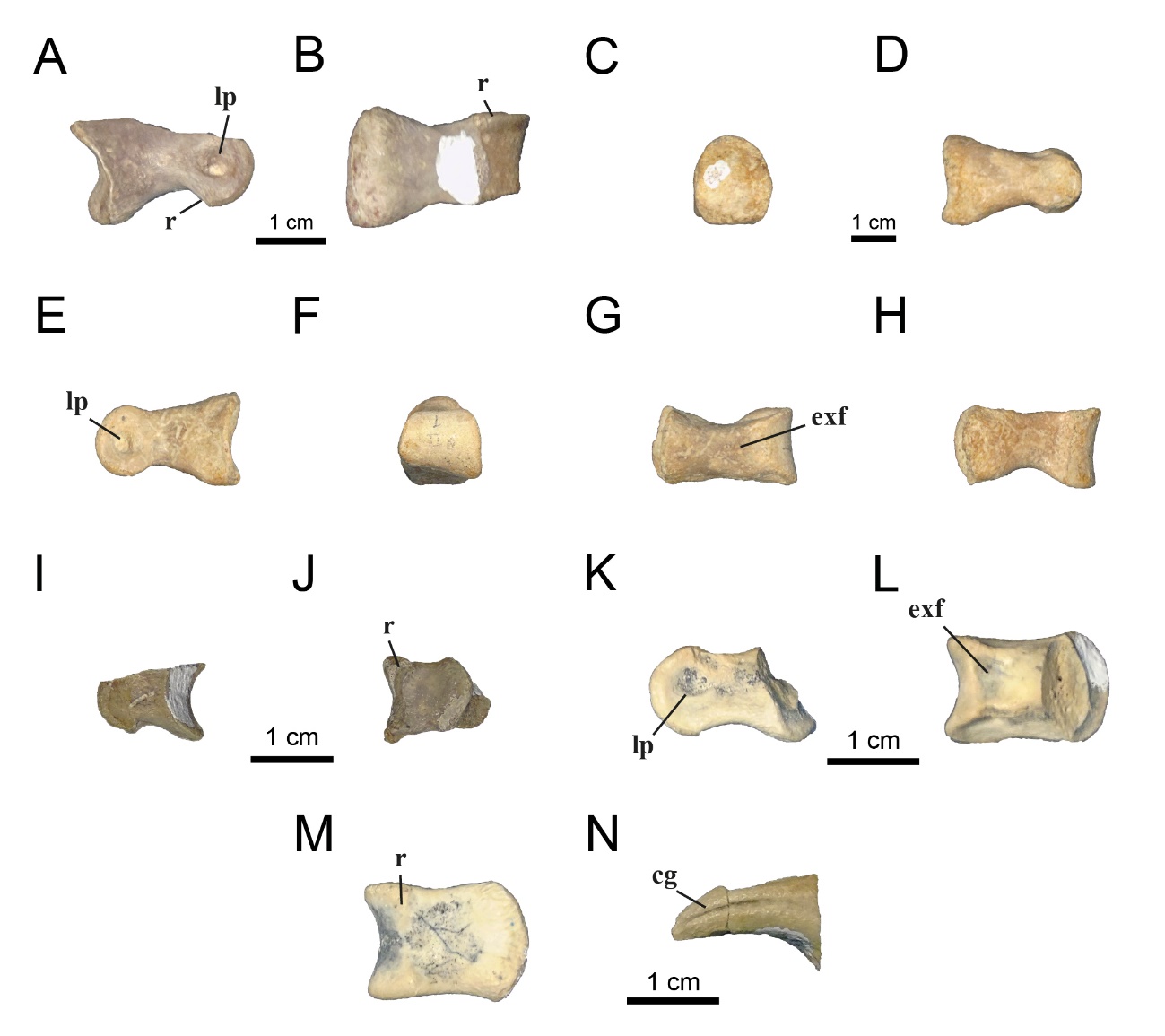


**FIGURE S15.** Pedal phalanges of digit IV of MPCN-PV 738. MPCN-PV 738.23 left pedal phalanx IV-2 in medial (A) and ventral (B) view; MPCN-PV 738.19 right pedal phalanx IV-3 in cranial (C), lateral (D), medial (E), caudal (F), dorsal (G), and ventral (H) views; MPCN-PV 738.20 left pedal phalanx IV-4 in lateral (I) and ventral (J) views; MPCN-PV 738.21 right pedal phalanx IV-4 in medial (K), dorsal (L), and ventral (M) views; MPCN-PV 738.45 ungueal phalanx IV-5 indeterminate in lateral or medial view (N). Abbreviations: cg, collateral groove; r, ridge; exf, extensor fossa; lp, ligament pits. Scale bar equal to 1 cm.

**C. DATA MATRIX**

#NEXUS

begin data;

dimensions ntax=122 nchar=647;

format missing=? symbols="0~7";

matrix

Herrerasaurus_ischigualastensis ??000000?00??100----???0010-00000000000????0?00000001?2010000000000-0--01100--00100000??-?00?0-00?00??00?1????01???11??000?0?0000?0?????0???000????01?1?0?0?0-000????0???????????0???1?000-0?0011???????010??000??????????1010?000?010000000???0?000??00-0000100?0????0010?001000?000000000000000000000100?0?01?1??0?000?00???200000000?000?0000?????????00??????????0??0-???00?1000010?001110?10000100??0100?0?00001?0??01000-0?012000000011000?11100000???0000?0-0??0????00-000001001????00??01010???00000?0101????00-1?1?0-??010000?2000000000000000?0000----0?0??100????0010000000?000?0-0?00?0000?000?01000-10000000--00?000001001100000000000000000?010?0000?????

Dilophosaurus_wetherilli ??001020?00??0?0----1?????0-010?0010011[01]0[01]1??0?0?1001?2010001000010?0--1111???0010?0?0???00010-01?0????111????000?011????????0000?0???0?1???000???1?1?0?0?0?10000??101?????0???????1?1?1?0-???0???0-10?001010?01??????????1010?000?1000000000??1?10???00-0110110?1101?0011?0011?0??0?000110000200???10?10000????1100?030?00???0?0000020?000?01000?0???10?0???????????0??2000000100???00?0??100?100001000?0100?0??010?01?00?000-1?002010000011000?1?11000?????11000-???0????1?00?0001101????100?00-00???100?00?100????10-????0-??11000001000000000?0000??00110?000?0??100????001000000??0?0?10??0??0000?0?2001000-??????????????????????????????????????????????????????

Coelophysis_bauri ??000011??0??000---????0?00-0101001?1?10???0?00001001????0000000000-0--11100--00100??0??-000?0-01?00??0??0?????0???????0?????0000?0???001?????0?????1???0?0?1000?????????????????0?001?000-0?0011???10?100-??000??????????1010?010?020000000?????000??00-1??0100?11000?02??0010?0?100??00[01]00002001??000????0????00?0?03??00???100000020?000100?01?0??????00??????????0???????001100?000?0?01?0?10000100??0100????000??1?000000-1?0000?000?0?100??111100?0???001000-0000????100000001101???000?000-00???0001101100????10-1?0?0-??1100?0??0?0000000?00101?0?110???0??0?001????001000000??0???10???0?0?0????1001000-100010????00?0000?00011000000000?0000000?00?00?00?????

Tawa_hallae ??0001201000??00----01001?11000?00000?1????0?000?0011?200?001000010-0--12100--?1100??0???000?0-00?0?0-??11010000??0????????????0?020?00?1???000???????000??1??0??????1?????????????10??000-00000-00-100?00-??0????0????????00??00002000?0000?0000?000100-0???????1100?00??00?1?????0???0?????0?????????1????1?????0????????0???????0??00????0?0?00????????????????????????????1?10000100?00100110000?????????????0??1????000???10??2?0000?01110001110?000000000000-0???0--?0?00??00000100?000?0?110????????10?????????????????????001??2000000??100????????????????????????0001000000000?010-?00?00???00?1?????????????????????????????????????????????????????????????

Carnotaurus_sastrei ??10000000001000---0??00?010000100010001???1?1001110??200000000?000-10101000--?10-1100000001?1101100101000010000??00-???0????1000??2??0??????0?00????00?1001??0?0????????????????1?101100??00?01000-100?0100-0??0?11101000101000??01000000?0?0000000???0-000010101101?10?00??11102?1011100?000100?0010?1000010011?110040??11?0??????00????0?????0000???0?????0???00--0000-000?01000011010?0221?0110?2000000-000111000----????????1?0???01????????????????????11000-0000??????00?00??00??0?000000100????1000000100?000?0-00000-??000?10000?0111011??011?00?110?0????????????????????????????????????????????????????????????????????????????????????????????????????????

Ceratosaurus_nasicornis ??10100010001100---00?00?0100000001000010001111010101101-1000000001110?11100--0010010000000010-011101010010100010?00-0?00?0??1000?02??0?1???000???0???1?1???10?00???0????????????1?????000-???0???0-10?00100-0?0??????100?100??000?11000?0000??0?0?0??00-0000111?11011000000011??0?0011101?000100?00???10000000110110040?01?0?1?000?01000?0?01000010??10000??????????00020000?0100?0?1000?????????????????10001100101????0100??1000001000101110??1??????2????1100??0000????1?00000010010000000000-000101000100100000010-00000-0?11000000000011011000111011110?0?0000?1000???001000011?????????????00?0???????0?0????0????????????0?000?????000????101??01?0????????????

Limusaurus_inextricabilis ??1000101001110101?00?000?11?0?1?01?000?---00000?000??01-?000000000-10012100--00120??0???00000-00?1????0000?0?020?00-?????01?000?????????????????????????????????????????????????0?20-?00??01?1110-?0-1000-??0?10?0??0????11------1-----------------------000??0?1101?1?2?0000-??01?0???0??????????????????00???1????0??????????10000?000???020000000-0??1101000??0-10002000000110000100001?10011011?000?00000?000000----?0000--010001000101[123]10-00011--02???000000-000110001000?1001001001000000100???0?0000?00-??000?0?00?00?1???00?010?0?0?????00011????????????0??10??????1???????001?0???00??0??00???2?00000-??????????????????????????????????????????????????????

Majungasaurus_crenatissimus ??10100010001000--??100000100001000100011100?1011010112010000000010-101?1010--010-1100000011?11011111011000100010?00-0000001?1000012??001?00000001??1?????0???0?0?????0-?????????0?10110???0?00?????10?0?100-0100001001000101000000110000000000000000100-0000111010-1?10000?011?02000111010000100100101?0000001010110030???1?001100002000000110000000?100100??????????000-00000???000?0?00022100100120000??????????0?????????????1?0???01???11???????????????010010000010001000?0001001000000100????????????0???0??????-??0???????????000000??00100011101101010000111100010000100010??0??0???0000000?0??0?0000011??????????????????????????????????????????????????????

Cryolophosaurus_ellioti ???????????????????????????????1??1?1??000??0??0????0?2?????0?00001011??1100--001000?????00000-01?00??10?10??00????10?0?0???????0???????????????0???10101?????000??????????0-????0???????????????????????0-0-110??0???????????????0[12]??0?0000?????????????????????10-1[01]????00?????0?????0?????0?00?00???10010??0?1?10?0????????00???0????????00?0?0????0?00??????????????????????????????????????0?????????10000????????????????????????????????????????????????00?????????????0?0??1??10?????000????????00????????00????10??????01001?010??011?0??0000??????????0??????????001100000???????????????????????????????????????????????????????????????????????????????????

Monolophosaurus_jiangi ??001000100101?10000?00?01110001000?0000??001111000100201100000001111??11100--0010000000-000110011000-0011000001??010????????0000?????0?1???00??????1010100?100000-??????????????0?0011110-?0000-00-100?0100-1????0100211?1010000002000?00000000000?010100??1101?10-1?100?01011?00010?100101001000000011?000000?10010030?00?0?????0?02????0?????0????????????????????????????????????????????????????????????????????????????????????????????????????????????0100100000100100?0?01000010000000000-??0101000100100?00110-100?0-?????????????????????????????????????????????????????????????????????????????????????????????????????????????????????????????????????????

Piatnitzkysaurus_floresi ?????0?????????1??????????1000????110?00100?1??????????????????????????????????????0???????????????0????????????????????????????0?????0?1???0?????10??1?1?0?10?0010?010-?????????00????0?10????????????0??????????????????????????0?1?01?00?0??0?0????0100111110?10-1010?1?0011????10??001?10010???????11000????1?00??30??0???0???0?02????0??????????????????????????0??21000?0?0????00?0??1?0?10?00???0??100????0???????????????????????????????????????????0100??0????????????010000??????????1?00????0000?010?????11-1???0-??110010?0100010?0100??0??10111???0?0????0??????????????????????????01?0?????????????????????????????????????????????????????????????????

Baryonyx_walkeri ??0101201001110??????10?0?11?0?000??00100000101????0??2??????0??0?101??10100--0010011???10?000??1????11???????020?010??????????0000000??1?10010???0?10101000110000-0?10-00-10??0?????11110-?0100-00-10010??1111000???0101?103000000?20000010102101000100-1101111?10-1010?001011?00010??01121001100??000110000??0100100?????????0??0002???????????00???01??0?101010???0000-???00?00?0?10000?2010?02010001012100110101??????????????????????????000????0100?????1?0??????????1000???0000100????00?0-????0????0?0100000?100?0000-0?010110??????1000100??????????????0?0100000?00?????????????????????????????????????1???????????????????????????????000??0???10?00000????

Eustreptospondylus_oxiensis ??0??020100?1100----00000?1100??0001001000?0???????1??????????000?1-11011000--0011011?00000010-010?0????010?00000?00-????????0??0?????0?1?00010??01010001?0111?100-??10-00-10000??0??1?110-?0????0101001?????????????0?????010000002000?0000000101000100-0110111010-11100101011?00?10??00100002000??000110000??01000?030?00?0?0?[01]00002???00?00????????????????????????????????1?00?0?100?0?200010001?0000?????????????????????????????????????????????????????10???0??0???01000?000000110000000010000001010000100010?0?01?000-010100100000001000100010000011100000001100000000100011??00??????00000100000?001000-??????????????????????????????????????????????????????

Afrovenator_abakensis ?????0???????1?101001??0??11010100110?00000?1???????0?201100??0?001010011100--00?????????00010-01??0????010000001?010????????0?0??????????????????????????????????????????????????????????????????????????????????????????????????02000100000???????0100-0110110?10-1?0001?1?11???010??0?[01]?10?10???????1?00???????0????????????0???0??0?0?0?100???????11?00??????????????????????????????????0??0?01???????00?????????1???011??1??00???????12100?2???010000000100100??0????00?00010000100?0000000-0?0?01???000100010011010000-?0?0?1100010001000100????0?0??????001????00?0000100?111??1?0?110????01?????2???0?????????????????????????????????????????????????????????

Allosaurus_fragilis ??0010[01]01001010?0000?0000?1000010?110?0100100001?1?011?1-000000000101???[01]100--?111010000-01000-010?0???01?0100010101120?00010??0001001001?00?000010?00101001100100-1?10-?0-0-?000000010000-?0000-00-10000110-11?000100101010200000021001000000000000010100000111010-1000000101100001001011010000100000010000001010010030?00000000000020000001001000???1?0100??????100000210000101000000000010000000010000000000000001010000111010000010001012100021110000000001001010001000100000100001000000000101000?1010000100000011010000-00010110001100110010001000101110000?00010000000110001110?1?00110000001010?02001000-10001000--000100001001100001001010000000?1100000000000

Neovenator_salerii ??00?01010000101000010000?1100010011000101000101110????????????????????????????????????????????????0???????????????????????1?????????????????????????????????????????????????????????1?100-?000??00-??00??????????????????102100000?100100000000010?010100001111011111100001011?00010??00200000010??001101101??1100100?0?10???????000100000100010?10??100100?????????0002100101?10?0?1010????????????????????????????????????????????????????????????????????0110100??010001000??000001000??110010100101010000100000011000000-00010120001100110010101010111110010001101001????100?????01??????000?01?10002001000-??????????????????????????????????????????????????????

Sinraptor_dongi ??001000000000?10000000?010-000110110000?0000001000000200000000100101??11210--?110011000-010010011000-00010000010?011300000100001000010?1?00000??10?00101000100100-??10-?0-0-?0?00?1011000-?0000-010100?01110010000??010101010000002100?00000000000?010100110111010-10100001011?001100101101001010001001100000101?010030?0000?????0002?00?0??1?1001???1??1??1?0000???0????????1010?00100?0???????0??????????????????????????????0????1100101210?????1000000100100101001???000?0??1000010000000001100??11010000100000011010000-00010?1000110011001000101010111?000000?00?0000001000011000?00110000001010?0100?000-??????0?????0???0?????????0?????-000??01?11??0?00?00??

Acrocanthosaurus_atokensis ???010100?0??101?0??10?0?10-?0?1?0110001001000?0?0?0112011??0?00001010??1110--?11?0??0???01101111?00??10110??01001?1120?0??1?0??0?????0?1?0?0?????1??0101?0?10000????10-?????????0?????200-?0000-?10???0?101011???0??0?0??101??000021001?00?0?0??1?0???1000?1??1?111110??001?11??0?10????2?1???01??????10?1?0??0??01?0?1??????????0?02??????10?1?0????1?010??????????0???1000?10100?010?0?0100000??10????02??00?101??0?0??011101?000?1000100220002??100?0???00???10??????????????10??????????10?1010??0?0[01]0??0????00?11?10?00???01012?1001?011?0??00100?101?????0?0?????0??0??100????001?000-00??0?1010??2?01000-??????????????????????????????????????????????????????

Giganotosaurus_carolinii ???0?00????????0????10????10???1?001000100??01?1?0????2???????????1010??1110--??1?0??????01101111?01??1???????1?0??10[12]??????????1????????????0????0???101????????????1?????0-????1?????201-?0?0???10???0???10?11????????????1???000200?0?10?0?0??0?0???110001??1?111110??[01]?1?11??0?10??0?2?0???0???????10010????1?01??????0???0?000?01??????000???0???1000???????????0??????????10??010?0????????????????????????????????????????????????????????????????????0???10000?110?1??0?0?000?11?????10?1010??010100?0???????11?1???????01012?100??01??0?????0??10111???0?0??1?00????1?????????????????????????????????????????????????????????????????????????????????????????

Ornitholestes_hermanni ??00?0000?01?00101011100??10000?01101000000000?0?001110001100000010-10011100--00101?0100?10000-000201000?001000000011[23]0????100000???0?0?????0?01001111101?100-????-????????????????0010?10-?0000-00-100?00-0-11100011010001012011102101-001000000?010100-????????10-1?101200011??111100?11?1001??0001001??100001010110?0???10?0?10?0020000?10?0??????????1?1?????????????????????????????0?10?11000010001?0??0?0?0?0?????0??????????????0?????????????????????1?0101??0100000?0?0010011000000?10????010?01000010?000001?10000-0????????????10?0?10?????????????????00??0?????????????001??????0?100?0?0?0?0010?0-?0?1??00--?0?0000??????00????????00???0???1??0????????

Zuolong_salleei ??00??00?00?010101001?0?0?0-0000001110000000??????00???????0??010?0-0--1?10111011????00??00000-0?1100-00?100000001011300??0????0??????????????????????????????????????????????????????????????????????????????????????????10100001020?0000100???????010100???????10-0?101100011??0?100???00?001???????????????????????40?001?000??000000??0?00????????????????????????????????1?10???1???0?10001???0100???????00?000????????????????????????????????????0???00100??????????00?0???0???100?00000011?0010112?00???????????????????1?011000100001001000001010111?0???011??000????????????010????100010101000????0?????????????????????????????????????0???????????????????

Albertosaurus_sarcophagus ??1????0000011??0????10?1???0??0??0100000101110?11??002011001100011111011201??000-12000010111100112010101?00101010010300000100000000000?1?00010?01??1000?00110000100?10-?00???0000?001??00-?0?00-00-10000101011?011100???010??00?10?00??0?0000000?0001010???011?0???????0000011?00??????0?0?00?????????1??000??0?00?0041?0?00???10??0??00??0??0?00?0????0??0000001??000020000???00?0??0000?11?001????????00??0?00??01000000010-?0???????????320?0??-?0000????????10101?100000000?20???1000000?0010??0001???00010000000102000101?1101100110000100100000001?1110011001?1000100?2000110?001?0011000001111000?0?1000-??????????????????????????????????????????????????0000

Daspletosaurus_torosus ??100020100111110100??00?10-00000101?000???11101??????2??0001100011111011101100011?01001?000?0-011201010110?1?101001030???01?0000????????????0?00????????????????100?10-?0-10?0000???1?000-0000??00-1000010101????????211?10100001020000000000000000010100??????????????0???0????0???????????????????????????????????0????????????????????????????????1?111?????????????2??????????????????????01?????????????????????????????????????????????0????-????????0????????????????????20???????????????????????????????????????????????01?0002???1?0?10?????01?111???2??????????????????????1????????????1????????0?0-??????????????????????????????????????????????????????

Dilong_paradoxus 0-100020000110?101001100?10-00000011100????010210100012011001100000-11011100--0010001100000000-001200-0001?00010??011??0?????0000010010?1?0000000?????1?1??1??0001??0????????????0?0-11000-00000-00-0-0000-101111111101010101000010200000000000100000100-0???????10-1?100000010??0010???0?01001?????????????????1????0??????????????0?0??????2?000?0??1??????????????0????????1?10?0000?01?1???10001????????????????????????1??1??10?1100100220??21????00000??1?0??1??010000??0??20000100?0?0???11??????????????0??????0??0????11?01?0001?00??0?10??0?001?111??010???10???0002000111??01???????0?00?0??0?2???000-??????????????????????????????????????????????????0000

Gorgosaurus_libratus ??101020100011?101001100110-000000010000???1110?11101?001100110001111?11110????0??1210000001?0-00?20101011??10?0?????3?0000100000000??0?1?0000000?????????????000????????????????0?00??000-00000?00-1000010101110?0??0????10100001020000000000000000000?????????0???????0?0??????00??110??0??0000000?0?????0000?1????0???????????????????????????000??????????????10??????????1??????0?????????????????????????????????????????????????02???32????????????????100101011100????0?12100?100?000?00101?0001???00?10010?0010????????????????????????????00???????????0?0?10???????????????01000110??????1????????0?0-??????????????????????????????????????????????????0???

Guanlong_wucaii ??10100000011111010011001111000000111000010010110001012011000100000-11011100--0010001000-00000-0012010001101101200011300?10??000000000011000000001101000100011000100010-1001000000?001?000-00000-010100?00-10111??1??0????101000010200[01]1001000?000000100-1??????010-10101000010?1001000010010010000000010010001110010030?0010?00100000000?0??2000000?????11??????????00020000?1?10000000?0110011000010001110001000001010000010-1001001100100210002121000????00100101010100000000021001100100001010110001000000100000000-100010011?01100011001100100000101111100?2000?1?00100011001111001?00110000001010002101000-10001?????0?000?0110011000010?001?0?0001?110???000????

Kileskus_aristotocus ??10??00?00?0?11000011000?0-00??011110000100??????01????????????????????????????????????????????????????????????????????????????????????????????????????????????????????????????????????????????????????01?10?01???????????011000102100000000???????010??0????????????????????????????????????????????????????????????????????????????????????????????????????????????????????????????????????????????????????????????????????????000????????????????????????????????????????????????????????????????????????????????????????????????????????????????????????????????????????????????????00??????001?1?????????????????????????????????????????????????????????????????

Proceratosaurus_bradleyi ??10??000000011101001100111000?001110000???0??1???010?001100010000?????01?0????01???????????00-?0??????0110?0000???112?0??0??0?00011010?1??011????????????????0??????????????????0?001?000-00001000-100000-10111100??0???01010000102101-0010000000010100-0?????????????????????????????????????????????????????????????????????????????????????????????????????????????????????????????????????????????????????????????????????????????????????????????????????????????????????????????????????????????????????????????????????????????????????????????????????????????????????????????????????????????????????????????????????????????????????????????????????????????

Juratyrant_langhami ?????????????????????????????????????????????????????????????????????????????????????????????????????????????????????????????????????????????????????????????????????????????????????????????????????????????????????????????????????????????????????????????????10-1?100?00011?00???0?0010000100000??1?0???0010?00??030?001000???000????00???????0???1??????????????????????????????????????????????????????????????????????????????????????????????????????0100110??1100?0100??21001100?00000011?1000?00001010001000001?0?101????????0????11001010101010110001??0????????????????????????????????????????????????????????????????????????????????????????????????????

Tanycolagreus_topwilsoni ??1??000?00?00????????????????????????????1?0000???0???????0??00000-101122011000100??0???00?11001?????????????0000010?????????????????????????????????????????????????????????????????????????????????????????1000????101??01??0?10??0??000?0????????????011???1??0-??1????????????????0?0?000100??????100110??0?001?0?0??????????00?0???????20?00????1??????????????000200000111000000000110111000110001110000000101?11000010-10000011000002200020?100000000???????????????????????????????????11110101???00??????????????????1010110001100010010000010101110001000110001000200011110010001000000010?0002001000-1101???1000?011001100110000?00?0100?000??110?0000?????

Tarbosaurus_bataar ??0???00000011010000?100110-000000??0000????110010100120110011000?111101???100001??01000-0?1010?112?111011001010??01031000010000?000??0?1?10010001?000001001??0000-??????0-?????0??0011?00-?0000-00-100001?101110111001010101000010200??0?0000000?00010??????????????????????????????????????0???????????????????????0?????????????????????????????????????????????????????????0????0??????????0?????????????????????????????0-??1?0???02???320???????????????????????????????????????????????????????????????????????????????????????????????????????????????????????????????????????01?????1??????1??????000?0-??????????????????????????????????????????????????0???

Tyrannosaurus_rex ??10?0000000110101011100110-00000001000001011101101000201100110001111101110110?111101000-011?1001120111011001010100103?000010000001001001?10?0000100000010010-0?010?010-?0-100000000011000-00000-00-10000101011?011100101010100001020000000000000000010100000111010-11?00000011?0001011001000000000000010000000010010031?00000?0100000100000020000000-100110??????10100020000010100000000001100010012??00010001001001?0?000010-101000------03200021--00000?00010010101110000000?12000110000001001011000101000010000000102100101011012001210010001000100010111?0010?0??0?0100020001101001?0011100001-110002000000-??????????????????????????????????????????????????0000

Stokesosaurus_clevelandi ?????????????????????????????????????????????????????????????????????????????????????????????????????????????????????????????????????????????????????????????????????????????????????????????????????????????????????????????????????????????????????????????????????????????????????????????????????????????????????????????????????????????????????????????????????????????????????????????????????????????????????????????????????????????????????????????010010101110010100??21001100100010????????????????????????????????????????????????????????????????????????????????????????????????????????????????????????????????????????????????????????????????????????

Xiongguanlong_baimoensis ??10?000??0?1?1?0????100110-?0??011?000001?0102001??0?0011001100010-1?1??100--?0111??0?00001???0?120111011??1000000103?001?1?0000000???1100000???0??10001000110000-???????????????????????????????????????????????????????101??01102100000000???????0100-0000111010-1?10000001000001001000000010?100100100110?10100100???????????????????????????????????????????????????????????????????????????????????????????????????????????????????????????????????????0100??1??1100?000001210??10000000??????????????????????????????????1100100011001100100????????????????????????????????????????????????????????????????????????????????????????????????????????????????????

Compsognathus_longipes ??00?011000????1010?1?00??1101000???0000???000000000??00001000000?0-0--111?0????1?1101???000?0-001000-0?0??????0???????????????0????????1????????????????010??0????????????????????0011000-?0?00-00-0-000100--0?000??1210?101000100210110010000000000100-0??0??1010-1?0?0?00010??0?1???0?10?0?200?00???????10??0010?103?????0??0000?0?000?01000010000-1?1000??????1??00020000?1110???000000?0??1???1?00??01???000000????0?01011???00?1000?1?22??12??10000011001??0-1000???0???0??0?0???????00?001111???00100001000100010100?0-?1??????000??1????1???00?0??????00?0??????0?0001000?10?001?0011000?00?0?0?02001000-??00??????0??00?0?100?1?01?1?0?1??0?0000?11??0000?0000

Huaxiagnathus_orientalis ??0???000001?0?10100??00??11000???11??0????0000?1000?????110?0000?0-0--?1?0????1???1?????????0-?0??0????0??????????????????????0?????????????????????????????????????????????????0?001?000-00?00-00-0-0???????????????????10??0??00?0?1-00100000000?0100-0??????0????????????11??10????0??0?0?100??????????10???01??10????????????0????00?0?120?1000??1?0000??????10000020000?1110?00000000100???????????00????00?00?0100?001???00?0?10?0?1?22??12111?000000?01?00-10001010???0??0?0??02??000?1011?????????000100000001010000-?1??????0?????????????0????????????0???0????00?2000110?001?0011?0??00?0???020??000-??????????????????????????????????????????????????????

Sinosauropteryx_prima 000000000001?001??0?0?????0-0000????0?0????0000???????????100????00-0--?1100????11????????0??0-00??????00??00012?0?10??????????0??????????????????????0???????0??????????????????0?001?000-00?0??0??0-0?0?????????????????10100?1002101-0?1000000?000100-0??????010-1?1?0?10?10??11????0?20???100?00???????1000?01???03?????????000?0?000??1120?10000-1?1110?????????00020000?1110000100001200?00?00?????01?0??00000?1100?0111110?1??11?010?22??121110100010001000-1000???0???0??0?001020?000?1011?????1???000100000011?100?0-?1??01?0001??0??0?1????0?01??10?00?0???0??0?00?20001101001?001100??00?0??0020??000-01001?????00?1000???011000??0000100?0000?010?0000?????

Nqwebasaurus_thwazi ????????2??????1000??1011?0-00??00?1???----0??????????????????????0-10?1?2011101100201001??0????010?0-0???????0?00?10????????????????????????????010?00?101???0000-???????????????????????????????????????????????????????????????03?11-21211???????--00-0???????10-1??01200?0-?11111???100100??????????????????????????????????????????????????0???????0????????????1002100001?10000000??0100?100?0100?10??0?00000010?00000010100100100010122001111001010110???????????????????????????????????1???0?0????00??????????????????1??0??1?????000001000001011111?0010?0010?0?0002001110?001?0011000000001000?0101010?0011?010001?1000?000??0?0?00??0?00?000??010?010??????

Anserimimus_planinychus ???????????????????????????????????????????????????????????????????????????????????????????????????????????????????????????????0??????????????????????????????0???????????????????????????????????????????????????????????1?????????????-?????????????????????????????????????????????????????????????????0????????????????????????????????????????????????0?????????111200000????0100000?012???????2???????????????10000?1010-01?1001001?00220??111110012210????10100?100?11?0???????1010000?0010????00???00?1000100010??000-???????????????????????????????????????????????????????001?1---?00?010110???0??1????0011?????0??0000?1001?000110?0000??000??00000?000????

Archaeornithomimus_asiaticus ??????????????????????????????????????????????????????????????????????????????????????????????????????????????????????????????????????????????????????????????????????????????????????????????????????0?????????????????????????????????????????????????????????00----20220011000121100000000020000000010000000000010031?1010?0?1000020000000?00??0?????0????????????101210000111001?1000?00200100012000010000000000????001010-11?100110110022?011???00011210010?0-0000100010000100000100010??00101100011-01001010100010000010010100100011011000100000101111100010?0????100002000110100101---100010011000?0??1?1???????????????????????????????????????????????????0000

Beishanlong_grandis ????????????????????????????????????????????????????????????????????????????????????????????????????????????????????????????????????????????????????????????????????????????????????????????????????????????????????????????????????????????????????????????????????????????????????1????????????????????????????????????????????????????????????????????1???????????1112100001?00010000?0002????000100000000????000?????????????????????????????????000002?0?????????????????????????????????????????????????????1????????????10?0??00021?0110?10001010???1100010?0?1010100?1000110???1?001?????00??10????001011?0???????????000??????????0??????00????????0?01???0???

Gallimimus_bullatus ??0???00210000000101?101110-0?000???000-???00000000?11001?110?000?0-0--1???111011200010000?0?0-?000?0-001?010112??00-??01?010?10?0?110??1?0000100010?0001001??00011??110?0-100010??10-0??10?1010-00-0-0?01?10?1?1000-0???011------1---??-?-?-2---???---?????10??010-??202210010??12?????0?0?????????00?1??000??0??0?0040?1??0???100?0??00??0??0?01?0????0??0?????????011200000??10?1??0000?02?0?0???2??0000??0000??1?0??001010-?10????????0?2???0??2?00011010?1?010100?100011?0??0????1010000?0010??01001-?000100010001010000-110?????00?1001?0????00?00??????00???00?0?0100??0001???00101---100?01?1?010?0001010100110110000?000001001100000000000000000?010?000000000

Garudimimus_brevipes ??0000002?000001001?0101110-00000111000----0?0000000??01-0110000010-0--111011101120?0100-000?0-00?000-00?00?00?001?113?01?01001000?11?0????00010001?000010?01100?11?????????0???00???-0001001?10-00-000?00?1001?1000-0?1??11------1----------2------------000?-1?????????????0-???2???000001001000001001???000000001?040?10100?0??0000???00?0??0??0??????????????????????????????????????????????????????????????????????????????????????????????????????????010011100010011100?10010010010001001001000101020??????????????????10101100011001000110010001011100000?0100?010002000110?00100011100000?1101020000011??????????????????????????????????????????????????????

Harpymimus_okladnikovi ??00000021000?010?0??1011?0-000?0?11100----000000000????????0???????0--1110111?112010?00-000?0-00?000-00100100?????????????????0?????????????????????????????????????????????????0?0010001001011000-0000010100??0000000-0011------1------?--11?0??0?--?---?????1010-???0??0000-??02?0???0001?02??000?00????00??110?11040?1?10???1000020000?0000001????0?0110??????????11??????1?10?1?100?0?12?0?000120000010000000011?10000010-1001001100110220002111000111100100101001????11?0??0??00100?000000????010????00???????????????????0101?00???0?????????????????????10????????0001000110?00101---100000?0?000?000101110011?0???0??000001001100000?000000?0000?010?0100?0???

Ornithomimus_edmontonicus ??0?????210000???11???01????0?020?????????????00???011?0111100000?0-0--????1111112?0010000???0-?00?????010010101??011??????????0?0?11???1?00?????0???000?0?0??0?0???1110?0-10?010??10-0??10?1010-00-00??01?10?1?111??0???011------1?-???-?-??2??????????????10-?010-?1??22100???102?????00??00???0??1??1??0?0??0??0?0040?1?00???100?0??0???0??0?01?00-??0??0?????????11121000???00?10?0000?02?0?????2?????0??0000??1?0?0001??0-?1?????????0?22??0??2?00012210???010100?10001100???????1010000?0010??010????00010??10001010000-110?????00?1001?0?10?0???0??????00???0??0?0100??0001???001?????100?01?1?0?0?0?01000100110110000000000100110001000100000000??010?0000??000

Pelecanimimus_polyodon ??00000021000101000111011?0-00010011100-???00000?00000001111?000??0-0--1120111011202?1?0-000?0-00???????????????0?010?00???1??10????????1?00?01????????????1???01????1?????10????0??00?001000?1??00-0-00010100??1?0??0????1030001103201-102110201010--10-0???101010-1?????00010?1???1?0000?1??????00?????????????????0??????????????????????????00?110??????000001???1????????1?10?10000?0??200?0001?000?0100?0000011??00?1010-01?10?110110?22000112100011210?????????????????????????????????????????????????????????????0??????????????????????????????????????????????????????????????????????????????????????1?0110????0??0000?10011000?00010000?000??110??100?????

Shenzhousaurus_orientalis ??00000021000001000??1011?0-000?0011000?---0000010001?????1???????0-0--1120111?1????0?00?0?0?????00?0-0???0?01????0????0???1???0??????????????????????????????0??????????????????0?001?001000010-00-0-000??1??1?0????0?????1------1---1-----11001001--00-0?????????????????0???????????0?00???200?000??????00???0001?041?1??0??????000?0000?0??0???0??0?01???????????????????????????????????????????????????????????????????????????01??10?220???1210001011001000-100010101000???1000100?00010010010100?1?010100000000010000-???1????001100??00100??????????????????????????????????????????????????????????????1?0?1?????0???000???011000?0001000??000??010?0?00?????

Struthiomimus_altus ??0???0021000??1?10?110?110-0000011?000----?0000100?11???1110?00010-0--11?01111?120001000100?0-?000?0-0010010112?1011??01?0???1??0????0?1000?????0??0000??0???0?010??110?0-100010?000-0001001011000-00??01010?1?1010-0???011--?-??1----------2------------??10-?010-?????2100???002?????000??????0?????1??000??0??0?0040?1?00???100?0??00??0??1?01?0????0?10?????????111210000??10010?000000200100012000001000000001?0?0001010-0101011101100220001?2?000112100100101???10001100?10????1010000000100100001-0000100010001010000-110?01?00011001?0010?0?000???1100010?0?10?0100?200011??00101---100?01011010?0001010??????????????????????????????????????????????????0000

Alxasaurus_elesitaiensis ???????????????????????????????????????0?????????????????????????????????????????????????????????????????????????????????????????????????????????????????????????????????????????????1?00110101??10-101?0?????????????????????????????00110?102110001010?0?????????????????0???????????0?00?001001??0011??000??01001004??1??0??????????01??00?2?01??????0000??????????0????????????0?????0?1000100111001101????00??0?100010000-00?0??110010122??02???00100100???110100?110101?0???????0-??000?20???????1?????011110?1?1?220?0-???????111?00???0??0?10???????????????1?????????????????00?010-?00?00???0?0?001?0???0????????????100????1??1????????00?000??11??0?00?????

Beipiaosaurus_inexpectus ??????????????????????????????????????????????????????????????????????????????????????????????????????????????????????????????????????????????????????????????0??????????????????0???1?00???1?1??10-101??100-?????????????11------0220001100132010001010-0???????????????????????1?????0????????0????????????????????0?????????????????????????????0???????????????001002000001?10?0?000?0?100??????????10????????0????00100?????0?0??????0?22?????1?00?0000?01011110011??????1????????????????????????0???????????????0???????1??????00???0?0?0??0????0?????010????????10?0??0??????001???11??????????????????0-1?001?????0??10?0110011100110010100?000??110?0?00?????

Erlikosaurus_andrewsi ??100000100110000?-0?101110-00001101000????0?00????11????1000001010-0--?1100--?110?10000010000-00100??0000010000?0??0???1012100001---01?0????00?00?1000000000-??00-????????10??000?1010?011?1011010-101000-0-11?0000-0100011-?----0220001?0013111?001010-???????????????????????????????????????????????????????????????????????????1??????????????????????????????????????????????????????10????????????????????????????????????????????????????????????????????????????????????????????????????????????????????????????????????????????????????????????????????????????????????????0????????0??0??????????0??????????????????????????????????????????????????????????

Falcarius_utahensis ??????????????????????0???????????010??001?0???????????????????????????????????????100000?0?00-00??0??????????1?10010???????????????1?00??00?00??1?01000?000??0?011?110-10011010??0??1?00100000??00-1010??????????????????????????0?20001[01]00101110001110-0???????1101?212100010?11211001120100200100110100101010?0010041?101000?1000120010011000010???101111?????010110020001?1?100001001?11001?00100001111001100?000110010010-101000110011022000211100100000010111100111010101?00000011010??11010010101010001121023001022000-010?01101011000000100000101111100010?11??010000100011010001001100??001000???001000-100010110001?1000110011000?100101000000??110000000????

Nothronychus_graffami ??????????????????????????????????????????????????????????????????????????????????????????????????????????????????????????????????????????????????????????????????????????????????????????????????????????????????????????????????????????????????????????????????????212?????????2??????20?00?001??111?????????10???04??10?0?0?2010??10?0??0?000100?????00???????10100?2????00???0?0100000110?1?1111001??100?100000?????????0-????0????????22????????000000001011120011??1???1?0??100???1100?10101000111--2?0121?0?11102?10???0??112111100??00?000?0010110???111??1101?0??0?2000?1??0001010-0000001?0110?0?0??0-11001????????10?01100?100???????01??000??010???00?0???

Segnosaurus_galboensis ??????????????????????????????????????????????????????????????????????????????????????????????????????????????????????????????????????????????????????????????1????????????????????0010?????1?1??1??101??0-0-?1?000??0???0????????0???001?0?10111?00101???????????????????????????2??????????????????????????????????????1??0????????????????????????????????????????2??????????10?????1???11?0?0??1100110??????????????????????????????????22???????00???????1011110011102010000101000-01000?201010????01000011110?1?102?101??0??????????????0?0??1??0???????11???11?????0??10000????001010-?00000??01102001??????????????????????????????????????????????????????????

Nothronychus_mckinleyi ?????????????????????????????????????????????????????????????????????????????????????????????????????????????????????????????????????????????????0?1??00?0?0??1??????1??1??10?10??0???????????????????????????????????????????????0???00110?101?1???1010-0???????110??2??010011?11?????112010??001???11100100??010??00????????????100????0????????????????????????10??????????1?0??0?110?0?1101?011110?1101000100??0????????????0?????????0?????????????0000????1????????????0???????????????12???????????????12110?111021100-00???????????0????????0??0??????11??111?1?10?????????????????0-?0????????????????????????????????0?????????0????????1????????????????0???

Avimimus_portentosus ??????????1?1?????????????????????????????????????????????11??1?????????????????????0000?????0-?01??????0?00?000???11????????????1?1?0??1?10?00??01??120?0?0??0?00-?110-????????0??00????11?????????11??10-0-?0?00???????111-??-??????????2??2????-?--??????110?111010012000110?11211100100100100000111100000?0000011060?1010?0?2?0000???00??????????????????????????200200000110010000100011001001010000100100000001?111????0-????1??????0??2???????????????0100??1???1000011101010001111?00?00100100011-00011110230010?0000-011011210110100000101000001001100011?11100010112000110111100?11000102-110102010100-??????????????????????????????????????????????????????

Caudipteryx_zhoui 100000??0?111?0?0000????????0?0?0?11??????????00???01?????000?01?0??????1?0101?10-?10000010??0-00??0???00????000??0????????????1??????????????????????????????0??????????????????0?201?001101111100-110?1??0-???0?0?????0?10???1101?--??-12??2????01--?0-???????0?0-????1??00??????????0?[01]0????00????????????????????04??1??0???2100??11????0??00?0???1?011?0?00??11000???????1?00?001?0?0?????1??10?00??0000?0??0001?110?0000-?0?1?111?010?22??021-100?000000100101001???0?1?10?0?000?????00?????????01???2011110??00102?0?0-?100?111111?0?????10??00?????????011???10????01200011?1001??0????01?1?0???1?0??000-10011?0??????00000100110010??0?1??0?000?0110??000?????

Chirostenotes_pergracilis ??1????????1?00??????1101??????????????-?????????????????????????????????????????????????????????????????????????????????????????1?0100???01?1???0???120?011??0?00-1?110??????1????10-0?01111111100-100-10-0-?0??00??0????????????1?--????-??2????-??????????????110??211??0?????12?????1????????0??10?1??1???????????52?10????0??1?0?????????????0??????????????????20?200000?????0??10????????????????????????????????????????00?0????0???2200???????10000??1000-100010000??1??010000-000000??12??00?????201111023001121000-01????0?0??????0?0?11??0?0??????00??????????00?????1???0?1??0???00?01?010?0?0??00??1?010?0????000?10?10001110??0??01000000???10010000????

Citipati_osmolskae ??1000000010100101?0?110100-00020111000----0000100101?0010100001000-1101100101?10-1111000100?0-001200-000?000000??010?011011111101?0??001?01010?1010112000000-0?001??11111110?0000021-0201101111100-110-10-0-1010001100-0111------1----------2------------??10-?110-?????210010??1??????1?0?10?????????1??100??0??0?1?52????0???10?????1???0??2?01?1????0??0010011111200????????00?0??1100?10?1?????1?000?0???000??0?1?10?00????0?????????0?22??0????0010000?????0-100????001?1???????0-01000?1010??0110???20?11102300102?000-01??????2110010?0?10?????0????????????????0000??000????001??0???00?00?0?0?0?0??000-10001????????0010110011000?000000?0?000??0?0?0?00?0000

Conchoraptor_gracilis ???0????0011100?0?00??1?????0?020??????-??????01????1????110??010?0-???????101?10-?1000001???0-?01??????0?000000??0?????????1??1?????????????0??????0?2???1?????00-??110????????0??21-0?01101??1100-110-1??0-?0?000??0???111-??-??1?--????-??2????-????????????????????????0????????????1???????????????????0??1??0????1?1??0???????1??????????????????????0????1???12?020000???00????0100??0?1????????00?0???0?????????????????0?????????0????0?????00100100????0-100?11?001?1???????0-10000?1010??00?1???20???1????010??000-0???????11?001??0?10?????0???????????????????0??????????01??0???00?00?0???0?0??0?????????????????????????????????????????????????????0000

Gigantoraptor_erlianensis ???????????????????????????????????????????????????????????????????????????????????????????????????????????????????????????????????????????????????????????????????????????????????20-0001111011100-110-1??0-00?00?0-????????????????-???-??-2------????????????????????????????????????????????????????????????????????????????101012?0000?0??0??????????1???????????????????1?00??0100???110?110002?0??00-00000000?????00010-10?000?????????????????????????????????????????????????????????????????????????????????????????????111110????10001??????????????????????????????????????1?????????????????????0?0-???0??????????0????-???00???0???0???000??????1?00?????

Incisivosaurus_gauthieri ??1000000101110100000100110-000111110001???0000100001100010000?1000-1001200001000-101000010000-00100100000000010010102011012101101-000001?0110???11?012001000-?0010?11??1100-??0?0?2011?01111111000-0-0000-0-00?0?0110??0?101200?00?00??1?2001101?10--10-?????????????????????????????????????????????????????????????????????????????????????????????????????????????????????????????????????????????????????????????????????????????????????????????????????????????????0?????????????????0??????????????????????????????????????????????????????????????????????????????????????????????????????????1???????????????????????????????????????????????????????????????

Microvenator_celer ???????????????????????????????????????????????????????????????????????????????????????????????????????????????????????????????????????????????????????????????????????????????????[12]0????11?1101100-????00-0-0????????????????????0???????2??2????-?--??????1101?110???????0010?01?1?11102??10100000?00100100010??0?10?0????????????1?11??00??2??????????????????????2002???????00?0??0?0??010110??02?00010000?000?0???????0????0????????????????2???0010?00001000-1001101001???1010000-00?00100100?0101???20????????0?????????1??11?10121010000100??000???11?001??001000000120001101??????????????????????????0-?????????????0????????????????????????????10???00??100

Oviraptor_philoceratops ???100??0?11?????1????10????0?0?0??10??-???0??01????1????01000???00-????1?0101?????1?0?0??0??0-001?0????0??0??00???10???1?1?1??1?1?0?1?????????????????????0????00-??????????????0?21-??011?1111100-110-10-0-?0?00???0????11-?????1?--??-?-??2????-?????????????????????????????????????????1??????????????0????????????????0???????????????????????10???????????1111??????????????0???1???1??0????????00?0???0????0???1????????0?????????0?????0????00?0000?????0-1???????0??1???????????????????????????????????????????????????????????????0?10?????0??????????????????????????????01?????????????????????????10011????????00101100110?0?0??000?0?000??1?0?0?00?????

Rinchenia_mongoliensis ???1?????011100?0?00??1?????????????0??-??????01????1????0100?010?0-10?????101?0????0?00?????0-?0??????00?000?00????????1???1??1???????????0???????????0???????????????????????????21-0?011?11111?0-??0-10-0-?0?0?0??0???111-??-??1?--??-?-??2????-?????????????????????????????????????????????????????????????????????????0??????????????????????1?????????????1??1??????????????0???1?0??0?0???????????0???0????????1?????????????????????????????00?000??????102???111?01?1???????????????????????????????????????????????????????????????0????????0??????????????????????????????0???????????????????0????????????????????????????????????????????????????????????

Buitreraptor_gonzalezorum ?????????0000??1?10???0?1???0??????????1???????0???????????00????0??????????????????1?10010??0-001?0????0???0?10?001???????????????????????????????????????0?????????????????????????1?0?0-?0????01010????????????????????????????0??01-0020???00?00??00-0??110?010-??201210110??12????010??????????0??1????0?????1?1??0?1??0???????1??01??10?2?01??????1??2???????102?1???????1100???101101001???????????0??1??????1?110???????????????????22???????????????0100??????????01?10?0????111?111?10????010????20112122??0112?0?1001???????1??1??????????????????????1????????11?20001????01?0011?10??1-01001?0???1??10011?00--00?0000110010000?1011??000000?0010?010000100

Deinonychus_antirrhopus ??000000200001?111101000?00-000000010001000000010000110??1000001010-0--1???100?110??10???10??0-00??0???1001000?0??11031?110100?0???????????1?????????00??0????0?0??????????10??????0011?00-?000??00-0-0001010?1001???110101011000002100?001000000?000100-0001111?10-00101200011?01011010020100000000000100101011101100??????0?????00120010010?1001?1????1?12?????1???21120000??110?0??1010?10?1?0???1000010001000?00?1110000110100100110011122000212?0010000001?00-1??1100001?1??0?0000-1110012010??0101???001111222001021010-0101011111111100001000001011111?00101??10?000002000110100110011?10?100011101001010-10001000--0001110?10111010?101001000000001100000000100

Dromaeosaurus_albertensis ????????2?0??1??1?1??100????0???0?010??10100??0????011???100??0101??????1???????0-1210111???????0??0?????????000??01031???0?0??0001000001?00000100?0?0011?01010000-1110-10-100000000011000-00000-00-0-000100-111010111101010?000110?000?00000000000?0100-0???????????0???????????????????????????????????????????0????????????????????????????????????????????????????????????????????????????????????????????????????????????????????????????????????????????????????????????1??????????????????????????????????????????????????????????????????????????????????????????????????????????????????????????????010-??????????????????????????????????????????????????????

Linheraptor_exquisitus ???000002000?1?1101?1?00?10-00000001000????000?0100???0001000101010-???1110100?10-1?1000?100?0-00?200-00001010?0?0?10????????0000?????????????????1?10101????????????????????????0??0??000-000???00-0-0?010101????0??111???01?????02101?0?1000000??00100-???1111010-1??00?0??11?11??1??????????????????????00???????10??????????10?0???000??001000?1101???1?1110??????????????1?????01?????10?11???01?0??1?????10??????????????????0???00????????2????????????1?0?????????????????????????????20????010????01??????????????????1???????????????????00?101??111??10???10???0??2000110??011001??10?10?0?10???01010-??????????????????????????????????????????????????????

Mahakala_omnogovae ???????????????????????????????????????????????????????????????????????????????????100?00???????????????????????????????????????0???????????????????1001??1???????????10?0-10?????????????????????????????????????????????????????????????????????????????????????????????????????????????????????????????????????????40?1??0???????1??01??10?2?????????1????????????????????????????????????????????????1???10??????1110?0???????????????????????????????????1000-0???0-??11?1???????0-11100???????????????????????????????????????1?11?111?0?0???0?0????????00??????0???00??0001????01?00??010?10-?1??0?0??010-??????????????????????????????????????????????????????

Microraptor_gui 100????01000???1??????00??0-0??????????????00000???0?????1?0?0??????????1?0??????????0????0??0-00??????????????????????????????0?????????????????????????????????????????????????????10?0???000??00-0-0?????????01???1????10???01?02?0??0?10000???00--10-?????????????????0?111??1??????00??0?1???????????100???0?1?004????1????10??1?001??1--1-01?1??????120100?1110?0?????????100???1111010?11???0????110??????000?110000000-10?10?0????0?22??02??1001000000100101?0?111??1?1???????0-011?1?200--?010?1-0011121?2??011011010?1??????11?111????10???0?0??????00??????0?0?0???0001???10110011?10?11?0??01?101010-10011?????0??0110010111010?00100100?110?01?0?0000??100

Saurornitholestes_langstoni ???????????????????????????????????????1??????0??????????1??????????????????????????1?111?????????????????????????????1?????????????????????????????????????????0???????????????0????1??00-?0?????????0????0-???????????1????1???0???0??0?1000000?000100-???1111010-1?1??200011?11?1011?12??000???00???1??10100110110?41?1010100????1??0???1??2?01?1????1??1?????????20??????????????????????0?????1???00?0?00?1?000??1?0?00?0-100???110011022?002???00100000?10?10210110100??1?0010011100100???????????????0???1????0?0??0??????????????????????????01011111???101??1?01100110001100101?0011?10?10?001?0?001?10-??????????????110??????11????????00?000??????0000?????

Sinornithosaurus_millenii ?000??001?000??1111?1000??0-00001?11??01???0?00010????0000000?010?0-0--1??0100??101110100100?0-001201101?0?0??0??0010??????????0??????????????0??????????????????????????????????0?101?000-00000-00-0-0?010100??0?0??1????101010000?10010?1?00000?000100-0???????????????????????11?????1????????????????????????????04?????0???????1??0??????2?????????????01001111?20120000???10?0??1011?10?????????????0????????0????0000????1?????????1?22??02?2?00?0000?????0-1???11??01?0???????0-11??0?2011?????1???011121????011211010?1??????????1????????????????????????????????0??1001???001?00???10??1?0???1?001?10-100???0???????000?????1010?10100100?110?0010?1?00?0?00

Tsaagan_mangas ??00000020000?011010?100110-000000010001???00000100?1?0001000101010-0--1100?????0-1110001100?0-001200-000010?000?00103????0?00000010010010010001011010101100???0000??10110-1000000?001?000-00000-00-0-0?0101011101011111?010101?0002101?0?1000000?000100-??????????????00?0?????110??01?????????????????????????????????????????????????????????????????????????????????????????????????1??????????????????????????????????????????????????????????????????????????????????????????????????????????????????????????????????????????????????????????????????????????????????????????????????????????????????????????????????????????????????????????????????????????????

Unenlagia_plus_Neuquenraptor ??????????????????????????????????????????????????????????????????????????????????????????????????????????????????????????????????????????????????????????????0??????????????????????1????????????????????????????????????????????????????????????0??????????????????????????????????????20?00101??????1??101??11011?0?0?1??0?0???????????????????????1???????????????????????110000?110?00100110211????????????????????????????????????????????????????00???01000-210011100111??010000-??111?20110101011-00011110??0011????11010?01?1112101000?10??00?????11??01??????????????????????1???????0????11??1??????????????????????????????????????????????????????????????

Velociraptor_mongoliensis ??00000020000?0111101100110-000000110001???000001000110000?00101010-0--1120100?10-1110110100?0-000?0???00?10?000?0010?1?1?0100?00??0010??001?00??1?01001?000??0?00-??11110-100000010011000-00000-00-0-000100-?1?0?0111101010?110000?00110010000000000100-0??111?010-???012000????10??0101???10?00????0?100101??1?01?0050?1010?0010001??010?1????01?110??1?1201000110020120000??100000?1110010?1?0??01?00010??1100??0?1?10000????00100?????0?220002?2?0010000001000-110011100101?00?1000-01100020110?01011-0001111022001021010-0111??1?11?11?000010000010?1???100???0????0000?20001???101?0011110010001111?001010-10011??????0?111?1100?101001011010??000?011000000?0100

Epidexipteryx_hui ?-?????????????????????????????????????-??????00???0???????0?001?00-???????????????0???00?0??0-001??10????????00???0????????????????????????????????????????????????????????????10?2?0?00???1?10-00-10001??0?01?00???????010?011100?01??2120?101?0?1??00-0??????0????????????????12????000????100??????????00???0????04???0?0?0?2100???020?1??2??100????????1?0??????2?1????????00??0100?1?01?02?????0?0010?0?0??001????????0???????????0???22??0??????00001?010?0-200011??????????0???????0??0?0??????????0?010102?????110????1????????????????????0????????????0?????????1??0001???111000110????0?0???0?????????0????????????0????????????????????????????0??0???????

Epidendrosaurus_ninchengensis ??????????????????????????????????????????????0????????????????????????????????????1???0????????01?????????????????????????????????????????????????????????????????????????????????????0011111?0-00-100?10-0?????????????????????????????1???1?1?0?1?????????????????????????????1???????0????1?????????????0?????0?1?????????????????????????1???????????1??????????2???????????????100?1?00??2?????????10?0??0?000????????0???1??01???0???22??0??00?000001???????????????????????????????????????????????????????????????????1????????????????????0??0?????????0???????????????????????001100??00???????0???0??10011?0??????00000100010000000000?0?000??0?0?0?00?????

Scansoriopteryx_heilmanni 0?????????????????????????????????????????????????????0???10?001000-???1???????1?????????0??????0????????????????????????????????????????????????????????????????????????????????0?20??00??01?11-00-100?10-0?0??1?0??0????1????????????????????????????????????????????????????????????0??0?0?100??????????00???0????030???00?0?10?0??001??10-0-??0???1??11??????????2??2000??1??????0?0?1?01??2???1?0?0?00???0000001?11000011010?10100?0?0?22000210000100000?10?0-2001????01?1?00????????????0?0-?????????[01]00??0?2?????1100???1??????2-???1????1????????????????0????????????????1???0??0011?00?00???00??101?0????????????????????????????????????????????????????????

Jinfengopteryx_elegans ????????1?01???1?10?0?????????001??????????????0???????????????????????????1??????????00????????0?0????????????2??0??????????????????????????????????100????????????????????????????0????0-?0??0-0????????????1??????1?????0?000??0???????????1???????0?????????1????????????????????????????????????????????????????0??????????10?????02????????0?011?????2?????????2?1????????1?????1010????????????????????????????110?0?????????????????22????????0?0?????????????????????1???????????????3????????????0?????????????????????????????????????????????????????0???????????????????????????????????????????????10011????????0??0110?110?0?11?00??0?00???1?????00?????

EK_troodontid ??????????????????????????????????????????????????????????????????????????????????????????????????????????????1????11??????????0????????0?????????????????10????1????1??0?????01?????????????????????????100-???0????1???????????????????????0???????1???????0-????????????????????????????????????????????????????????????????????????????????????????????????????????????????????????????????????????????????????????1000010-?0?1???????1?2200?2???0000??????????????????????????????????????????????0?????????????????????????????????????????????????????????????????????????????????0011?00?1????0???1???10-??????????????????????????????????????????????????????

Byronosaurus_jaffeei ??000000110000010001?100110-000001110001????00001000??0000???0????110--11101110?0-?0???????????????????????????????????????????000?11?0??1???0?00011?100111????010-??11001010111?????0?000-00000-0110-0?010???????????????101?001002201-002000120000--10-0??10-?????????2??0??????210???1??????????????1??1???????0????????????????????0???2???????????????????????????????????????????????????????????????????????????????????????????????????????????????????????????????????????????????????????????1???????????????????????????????1???????????0?0?01??11???????????0?????????????????????????????????????1????????????????????????????????????????????????????????

Mei_long ??00000011011?00?-??0?00?12100000?11000?????00001000??01--11?0?11?0-0--12?01110?0-1100?00??0???001100-0???????10???11??????????0?????????????????????110??????0???????????????0??0??????00-0000??0110-0???????100????1????10?0?0?001201-0020???0???0--?0-0??110000-?--101210110??1010??01?0?1?1000???00????00???00111040???1??0?1000?0001??2--0-0?0?1?0???12?????111120120000?1100000110100000010?111000110?0?????0???????????????10???00???2?000?1210010000001????????0--00??1?0011000-01100000????01001-0101??10???0??20??10?10?01??1110110?0?11?000?????????011?0?100??0112000110?00100011???1?1??10112101010-1??????????????001????????0???????????0??0????????01??

Saurornithoides_mongoliensis ??1000??1100000??10?01?0????0???0?01???01000???0?????????1??????????????110??????????0???????0-00??0????????????????????1?01????00?11?????????1?????????????0-0??0-????????0-?11?0?000?0?100000??011??0?0????????????1?????0?000??02101-0010001000001010-0???????????????????????1?????0?00??????0??000?00??0???000??0?0?10?000???001????00??????????????????????????????????????????????????????????????????????????????????????????????????????????0??????????????????????????????????????????????01?1???0??111023001020000-0???1111111011??????????????????????????????????????????01??????????1???0???1???1??????1?????????????????????????????????0???????????????

Sinornithoides_youngi ????????1?000???????????1???0?00??????????????00??????????????01????????????????0-??????????????0??????10?????????0???????????????????????????????????????????0???????????????1????000??00-?000??0110-0????????????????????0?000??0?101???1??0100?10?010-????????10-???0??101???????????1????????????????????????????0??????0???10??1??01??1??0??1?0????1?12?????1???20120000???00?0??1??0?01???????2??0?10????????????10?0010-?0?1?????????22??02????0?0000??????????????001?1???????0-1???0?00??????01???00?1210??00?0210?0-?1???????1?011???????00?????????00??????0???0???????????0100011?0?10????011?101010-100?1?????????0?????0???0??10?10000?000??010?0?00?????

Sinovenator_changii ??1???0001010??0010?1100111000?11???000??????000???011????????0?????????????????0-???0?00???????01????????00??12??011??????????00010110001--010??01101011110110010-?1110010101001??1?0??0???000??011100?0??0-?1?0?0??????0101?00??0?2?1?0?10?010??0?01?0-?????????10???0?210?10??01?????00???????0000?01??10000???0?1?40?1?00???10??1??01??2??0????????????1?????????20120000???10000?101?0????????????????????????0???10?001???????????????????????????0000???????????111????1???????0-11?00?2011??010????0011210??0011210010?10?01?11110110?0?11?00??11?111?0011???1000?10??0001???001??????00?10???1?1?1???1?????1?????????000?????????????0????????????????????????

Troodon_formosus ??0???001?0?0?010?011100?10-0000?1??0??1??????00???0????????00????110--????100??0-?00?0001??00-?01??0-0???0?0?1???01030??1?11??0?00110000???001??010?1000001???010-1110-10-10?111??1?1??010?100??0110-00?????????0????????101?00?00?201?0?1000220?001010-????111?10-111??210110?01?10???1001001????????1??101??1??0?1050?1?10?0????011?0?0?20?0??1?????????2??????????????????1?00000110??0?0?1?0?10???01?0????????0????000010-?0??0???00???22???????001?????????0-???????????????????????????0010??0001???0??11??23??1?21000-0?0001?11120110?0?1???0?00110101??1????1?0100012000110?001?????100111?1?011?1??010-??????????????????????????????????????????????????????

Zanabazar_junior ??10000011000001010101001110000001110001????00001000??1???1????????????????111?????00?000100?0-00120100?0000?0???????????????????001100?0????01??0??110001010-0010-??10-???0-?1110???0?001000000-0110-000????????????1100?1010001102101-0010001000001010-0????????????????????????????????????????????????????????????40?1010?00???0??00???2-?0?????????1?12????????????????????????????????????????????????????????????????????????????????????????????????????????????????????????????????????????????????????1??????0?????????????????????????????????????????????????????20001?????1???????????-?1?????????????????????????????????????????????????????????????????

Apsaravis_ukhaana ???????0????????????????????????????????????????????????????????????????????????????????????????0?????????????????1?????????????????????????????????????????????????????????????????0??001010011100-0-0??????????????????????????????????????2----??????????????110-1?001?12110??1?11???00??0?????????????????????0???50?1210?0?210?10-0200-??2?0110????????1?1001???3000-000111000001101001100?00011001110001010000111110001????110?11-0?1??????????????????11000-01010--0???1?1?1?000-10????3012--1---1-00000-00000-1-22000??11?11112-2001?????1100???????????11????????11?????110?111?00-?000110?11010?001000-??????????????????????????????????????????????????0100

Archaeopteryx_lithographica 1100001010010001010?1?00?00-000100010001???0000010001100111100021?0-0--1100111110-1101000??0?0-000000-010?00?000???0-210??12?010?0?0??????????0??0?01110001???0???????1001011?00??10010000-?0000-00-0-0?00-0-?1?0?00-0??001010001002001?212010000?00--10-0??1???0?0-??????10??????0?????????0??????????????00?????0??04?????0???20?01??01??1??2?00?0????1?111?00?1?102012-00001100?0001111?110020??1?000010????000?011?10?00?0-?0??01???0?1?220?0??2??0000000???00-010???1001?1??0????0-01100?20?1????11???0011210000010?2101001??????11?0110?0??0?00??0??????0010?????00?0002000110?11110011?00?00?0?010?0??010-10011??????0?00?0111111110011000100?10000010?0000?0100

Confuciusornis_sanctus 11-000-01?000000----0?01?0--00110011000????00000?0?01?11-111001-1?0-0???1?0????10-??00?0?10??0-000?0????0?00?000??1?????????0??0?????????????????????1?????0?????????????????????0?10??000-?0011000-100?0100-?1?000??0???011---?--1----------2----??------??????0??????????????????????0????0???0??????0??100?????1??05?????0???20?01??1-?0?????010110??????111001110?0????????1???00?11110110020??11001110001?1000011?11?00?0-?01?0????0?1?220?0?????000000?1?000-010????0?1?1???????0-??100?300---??1????0010-1?????1?211011?11?????2-20?1?????0??0??1??????00???????01??1??2110???111100??001?10?01??0?0??000-10011?????00?0110111110010010000000?000??1?0?0?00?0100

Rahonavis_ostromi ??????????0???????????0????????????????????????????????????????????????????????????????????????????????????????????????????????????????????????????????????????????????????????????????????????????????????????????????????????????????????????????????????????????????????????????????012011110000000?1001000010010?041?001?000??10120010010020??????1?1112??????????????????1100000110??0????2?????????10001010000?????????????????????????????????00??????01000-21011110011100010000-0111002011??00111--0011210000011121011010001112-20110000100000101111110011100100100012000110100110011011110101110?001010-??????????????????????????????????????????????????????

Sapeornis_chaoyangensis ??0000000?010??1?1??0???????0??????1?00?????00001000??????11-002?00-???12?0????10-11???0?100?0-0000?0-0????????????????????????????????????????????????????????????????????????????10-?001001010-00-0-0-00-0-?1?101??0??0010100?100?011-2120?2--------?0-0??????0??????????2??????211?????0??????0???????????????????05???01??0?210-0??1-0000??????0??????????????1112010-000?1100?0011011?11012?001?0011100?10000001111100010-10?10111?0?10220?020010010000001000-11010-???1?1??010000-0?100?30110101101-01010-10001011010011010?11112-2?11??00100?0????????????1????????11??1001???11100010??0?10??10?0?0??000-10011????????000001011100101???1??0?000?01?0???00?0100

Jeholornis_prima ????????????0??????????????????????1???????????0???0????????????????????1?0???????????????????????????????????1????0??????????????????????????????????????????????????100????????0?????000-?0?????????????????????????????????????1------?--?1?0????---0-0???????????????????????????????2??????????????????0?????????3????1????20?02??02?0?1??????0??????????????11?2??????????0???0?1011?1[01]?0??????????10??001?0??1?111?0?????????????0?1?220???????000000????00-110?10??0??1?????????????????????????????????????????????1??1???????????????????????????????????????????????????????1?????????????????????????10??0????????0000111010000?10?001???000?00?????00?0100

Yixianornis_grabaui ???100??2?0?0???????????1??????????????????????0????????????-????????????????????????????????0-?0?????????????????1?????????????????????0????????????????????????????????????????????0?0?0-10????00-????0?????1??0????????10100?101?--1--12??100?0??--10-011????110-???????0????????????02?????00???????????0?????0??07???00????21?????1-?000?2???11???-????1100001003000-00011100?0?110110110???000100??1000100000010111?0?????????????0???22????????01000??110?0-000?0-?????1???????0-1?1???3012--?????-?1?01210000???20?01?010?????2-201???????0??0???????????11????????1?1???????11??0010?????0???????0???0??10011????????0000011110001?1???1??????0??1?0???00?0100

Aorun_zhaoi ??00??00?10?110101001100??0-00010011100????00000?0000?2000100000000-0--1110110?1100??0???100?0-00??????010??0?0000?11????????0?0?????????????????????????????????????????????????0?10??010-00000-00-0-0?01?0-1??000??111?01010001002001-0010001100000100-0???????1101?101001?11010110000?00?00?00000000??0?000??00????????????????0010???????????????????????????????????????????????????????????????????1??0??00???1?10000110-10110111?0?0022101212101010101???????????????????????????????????1111???????????????????????????????????????????????00000???1000020?0??????000?100110?00100011?00000?01000?001000-1?001?01000??0000?10011000??0??0?000010?0010?01000????

Shishugounykus ?????????????????????????????????????????????????????????????????????????????????????????????????????????????????????????????????????????????????????????????????????????????????????????????????????????????????????????????????????????????????????????????????????????????????????????00?0?1???????????????????????30?1??0????0000000?00?0?????????????????????????????????1??0?00000????00110110??????1??????????????000110101000??????02200?21?101000001??0?????????????????001??100???????????????????????0000????????0-0101011000?000000?10100010111111002?10010000????????????0???????1??0??01???????0???1?0010010010?0000?10011010?100?01000000000101010000???

Haplocheirus_sollers ??00??0011011111010111001110000100111000?0?0000100001?01-1100000000-0--1000111?112010000-10000-00??00??010011000??010?00110100000021?0001?0001000010?0111??00-00110??????1?110??00?1011110-0000100100-0001111010001??02100101000100120110010001101000100-0??????00-?--101?00?10?10111?101001001??0100001??1??0??1?101030?00?0?0?[01]?001?00000?00?00000??1?01???????????1102000001111101000101100110110110100100?1100001?100000010101101110110022111212101010001010???????11??01002?000001001??000011010100??01001100000110110?0-01??0110001000000?1010001011110000?011010??000?20???1?10010?????00?00?010002001000-110010010010110001100110000100001000010000101010000100

Tugulusaurus_faciles ???????????????????????????????????????????????????????????????????????????????????????????????????????????????????????????????????????????????????????????????????????????????????????????????????????????????????????????????????????????????????????????????????????????????????????????????????????????????????????????????????0??00??????0?????????????????????????????????????????????????????????????????????????00?1111101????????????11?2???0?000?01??????????????????????????????????????????????????????????????????101000000??0000001010001011111???20????????001010?1101???????????????????????????????0?00100101100?????????????????00000000010101000????

Xiyunykus_pengi ????????????????????????????????????????????????????????????????????????????????????1010?????????????????????????????20???0?????00201101100111011110?00110001000011?????1?0110?0??1??????????????010??00010110100100-????????????????????????02???0????????????1?1[01]010001101010001?1?11000100010?0?11001001???1???1100????????????001000?000000??????????????????????111100000110210010010110010020001011?21101????????????????????????????????????????????????????????????????????????????????????????????????????????????????1????????????0000101000101111110020110??000?0??1?????1001?001100?0000010????0?0?0-??????????????????????????????????????????????????0???

Bannykus_wulatensis ????????????????????????????????????????????????????????????????????????????????????????????????????????????????????????????????????????1?01???????????????00-0001????????????????????1?????????????????01011????????????????????????????????????????????????????1[01]01??111010100?1?111????1????????????????????????????0??0?1????001??00???1?00?0100??0100????????????111100001?010010001012000002100101102110110?0?1?1100011111011101111100221112???01000111010???????????0010010????11000000?????????????????????????????????11000200010000000111000101111110020110??00000010?111?10010????1000000010002?01?????1001001001011000?1001101???00?0?000000000101010000???

Patagonykus_puertai ??????????????????????????????????????????????????????????????????????????????????????????????????????????????????????????????????????????????????????????????0????????????????????1?????????????????????????????????????????????????????????????????????????????10-10?0???1???0??????10?00??01?0?01???10000?11?10????3002011000??112100?002??2??????????????????0???1110-1010????????001????0??01101111002010011???????10?10??101?????????1??1112?????011?11010???????110???????00100111????0111001011001000???0???????????0-0???1??11120001000111000001111110?00111??00011020011101101?????1????00???????110?0-???0??0122?11????????????????????00001001??????10??101

Alvarezsaurus_calvoi ????????????????????????????????????????????????????????????????????????????????????????????????????????????????????????????????????????????????????????????????????????????????????????????????????????????????????????????????????????????????????????????????0????0?0???0???0?12??11000??00???000100??????0???0??0?30020????????121?000??00????????????1?????????????0-00001?02100?00??0??????????????????????????????????????????????????????????0?011?1101000-2102110001002100??011?1000???????????????????????????????????01?11?002001????????????????????00????????10010?11111001?????100000?01010?010000??????0????1????????????????????????0?????00010?1000000

Achillesaurus_manazzonei ???????????????????????????????????????????????????????????????????????????????????????????????????????????????????????????????????????????????????????????????????????????????????????????????????????????????????????????????????????????????????????????????????????????????????????????????????????????????????????0?2?01000??1121???00???????????0?0????????????????????????????????????????????????????????????????????????????????????????????????????010???????110?01102?00???1111???0???????????????????????????????????1?121002?0?????????????????????00????????100[12]001111?001?????1??0?0??10????????????????????????????????????????????????????????????????

Bonapartenykus_ultimus ???????????????????????????????????????????????????????????????????????????????????????????????????????????????????????????????????????????????????????????????????????????????????????????????????????????????????????????????????????????????????????????????????????????????????????0?00??0?0??0????1011?0111?0???????????????????????????????????????????????????1110-11101?01001?011?-???????????????????????????????????????????????????????????????????1????????110????????????????????2110??0110???00????????????????????????1????????????????00??????0????????????????????????????????????????????????????????????????????????????????????????????????????1210

Albertonykus_borealis ??????????????????????????????????????????????????????????????????????????????????????????????????????????????????????????????????????????????????????????????????????????????????????????????????????????????????????????????????????????????????????????????????????????????????????????????????????????????????????????0??????????????????????????????????????????????????????????????????????????????02101011??1?????????????????????????????????1?011?12???????????????????????????????????????????????????????????????????????????????????????000?111111001????????????????????????????????02-01????????0???????0????1???00??????????????????00?????00?11?-10????

Nemegtonykus_citus ?????????????????????????????????????????????????????????????????????????????????????????????????????????????????????????????????????????????????????????????????????????????????????????????????????????????????????????????????????????????????????????????????????????????????????????02?00100?00???0-01??0????00???01??????0??01210001000[12]0????0??0?01???????????1000-00001111001?010?-??????????????????????????????????????????????????????????????????0100?????20--00000210210010110???3????????????????????????????????1?0111????0000??011100001111111001110?10???111111101011010????0001?????000????0?0-???????????????????????????????????????????????????0??

Xixianykus_zhangi ???????????????????????????????????????????????????????????????????????????????????????????????????????????????????????????????????????????????????????????????????????????????????????????????????????????????????????????????????????????????????????????????????????????????????????0??2?01??0??????0-0100???00100050?2211?10???101???????????????????????????????????????????????????????????????????????????????????????????1???????????????????????????01000-?0020--0110??10110110111???31?---1---0100000-0000----220?10010010112--0010010111000011111110011???100101111111110?1110????0?00?2-?10?0??????????????????????????????????????????????????????????????

Qiupanykus_zhangi ?????????????????????????????????????????????????????????????????????????????????????????????????????????????????????????????????????????????????????????????????????????????????????????????????????????????????????????????????????????????????????????????????0-?-?0???0?10-??1????????????????????????????????????4002??????[01]?012100?0100?0??????????????????????????????????????????????????????????????????????????????????????????????????????????????0?????????0--?001????2100111????031????1---???00??????????????????1?011112--0010000111?0001101110001?100???1?101?1???1010010????000102-11000????0?0-??????????????????????????????????????????????????????

Ceratonykus_oculatus ??????????????????????????????????????????????????????????11?01-1?0????1110111?1120????0?100?0-00??????100??0?????10-??????????0?021????0?-001?????????????00-0000-?1??????????????10????????????0??0-0??????????????????????????????????????????????????????????0?????0???10??0?221??1???????????????????????????????????????????01?1??????????????????????1011?????1?00-2000??????????????0????10????????????????????????1???????????????0??11?2?????????????????????????????????????????????1???????????????????????????????1???????????0???????????????????????????????11211?110??010?????00102-?1011?0?????????0??0111?11????????????????????00?00001?????????0???

Parvicursor_remotus ??????????????????????????????????????????????????????????????????????????????????????????????????????????????????????????????????????????????????????????????????????????????????????????????????????????????????????????????????????????????????????????????????????????????????????????2?21200?00???0?0?0100?0010?????2??1?????0121???10?0??????????0???????????????????????????????????????????????????????????????????????????????????????????????????????0???????0--?????2?01?001111?00?3??---1---???0000-0000----1?00???1?011112--00100001110000111111100111001?0101112110110100100???000102-11011?011000-??????????????????????????????????????????????????????

Linhenykus_monodactylus ?????????????????????????????????????????????????????????????????????????????????????????????????????????????????????????????????????????????????????????????????????????????????????????????????????????????????????????????????????????????????????????????????10-1??0??110????1210????121212????????????00???0?10?0?[01]?20?10??[01]0?121?00???0020????????1???1011?0??????????????????????????????????????????????????1?10101111010111111-21003211?2???11011?120??????????????????????????????????????????????????????????????????0??1?1???0010???111?0???10111???11?????????112??0?1?1?01?00110?0002-?1000?0???0?????0?011111111001?--?????????????0000001100?10?-10?0??

Shuvuuia_deserti ??000000110000010101?101102100000?11000-???00000000011000-11001-1?100--12201110112020000-10000-001000-0100000000??10-??01?1200000021110101--01001011111?00100-0010-??11001011110001100?001000001000-0-0000-0-010011110??001????0??03201-212110202?10--10-0??10-???0-10100211110?1121011?102?01???000???0??10000?001?1050?20?1?00[01]0012100010100200101????1110101100???1000-00001112000000000230000101?11-002????110?11?101011110-011010112--0221112--11101121201000-10020--0111021012001111000?31?---1---1-00000-0000----22000-010011112--00100101???0??1???101?011??????101112110110100100011000102-11000?011000-010010111111110001110?1001?1000010000001100?11?-100000

Mononykus_olecranus ?????????????????????????????????????????????????????????????????????????????????????????????????????????????????????????????????????????????????????1100?????????????1001011??0??1???????????????????????????????????????????????????1-212?1??0????--10-0???????1-?--1010?11????1211?101021012001000000-?10100???1010???2??1?????0121?0?1??????????????????101100???1002000001?11001000000230000101111-0021000111011?1010111101011010111100221112???1?011?120?0?0-???????????????1?00111??00?31?---???????00?0-0000??-?????0-?10011112--0000010111000011011110011?11???1011??1101101001?0011000102-11000?010000-?100?01111011100?????????????????0000011100?10?-100000

Albinykus_baatar ?????????????????????????????????????????????????????????????????????????????????????????????????????????????????????????????????????????????????????????????????????????????????????????????????????????????????????????????????????????????????????????????????????????????????????????????????????????????????????????????????????????????????????????????????????????????????????????????????????????????????????????????????????????????????????????????01????????????1??12?0???????1?00??????????????????????????????????1????????????????????????????????11???10010?11?11111??11100011000002?110102010000-??????????????????????????????????????????????????????

Heptasteornis_andrewsi ?????????????????????????????????????????????????????????????????????????????????????????????????????????????????????????????????????????????????????????????????????????????????????????????????????????????????????????????????????????????????????????????????????????????????????????????????????????????????????????????????????????????????????????????????????????????????????????????????????????????????????????????????????????????????????????????????????????????????????????????????????????????????????????????????????????????????????????????????1????0???11?21101101??????????????????????????????????????????????????????????????????????????????????

Trierarchuncus_prairiensis ??????????????????????????????????????????????????????????????????????????????????????????????????????????????????????????????????????????????????????????????????????????????????????????????????????????????????????????????????????????????????????????????????????????????????????????????????????????????????????????????????????????????????????????????????????????????????????????????????????????????????0??????????????????????????????????1?011?12?????????????????????????????????3?????1??-???00????????????????????????????????????????????????????????????????????????????????????0??11????????????????0????0???00???????????????????0?????00?10?-10????

Dzharaonyx_eski ???????????????????????????????????????????????????????????????????????????????????????????????????????????????????????????????????????????????????????????????????????????????????????????????????????????????????????????????????????????????????????????????????????????????????????0?0??01200?00???0??????????10???????????????1??0????1??2????????????????????????????????????????????2????0101111100?????11??1??????111?0101111??????0?211?2???1?01???2?????????????????????????????????31???????????????????????????????????????????????????????????????????????????????????????1??????0?1?2????????1?0?0????0?00111111100?????????????????0000000100?10?-10????

Kol_ghuva ?????????????????????????????????????????????????????????????????????????????????????????????????????????????????????????????????????????????????????????????????????????????????????????????????????????????????????????????????????????????????????????????????????????????????????????????????????????????????????????????????????????????????????????????????????????????????????????????????????????????????????????????????????????????????????????????????????????????????????????????????????????????????????????????????????????????????????????????????????????????????????001?0011000002-?11112010000-??????????????????????????????????????????????????????

Khulsanurus_magnificus ?????????????????????????????????????????????????????????????????????????????????????????????????????????????????????????????????????????????????????????????????????????????????????????????????????????????????????????????????????????????????????????????????0?????0????0??0?[12]2???????????????????????????????????????????????01?????00?0??0?????????????????????1000?000?1?11?0100?000?30???101????????????????????????????????????????????????????????????????????????????????????????????11?10110????????????????????????????????????????????????????????????????????????????????????????????????????????????????????????????????????????????????????????????0??

Ondogurvel_alifanovi ???????????????????????????????????????????????????????????????????????????????????????????????????????????????????????????????????????????????????????????????????????????????????????????????????????????????????????????????????????????????????????????????????????????????????????????????????????????????????????0??????????????????????????????????????????????????????????????????????????????????????????????1?10111???01?2101121?0?211?2?????????????????????0??????????????10??????3?0???1??-0100000?0000???????????1?????12--001????????000?1??11100?100010????1??????????1100??1??0??????0?????0???????0??1111?111???????????????????00000111?????????????

MPCN-PV_738 ?????????????????????????????????????????????????????????????????????????????????????????????????????????????????????????????????????????????????????????????????????????????????????????????????????????????????????????????????????????????????????????????????????0?01???0????1211?1??00??0?0?0???????1???1????????30020?1?????112100?00?00??????????0????????????1110-1110???1001?011?????????????????????????????????????????????????????????????????????????????????????????????????????2110??0110???00??????????????????????121???0????????????00??????0??????????????????????????????????????1?????????0???????????????????????????????????????????????????1210

;

end;

ctype ord: 47 74 82 99 118 124 131 151 180 184 222 228 229 235 238 239 265 266 274 275 282 283 287 311 312 321 329 332 334 335 348 351 358 380 381 384 386 389 395 429 430 434 436 441 443 445 466 468 479 505 506 519 521 558 579 586 603 ;
